# A subgraph isomorphism algorithm and its application to biochemical data

**DOI:** 10.1186/1471-2105-14-S7-S13

**Published:** 2013-04-22

**Authors:** Vincenzo Bonnici, Rosalba Giugno, Alfredo Pulvirenti, Dennis Shasha, Alfredo Ferro

**Affiliations:** 1 Dept. Computer Science - University of Verona, Verona, 37134, Italy; 2 Dept. Clinical and Molecular Biomedicine - University of Catania, Catania, 95125, Italy; 3 Courant Institute of Mathematical Sciences - New York University, NY 10012, USA

**Keywords:** Subgraph isomorphism algorithms, biochemical graph data, search strategies, algorithms comparisons and distributions

## Abstract

**Background:**

Graphs can represent biological networks at the molecular, protein, or species level. An important query is to find all matches of a pattern graph to a target graph. Accomplishing this is inherently difficult (NP-complete) and the efficiency of heuristic algorithms for the problem may depend upon the input graphs. The common aim of existing algorithms is to eliminate unsuccessful mappings as early as and as inexpensively as possible.

**Results:**

We propose a new subgraph isomorphism algorithm which applies a search strategy to significantly reduce the search space without using any complex pruning rules or domain reduction procedures. We compare our method with the most recent and efficient subgraph isomorphism algorithms (VFlib, LAD, and our C++ implementation of FocusSearch which was originally distributed in Modula2) on synthetic, molecules, and interaction networks data. We show a significant reduction in the running time of our approach compared with these other excellent methods and show that our algorithm scales well as memory demands increase.

**Conclusions:**

Subgraph isomorphism algorithms are intensively used by biochemical tools. Our analysis gives a comprehensive comparison of different software approaches to subgraph isomorphism highlighting their weaknesses and strengths. This will help researchers make a rational choice among methods depending on their application. We also distribute an open-source package including our system and our own C++ implementation of FocusSearch together with all the used datasets (http://ferrolab.dmi.unict.it/ri.html). In future work, our findings may be extended to approximate subgraph isomorphism algorithms.

## Background

Complex biological systems arise from the interaction and cooperation of a large number of molecular or organismal components. Understanding such systems has required, just at the molecular level, the construction and analysis of protein−protein interaction, metabolic interaction, transcription factor binding, and hormone signaling networks. Networks are represented by graphs, where vertices are, for example, molecular components and edges represent some relationship among them. Understanding such networks mainly requires finding specific topological subgraphs, which entails the application of subgraph isomorphism algorithms [1-3]. Such subgraphs are sometimes called network motifs [4]. These motifs, which could be repeated in the same network or in different networks, give insight into evolutionary mechanisms (analogous to the process of establishing the evolution of proteins through local alignments of sequences). In [5,6], the authors find network motifs through the following steps: (i) enumerate all possible subgraphs of the network; (ii) classify them in classes of isomorphic subgraphs; (iii) generate random graphs and enumerate and classify all subgraphs in such graphs (i.e. null hypothesis construction); and (iv) establish as motifs all subgraphs classes that appear with higher frequency in the real network than in the random networks. The second step is repeated many times in real and random networks and entails the usage of subgraph isomorphism algorithms [1]. In [7], the authors provides a Cytoscape plugin to query networks by drawing and searching known or user-defined subgraphs. It uses the subgraph isomorphism algorithm of [3]. The authors in [3] and related publications show their speed-up compared to the algorithm in [1] which is used in [5,6]. In another application, molecular components, such as small or large proteins, are represented as graphs. In such chemical networks, vertices are atoms and edges are the bonds among them. Systems such as Daylight [8] and its academic version Frowns [9], collect a set of molecules represented in two dimensions. Then, given a subgraph, they apply a subgraph isomorphism algorithm [3] to determine how many times and where the subgraphs occur in each molecule of the collection. The aim of the above works is to predict or increase the functionality of new or known molecules. In [10], graphs are used to represent proteins in three dimensions. There, vertices and bonds are associated with their positions in space or contact maps are used. Contact maps represent protein residues and the cut-off distances among them starting from a three dimensional protein structure. The authors discuss subgraph isomorphism algorithms, graph theoretical properties and the importance of an efficient implementation of such algorithms with the aim of detecting ligands that bind to proteins (i.e., common regions in the maps). Finally, in [11], the authors describe the relations among the components and subcomponents of molecules by using hierarchical graphs and making use of subgraph isomorphism algorithms [1] to find common substructures.

Finding a solution for the subgraph isomorphism problem is inherently hard [12] and therefore the efficiency of any software using subgraph isomorphism algorithms largely depends on (i) finding efficient heuristics to make the isomorphism algorithms faster; (ii) reducing the number of subgraph isomorphism calls; and (sometimes) (iii) relaxing the isomorphism conditions.

Graph indexing based methods aim to design efficient indexes (i.e. extracted from graph subgraph, trees or paths [13-18]) or data structures [19,20] capable of limiting the execution of subgraph isomorphism to only a few candidate graphs; graph mining algorithms [21-24] reduce the size of indices by identifying frequent subgraphs having at least a specified support; and graph pattern matching algorithms [25-28] solve a "near" subgraph isomorphism problem by applying more relaxed reachability conditions [27].

This paper introduces a new algorithm for the subgraph isomorphism problem and compares it on synthetic and biochemical data with the most efficient and recent algorithms present in literature [3,29,30]. Notions, concepts and related work are given next.

### Basic notions

A graph *G *is a pair (*V*, *E*), where *V *is the set of vertices and *E *⊆ (*V *× *V *) is the set of edges. Let *A *be a set of labels, the functions *lab *: *V *→ *A *and *β *: *E *→ *A *assign labels to vertices and edges, respectively. If (*u*, *v*) ∈ *E*, *v *is called a neighbor of *u*. Given *G*, |*V *| (|*E*|) indicates the number of vertices (edges). A graph *G *is *dense *when the ratio |*E*|/|*V*| is high, *sparse *otherwise.

Given a pattern graph *G *and a target graph *G'*, the problem is to find an injective function, *M *: *V *→ *V'*, mapping each vertex of *G *to a unique vertex of *G' *such that the following isomorphism conditions are satisfied: if (*u*, *v*) is an edge in *G*, *u *has label *lab*(*u*), *v *has *lab*(*v*), then the corresponding edge (*u'*, *v'*) in *G' *has *lab*(*u*) = *lab*(*u'*), *lab*(*v*) = *lab*(*v'*), and *β*(*u*, *v*) = *β*(*u'*, *v'*). Note that there may be an edge (*u'*, *v'*) is ∈ *E' *without any corresponding edge in *E*; when this happens, the subgraph isomorphism is also called a *monomorphism*. When *G *has exactly the edges that appear in *G' *over the same vertex set, then *G *is an *induced subgraph *of G'.

In what follows, we view *G *and *G' *as connected graphs and ignore edge labels (edge labels improve efficiency, because they add more constraints, but complicate the algorithm needlessly). Moreover, we consider graphs that are directed, that is (*u*, *v*) ∈ *E *does not imply that (*v*, *u*) is also in *E*. Our approach applies as well directly to undirected connected graphs. When needed, we denote an undirected edge with 〈*u*, *v*〉.

### Algorithmic aspects of subgraph isomorphisms methods

A simple enumeration algorithm to find all the subgraph isomorphisms (i.e., occurrences) of a pattern graph in a target graph works as follows: generate all possible maps between the vertices of the two graphs and check whether any generated map is a subgraph isomorphism (which we will call a match). Whereas this algorithm is inefficient if done naively, it serves as a good starting point.

**Figure 1:**
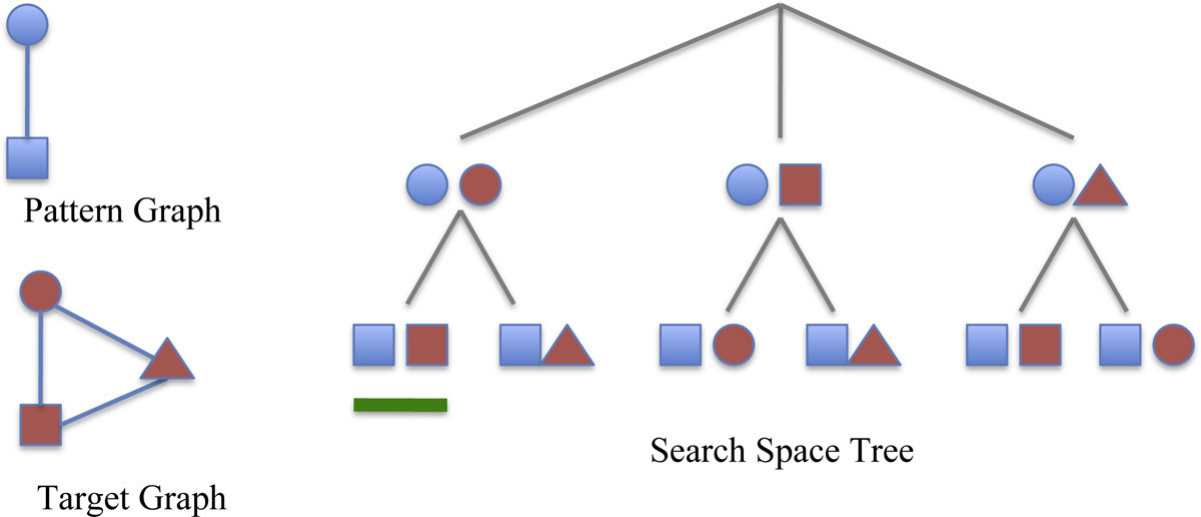
**Search space tree**. The leaves of the search space tree corresponding to a path of the search tree leading to an isomorphism are highlighted with a green stick.

All the maps can be represented using a *search space tree*. The tree has a dummy root. Each node represents a possible match between some vertex *u *of the pattern *G *and some vertex *u' *of the target graph *G' *. The path from the root to a given node represents a partial match between *G *and *G' *. Only certain leaves correspond to subgraph isomorphisms between the pattern and the target graph (see Figure 1 for an example of a search space tree). During the visit, the isomorphism conditions are applied to verify the partial matches. When the conditions are not satisfied the algorithm prunes the underling branches and backtracks on the parent nodes of the search tree. The size of the above search space tree increases exponentially with the graph size. Because the subgraph isomorphism problem is NP-complete [12] (to be precise, subgraph isomorphism of graphs with repeated labels or no labels is an NP-complete problem; such graphs are typical in biomedical applications) a cheaper-than-exponential algorithm may not exist. Next we sketch the main heuristics in existing subgraph isomorphism algorithms. The common aim is to eliminate unsuccessful mappings as early as possible.

**Search strategy **An important factor influencing an algorithm's performance is the choice of a good variable ordering (sometimes called a search strategy) of the pattern graph vertices in the branches of the search tree. For example, a variable ordering may begin with a pattern vertex having the highest degree or having the most uncommon label in the target graph [31,32]. A strategy depending on the partial solution could choose the next pattern vertex to be matched such that the number of children in the current search tree's branch is minimized [29]. One can choose to maintain the same variable ordering for all the branches of the search tree or can choose different orderings for different branches. These two strategies are called *static ordering *and *dynamic ordering *[29,33], respectively.

An important difference between static and dynamic orderings is that the first one can be chosen *a priori*, before the search phase. Dynamic strategies must be elaborated during the search.

**Reduce the search space **After evaluating a partial solution, an algorithm may backtrack if there is no possible mapping for the remaining unmatched vertices [3]. Alternatively inference-based techniques can predict future branching of the search tree thus avoiding the need to explore partial solutions that do not result in a match [29,30]. An intelligent matching algorithm orders vertices well and filters well. However, intelligence often comes at a cost.

### Related work

Next, we briefly describe the state of the art. We refer to [3,29,30,34] for a deep treatment of the subject.

A popular algorithm, VFlib, was presented by Cordella et al. in [3]. It uses a dynamic search strategy. Given a partial solution, first it chooses unmatched pattern vertices having edges starting from vertices in the partial solution; then it chooses those unmatched vertices having edges ending in vertices in the partial solution. In order to reduce the search space, the approach uses the following two lookahead heuristics. A mapping pair (*u*, *u'*), where *u *and *u' *are vertices of the pattern and target graph, respectively, is considered a valid match if it satisfies the following rules: (i) *u *and *u' *are both neighbors of matched vertices; (ii) the number of unmatched pattern vertices which are neighbors of matched vertices and are connected with *u *must be less than or equal to the number of unmatched target vertices which are neighbors of matched vertices and are connected with *u'*; (iii) the number of vertices connected with *u *and not in (i) and (ii) must be less than or equal to the number of vertices connected to *u' *that are not in (i) or (ii). The rule (ii) is subdivided into four cases depending on the direction of involved edges between the neighbors of *u *and the set at (i).The rule (iii) applies only for induced subgraph isomorphism as opposed to monomorphisms.

Subgraph isomorphism may be modeled as a constraint satisfaction problem (CSP). Given a set of variables (pattern vertices) and a set of constraints among them, a solution of a CSP for the subgraph isomorphism problem consists of finding an assignment of values (target vertices) to all variables such that all constraints are satisfied. Initially, each pattern variable *v *is associated with a set of values formed by the set of target vertices that could be matched to *v*, i.e. *lab*(*v*) = *lab*(*M*(*v*)) and the degree of *v *is less of degree of *M*(*v*). That set is called the *domain *of *v*. Constraints guarantee that isomorphism conditions are maintained.

Several filtering techniques, such as forward-checking [34], prune the branches of the search tree by propagating constraints to remove values from potential domains (i.e., domains of variables not yet assigned).

A branch is pruned when a domain becomes empty. In forward checking, first a variable is assigned, then all constraints involving such variables are propagated to remove values from other domains that are not consistent with the current assignment. This is called inference.

Solnon in [30] proposes LAD, which combines the constraints that two pattern vertices cannot be matched with the same target vertex into a partial solution, together with the preservation of edge correspondence between the pattern graph and the target graph. Such constraints are applied during backtracking and are propagated until convergence (i.e., as much as possible). LAD defines a dynamic search strategy where the next pattern vertex to be assigned is the vertex with the smallest domain cardinality.

Recently, Ullmann proposed an algorithm called FocusSearch [29]. The search process is done by a backtracking algorithm that applies a bit-vector domain reduction to each step. Before the search starts, it runs two preliminary steps. The first one, called prematch, fills domains by filtering them using vertex invariants based on labels and topology. The second one locally ensures that two pattern vertices cannot be matched to the same target vertex. After the preliminary steps, a static search strategy orders the pattern vertices in the following way: each pattern vertex with a single compatible target vertex is put at the head of the sequence and the next pattern vertex to be matched is the one with the highest number of branches between it and the partial solution. If there are two vertices that are equal candidates to be the the next vertex in the ordering, it chooses the one with the highest sum of the degrees of its neighbors.

Other methods in the literatures do not rely on backtracking or CSP techniques, but rather apply heuristics based on probabilistic functions, explicit enumeration of matches, and so on [35-46].

### Contribution

Inference-based methods, which propagate constraints until convergence (for example LAD), reduce the search time to the greatest extent. Unfortunately, such inference is done at the price of a greater computational cost. On the other hand, when constraint verification is applied only locally (for example, the local inference used by FocusSearch and the pruning rules of VFlib), it is crucial to define a search strategy that tries to prune the search space as much and as early as possible at low cost. This aspect is not addressed by VFLib. FocusSearch applies this concept only partially. It defines a static and partly target-dependent search strategy reflecting the pattern topology. It also performs local inference, minimizing the cost by using bit-vectors. In this paper we present a novel subgraph isomorphism algorithm, called RI (http://ferrolab.dmi.unict.it/ri.html). It creates a search strategy based only on the pattern graph topology. The order is chosen to create constraints as early as possible in the matching phase. Roughly, vertices having high valence and that are highly connected with vertices previously present in the ordering tend to come early in the final variable-ordering. During the matching phase, RI does not apply any computationally costly pruning or inference rules. This is the first paper that compares all the most recent and used algorithms (LAD, FocusSearch, VFlib). We analyze algorithmic aspects including the size of search space, the memory requirement, the timeout of the algorithms, the matching time and the total time, varying the density and dimension of pattern and target graphs, the number and the distribution of the labels. Dataset characteristics are typical of molecular biological data. We also used the synthetic data analyzed in the previous work by authors of LAD and VFLib. In order to validate our strategy, we compare RI and two versions of RI, called RI-Ds and RI-DsPm. RI-Ds computes, after defining the variable order of pattern vertices and before the subgraph isomorphism starts, an initial domain assignment. For each pattern vertex, RI-Ds computes its domain and verifies that pattern edges are compatible in the target domains. It does not apply inference or domain reduction during backtracking. This low-priced verification helps in large dense targets, because it reduces the number of candidates to be verified during backtracking. RI-DsPm, in addition to RI-Ds, uses the prematch phase defined in FocusSearch, i.e. filters domains by using vertex invariants based on neighbor labels and topology. We show that RI-DsPm does not improve performance compare to RI and RI-Ds. This behavior is supported by the analysis given in [29] (see in [29] Section 7.7 "Molecular graph retrieval experiments"). Moreover, it validates the main ideas in RI: a powerful pattern vertex ordering, i.e. strongly dependent only on pattern graph topology, together with light constraint verification, is more efficient than a local or global inference procedure.

## Results and discussion

**Compared software **We compared RI, along with variants RI-Ds and RI-DsPm with VFlib (using the last released version named VF2), LAD and FocusSearch measuring the search space size, the matching time, the memory requirements and the total time.

RI, RI-Ds, RI-DsPm, VF2 and LAD are implemented in C/C++. Since FocusSearch has been released in Modula2, in order to compare the algorithms under the same platform, we re-implemented FocusSearch in C++ following the author's guidance and the original source code.

Next we describe the pattern and target graphs used to test the algorithms. Table 1 reports the statistics on the number of vertices, edges and labels of the real target graphs described below. We refer to the Additional File 1 for details on the synthetic datasets. We consider all graphs to be directed. We transform undirected graphs into directed graphs by replacing each edge connecting two vertices with two edges.

**Table 1:** Statistics of biochemical datasets.

	Min Vertices	Min Edges	Max Vertices	Max Edges	Avg (SD) Vertices	Avg (SD) Edges	Avg (SD) Degree	Total Labels	Avg (SD) Labels
*AIDS* Small Sparse	4	8	245	500	44.98 (21.68)	93.91 (45.05)	4.17 (2.28)	62	4.36 (0.86)

*PDBSv1* Large Sparse	240	480	33067	61546	5663.6 (6954.82)	86661.27 (12365.7)	3.21 (2.52)	14	5.9 (1.04)

*PDBSv2* Medium Sparse	1683	3414	7979	16302	3614.1 (1772.06)	7386.2 (3814.08)	4.08 (17.47)	13	4.63 (0.76)

*PDBSv3* Small Dense	7	16	883	18832	376.86 186.66	8679.48 3814.08	44.78 (17.47)	21	18.86 (3.48)

*Graemlin* Medium Dense	1081	12961	6726	230468	3167.6 (1568.66)	87759.6 (75939.2)	48.14 (63.61)	31676	3167.6 (1568.66)

*PPI* Large Dense	5720	51464	12575	332458	7827.1 (2120.15)	107135 (82730.9)	28.66 (47.44)	78271	7827.1 (2120.15)

Statistics of the number of vertices and number of edges. These describe the minimum, maximum and average number of vertices and edges in the dataset. Total Labels is the total number of labels in the dataset. Avg Label is the average number of labels per graph. Standard deviations are reported in parentheses.

Patterns are searched against all graphs of the dataset, by a one-to-many approach (pattern/target dataset). See the Table 1 in Additional File 1 for the average (and standard deviation) of the number of subgraph isomorphisms obtained per datasets.

**Molecular dataset ***AIDS *dataset contains the topological structures of 40000 chemical compounds that have been tested for evidence of anti-HIV activity (available from NCBI [47]). Compounds are graphs where the number of vertices varies from 4 to 245. They are small sparse graphs. Since the *AIDS *dataset contains relatively small graphs, the patterns are graphs of the dataset. Patterns were divided into four groups, each group has one hundred graphs, and each graph may have 4, 8, 16 or 32 vertices. The topology of patterns was chosen in order to respect the average degree and label distribution of the target graphs. This implies that patterns were often quite complex. Patterns of the same size may have different number of matches (this is shown in Table 1 of the Additional File 1, based on the standard deviation values associated with the number of matches).

**Protein dataset ***PDBSv1 *dataset contains 30 graphs with data from DNA, RNA, and proteins having up to 33067 vertices. Original structures can be downloaded from http://www.fli-leibniz.de/ImgLibPDB/pages/entry_list-all.html[48] and http://www.rcsb.org/pdb/home/home.do[49]. In our software package we include the software to convert the original data into graphs. Our software makes use of the BALL library available at http://www.ball-project.org. The dataset mostly contains large sparse graphs. Pattern graphs were extracted from the corresponding target graphs fixing the number of wanted edges. Patterns are subgraphs (monomorphisms) of their corresponding target graphs. We create six groups of 10 random patterns having a number of edges equals to 4, 8, 16, 32, 64, and 128. We generated the patterns from the original targets in the following way. Starting from an edge, the algorithm adds its closest edges to a list of candidate edges. Then, it choses a candidate edge, adds it to the pattern and then adds its neighbors to the candidates list. The process is repeated until the desired number of edges is reached. Again, patterns reflect the average degree and label distribution of the target graphs. The number of subgraph isomorphisms for patterns of size 64 and 128 is larger than for smaller patterns sizes (see Table 1- Additional File 1). This may due to the fact that patterns match parts of the backbones and parts of protein surfaces. Protein surfaces are rich in atoms of the same type such as hydrogens, leading to an increased number of possible subgraph isomorphisms.

**Protein backbones dataset ***PDBSv2 *dataset contains 40 proteins represented by the backbones of the proteins coming from the crystallography downloaded from *Jena *[48] and *Protein Data Bank *[49] converted to graphs by BALL library (available at http://www.ball-project.org). They are medium sparse graphs. They may have from 1683 to 7979 vertices per graph. Pattern graphs were extracted from the corresponding target graphs as we have done for dataset *PDBSv1*. We create seven groups of 10 random patterns, reflecting the typology of the target graphs, having a number of edges equals to 4, 8, 16, 32, 64, 128 and 256. Again, the number of subgraph isomorphisms for patterns of size 64 and 128 is larger than for smaller patterns sizes but is much smaller compared to the corresponding results in *PDBSv1 *(see Table 1- Additional File 1). This is due to the fact that the graphs are protein backbones.

**Proteins contact maps dataset ***PDBSv3 *dataset contains 50 contact maps of the amino acids of the domains of the proteins, retrieved by *CMView *[50]. While for backbones we can have thousands of vertices corresponding to atoms, the number of vertices in the contact maps is relatively small (corresponds to the length of the proteins), since they represent relationships among amino acids. Contact maps are small dense graphs. In average a graph may have 380 vertices.

Since target graphs are dense, we extracted from them different types of pattern graphs (from dense to sparse) to vary the performance comparisons. Patterns were generated from the corresponding target graphs giving the number of desired edges. Dense patterns are constructed by forcing the number of vertices to be approximately equal to 25% of the number of edges. Since patterns are subgraphs (monomorphisms) of their corresponding target graphs each pattern tends to reach the desired percentage of vertices. Semi-dense patterns have a number of vertices almost equal to 50% of the number of edges. For the sparse patterns the percentage of vertices is set to 90% and cycles avoid simple structures as paths. For each density of patterns, we create seven groups of 10 random patterns having a number of edges equals to 4, 8, 16, 32, 64, 128 and 256. Dense patterns have an average degree of 11.5 with a standard deviation equals to 3.74, an average number of labels equals to 9.94 with 6 as standard deviation. Semi-dense patterns have an average degree of 7 with a standard deviation equals to 3.04, an average number of labels equals to 10.24 with a standard deviation 6.33. Sparse patterns have an average degree of 4.4 with a standard deviation equals to 2.69, an average number of labels equals to 12.88 with a standard deviation 5.67. As we expected, dense patterns have fewer subgraph isomorphisms. The number of matches increases with the size of semidense patterns. However, this is not always true (see Table 1- Additional File 1). Sometimes, larger patterns may have fewer matches. In real data, this depends on the nature of the data.

**Graemlin dataset **This dataset contains 10 microbial networks (*Campylobacter jejuni*, *Caulobacter crescentus*, *Helicobacter pylori 26695*, *Salmonella typhimurium LT2*, *Synechocystis PCC6803*, *Vibrio cholerae*, *Escherichia coli K12*, *Mycobacterium tuberculosis H37Rv*, *Streptococcus pneumoniae TIGR4*, *Streptomyces coelicolor *) [51]. Since each vertex has a unique label, we perform the test using networks without labels, with unique labels, and varying the number of assigned random labeled from 32, 64, 128, 256, 512, 1024, to 2048. For subgraph isomorphism algorithms, the former corresponds to the hardest and the easiest cases, respectively. In [52], authors prove that the complexity of subgraph isomorphism algorithms is quadratic in the number of vertices on graphs labelled with unique labels. Labels are assigned using a uniform distribution. As described for *PDBSv3 *we create sets of 20 dense, semidense and sparse patterns for each pattern dimension (4, 8, 16, 32, 64, 128, to 256). The number of matches reflects the density but is not exactly proportional to the the pattern sizes (see Additional File 1, plots are shown for pattern dimensions varying the number of labels and density).

**Protein**−**Protein interaction networks dataset **This dataset contains 10 networks describing the known and predicted protein interactions downloaded from STRING [53]. We used the following organisms: *Mus musculus, Saccaromyces cerevisiae, Caenorhabditis elegans, Drosophila melanogaster, Takifugu rubipres, Danio rerio, Xenopus tropicalis, Bos taurus, Rattus norvegicus*, and *Homo sapiens*. They are large dense graphs. Since each vertex has a unique label, we perform tests using the networks with unique labels and then with randomly assigned labels varying the number from 32, 64, 128, 256, 512, 1024, to 2048. Labels are assigned using a uniform distribution and a normal distribution (gaussian). We extracted from the target graphs sets of 20 dense, semidense and sparse patterns for each pattern dimension (the number of edges vary from 4, 8, 16, 32, 64, 128, to 256). Again, the number of matches depends on the density factor but is not exactly proportional to the pattern sizes (see Additional File 1 where plots are shown for pattern dimensions varying the number of labels and density). In the Additional File 1 we present an application of subgraph isomorphism on protein complexes searching on GO annotated *PPI *dataset.

**Synthetic dataset **RI has been compared also on the synthetic dataset distributed by Sansone et al. [54]. They are pairs of unlabeled graphs having sizes varying from 20 to 1000 vertices. The dataset contains the following kinds of graphs: *Bounded Valence*: (the number of edges per vertex varies from 3, 6, 9), *Mesh *(2D, 3D and 4D, where 2,3,4 indicate the dimensionality of the meshes), and *Random *(edges are added according to a fixed probability; edges are independent and the probability distribution is uniform). Since the synthetic dataset is composed of a pair of (target and pattern) graphs, we do not need to generate patterns for it. We refer to [54] for the detailed statistics of this dataset and to Additional File 1 for the performance results on the compared softwares.

**Performance **Experiments have been conducted on a QuadCore Intel Xeon 2.33 Ghz, with 4 physical cores at 64 bit with 4 Mb cache, 4 Gb of RAM, running Linux version 2.6.32. We do use multi-threading. Experiments were repeated to get rid of caching effects. We set a timeout of 3 minutes to the total execution time of the algorithms (note that all algorithms end). We chose this timer since it reflects in proportion the results reported in [30] (where test are run with a timeout of 1 hour). For each dataset we report how many subgraph isomorphism runs each algorithm completes before the timeout. When an algorithm times out, we exclude the related running times from the means of all algorithms. RI shows the best behavior on all datasets. Results for VF2 on *PPI *datasets are not reported since they often time out. FocusSearch times out on dense datasets and LAD times out on large graphs.

Here, we report the average and standard deviation of space size, memory requirement, matching time and total time on biochemical datasets (see Additional Files 2, 3, 4, 5, 6, 7, 8, 9). All detailed comparisons and plots are given in the Additional File 1. In Additional Files 2 and 3 we give a comparison of all tested algorithms on *AIDS *and *PDBS *datasets for time and space respectively. Additional Files 4 and 5 give the performances on synthetic datasets provided by Sansone et al. Additional Files 6, 7, 8, 9 present a comparison of microbial and protein interaction networks varying the number of labels. For each dataset, tests are grouped with respect to the pattern density, number of labels or labels distributions as shown in the plots. We highlighted in bold the algorithm outperforming the others.

The total time needed by an algorithm includes the time to read graphs from files, to build data structures, to run preprocessing operations, to run the real matching phase and so on. Therefore, we distinguish between the total time and the matching time. The matching time for RI and VF2 pertains only to the matching process; conversely for RI-Ds, RI-DsPm, LAD and FocusSearch it also includes the preprocessing time. Notice that the preprocessing steps are the first parts of the matching processes and they depend on the pattern graphs. The space size is the number of visited nodes of the hypothetical search space tree and the memory size is the number of kilobytes required to store all data structures.

Beside total time comparisons, we analyzed space, and memory size due to the fact that comparing the performance of those algorithms means to deal with basic differences that may influence the final results and their applicability on a variety of data.

RI, RI-Ds, RI-DsPm, VF2, and FocusSearch store data in adjacency lists. LAD uses adjacency matrices. The formers use less memory but require linear time to check the existence of an edge. On the other hand, LAD requires quadratic memory to represent the data, but verifies the existence of edges in constant time We refer to the actual implementation of data structures of the algorithms in the released codes. Theoretically, efficient matrix implementations for sparse graphs could be used with moderate look-up time sacrifice. The search in adjacency lists could takes logarithmic time on the number of edges if a binary search were used on ordered lists of edges. Moreover, each algorithm uses several data structures besides the data structures to store the graphs. Therefore the resulting plots do not show the quadratic memory increases of an algorithm compared to another.

RI and VF2 do not use initial domains (which are compatibility maps among pattern and target vertices) or make use of variable domains. For this reason, FocusSearch, RI-Ds, RI-DsPm, and LAD check label similarity between vertices or edges once. On the other hand, RI and VF2 compare labels even if they have already done so in some previous step.

Since LAD, FocusSearch, RI-Ds and RI-DsPm, run a preprocessing phase to filter out the variable domains before the matching phase begins, they can potentially generate a smaller search space.

The extensive reduction operations of LAD prune the search space well at the price of a greater computational cost. FocusSearch applies cheaper reduction operations decreasing the running time but generating a larger search space. RI-Ds and RI-DsPm do not apply inference, therefore only the initial domains are reduced. RI and its two versions apply very light pruning rules and, therefore, they may generate a larger search space. In fact, the aim of our approach is to maintain a balance between the size of the generated search space and the time needed to visit it.

Summarizing the results (see Additional File 1 for detailed plots) we observed that

• RI always outperforms VF2.

• RI outperforms all other algorithms in sparse target graphs such as *AIDS*, *PDBSv1*, *PDBSv2*.

• RI is comparable with LAD and FocusSearch on small dense pattern graphs *PDBSv3 *with dense patterns, and with semidense small-medium patterns.

• RI outperforms LAD but not FocusSearch on small dense pattern graphs *PDBSv3 *with large semidense or sparse patterns.

Morever:

• We suggest to use RI-Ds on medium or large dense targets (such as *Graemlin *and *PPI *datasets). Here, RI-Ds outperforms (or comparable in total times with FocusSearch on *PPI *) all algorithms across different numbers of labels, pattern dimensions, and densities.

• We do not suggest the use of RI-DsPm or any costly inference or pruning rules. RI-DsPm does not improve performance compare to RI and RI-Ds. This behavior is supported by the analysis given in [29] (see in [29] Section 7.7). Since, our algorithm is independent of the used pruning rules, we also tried to run our algorithm with the rules from VFlib [3]. Experiments show that the rules helped to reduce the search space but their contribution were not significant and, in some cases, they increased the total time of the matching process. Therefore, we did not deploy those pruning rules.

These considerations validate the main idea in RI: a powerful pattern vertex ordering, i.e. strongly dependent only on the pattern graph topology, together with light constraint verification, is more efficient than a local or global inference procedure.

## Conclusions

Subgraph isomorphism is an important functionality of biochemical tools. This paper has made two intellectual and two pragmatic contributions to solving this inherently difficult problem. First, it proposes a new algorithm that outperforms existing algorithms in many though not all settings. Second it offers a perspective into when to choose which algorithm. Third, it provides implementations of the various leading algorithms. Fourth, it compares for the first time all most recent and popular subgraph isomorphism algorithms on biochemical data. In future work, we will apply these algorithms and similar analyses to approximate subgraph isomorphism search.

## Method

A measure of goodness for a subgraph isomorphism algorithm is its ability to reduce the search space. Our approach is based on the generally applicable observation that the order in which vertices of the pattern are matched is crucial to speeding up the pruning process. So, our algorithm starts by ordering the vertices of the pattern graph independently of any target graph and maintains the same variable ordering for all the branches of the search space. So, our search strategy is static and target independent.

### The static search strategy in RI

The search space tree has a dummy root. Each node represents a possible match between some vertex *u *of the pattern *G *and some vertex *u' *of the target graph *G'*. Let *M *: *V *→ *V' *be a mapping and *p_t _*= ((*u*_0_, *M*(*u*_0_)), (*u*_1_, *M*(*u*_1_)), ..., (*u_n_*, *M*(*u_n_*))) be a path of the state tree starting from the root. If *n <*|*V *| then *p_t _*is a partial match between *G *and *G'*. When *n *= |*V *|, *p_t _*is a full solution. Because our search strategy is static, each such full match maps the same order of vertices from the pattern graph to some sequence of vertices of the target graph.

Before the subgraph isomorphism process starts, RI orders the vertices of the pattern graph to maximize the chance that a partial path will be pruned away. This means that the ordering seeks to introduce as many edge constraints as possible and as early as possible in the ordering. Constraints are deduced only from the pattern graph and not from the target graph.

Recall that we have defined graphs as directed, that is (*u*, *v*) ∈ *E *does not imply that (*v*, *u*) is also in *E*. Thus, given a vertex *u *we could distinguish among edges going out from *u*, such as (*u*, *v*), from edges going into *u*, such as (*v*, *u*). Our search strategy considers the neighbors of a vertex *u *to be all edges touching *u*, without regard to directionality. So, here we denote the edge by 〈*u*, *v*〉.

Given the pattern graph *G*(*V*, *E*), let *n *= |*V*|. The aim is to define a suitable sequence of vertices *μ*=(*u*_0_,*u*_1_, ...,*u*_n_) of *V *. Specifically, at each step *i*, the vertex *u_i _*∈ *V *chosen is the one that maximizes the size of the set *B_i _*= {〈*u_i_*, *u_j_*〉 ∈ *E *: *u_j _*∈ *μ*, 0 *< j *≤ *i *≤ *n*}. *B_i _*represents the set of edges in the pattern graph connecting *u_i _*with vertices in *μ*. By making *B_i _*as large as possible, the algorithm imposes the most constraints on corresponding subgraphs of a potential target graph. That is, in the subgraph isomorphism process, RI first will be matched to nodes that are highly connected (i.e., a large number of constraints to verify) with nodes already matched.

**Figure 2:**
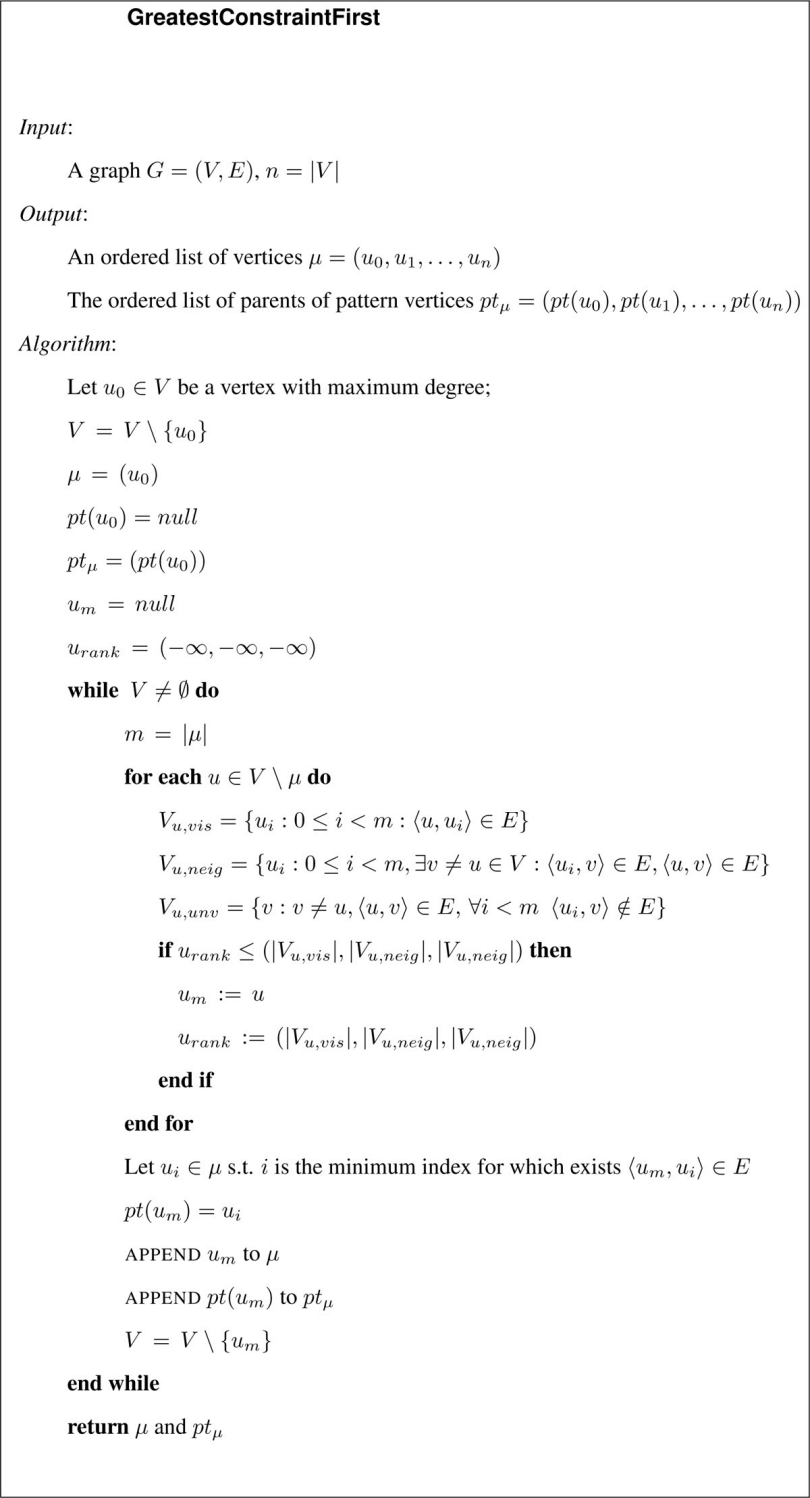
**GreatestConstraintFirst algorithm**. The algorithm generates an order on the pattern vertices, sequence *μ*, that during the subgraph isomorphism process will maximize the number of topological constraints as early as possible in the matching process.

We use a greedy algorithm called GreatestConstraintFirst to find a good sequence of vertices *μ*. GreatestConstraintFirst visits the pattern graph based on a scoring function. It starts from a vertex *u*_0 _in the pattern graph that has the maximum number of neighbors among any vertex in the pattern graph. The algorithm iteratively proceeds until all vertices in the pattern graph are inserted in *μ*. For each vertex *v *not yet in the sequence (*u*_0_, *u*_1_, ..., *u*_*m*−1_) we maintain the concept of vertex parent, that is the vertex *u_i _*in the sequence with the smallest index *i *such that 〈*u_i_*, *v*〉 ∈ *E*. Figure 2 shows the pseudocode of the algorithm.

The scores are assigned in the following way. Let *m *be the next step of a visit in the pattern graph and let *μ *(*u*_0_, *u*_1_, ..., *u*_*m*−1_) the visited vertices so far. Let *u_m _*be the next candidate vertex to be inserted in *μ*.

We can ascribe a score to *u_m _*using the following three sets.

1 *V_m,vis _*= {*u_i _*: 0 ≤ *i < m *: 〈*u_m_*, *u_i_*〉 ∈ *E*}, the set of vertices in *μ *that are neighbors of *u_m_*.

2 *V_m,neig _*= {*u_i _*: 0 ≤ *i < m*, ∃*j > m *: (*u_i_*, *u_j _*) ∈ *E*, (*u_m_*, *u_j _*) ∈ *E*}, the set of vertices in *μ *each of which is a neighbor of at least one vertex outside *μ *that is connected to *u_m_*.

3 *V_m,unv _*= {*u_j _*: *j > m*, (*u_m_*, *u_j_*) ∈ *E*, ∀*i < m *(*u_i_*, *u_j_*) ∉ *E*}, the set of vertices that are not in *μ*, not even neighbors of vertices in *μ *but are neighbors of *u_m_*.

The score of candidate *u_m _*is a lexicographic score based on |*V_m,vis_*| as the high order quantity, followed by |*V_m,neig_*| and finally |*V_m,unv_*|. Thus, suppose *u_a _*and *u_b _*are both candidates. The score of *u_a _*is greater than the score of *u_b _*if either (i) |*V_a,vis_*| *>*|*V_b,vis_*| or (ii) |*V_a,vis_*| = |*V_b,vis_*| and |*V_a,neig_*| *>*|*V_b,neig_*| or (iii) |*V_a,vis_*| = |*V_b,vis_*| and |*V_a,neig_*| = |*V_b,neig_*| and |*V_a,unv_*| *>*|*V_b,unv_*|. If two vertices tie for the highest score, then we choose one arbitrarily and keep track of the other.

Note that, if we are working with directed graphs then our algorithm increases the score more when both underlying directed edges are present than if only one of the pair exists.

**Figure 3:**
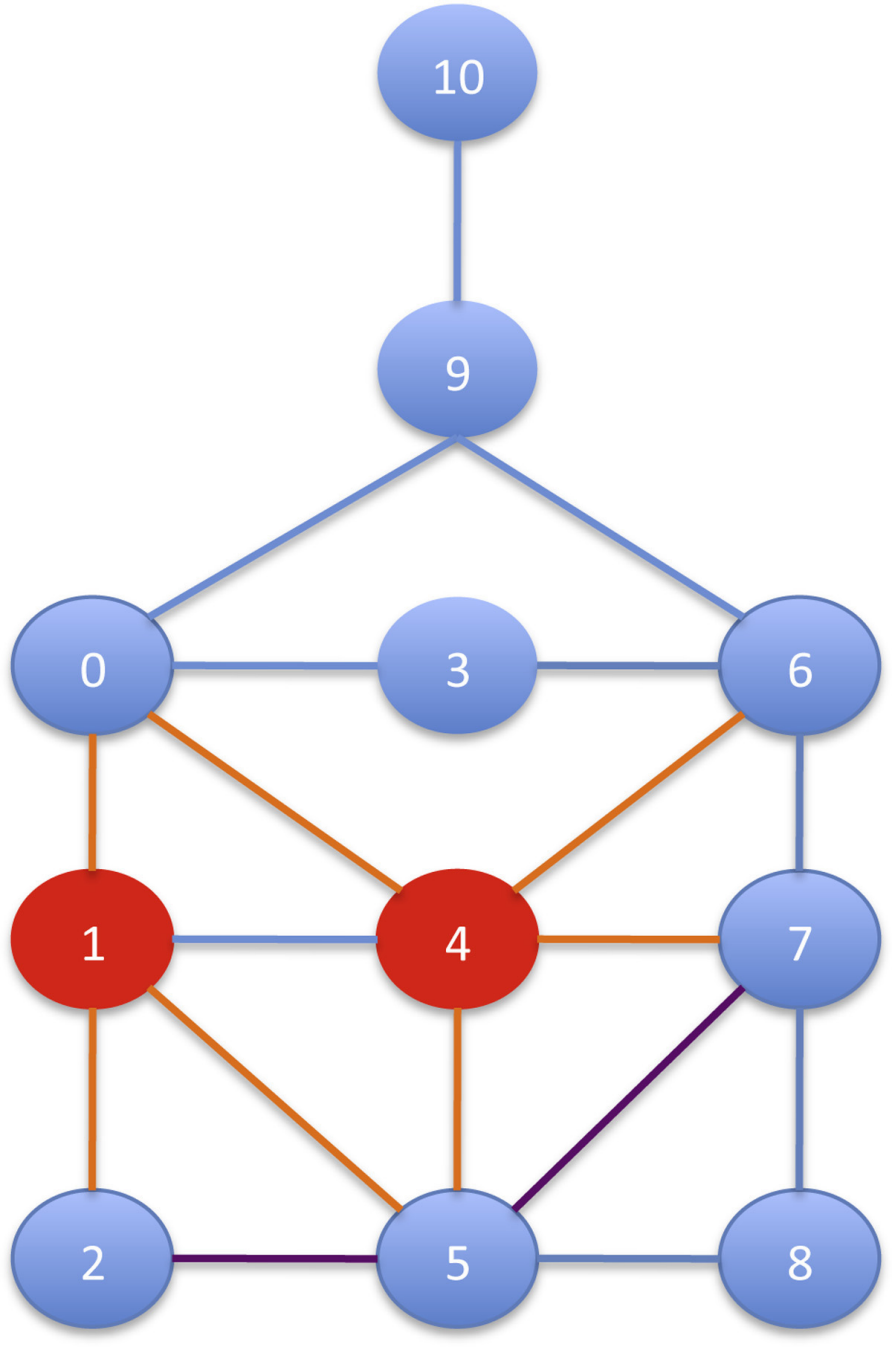
**Search strategy in RI**. The sequence of pattern vertices produced by the static search strategy of RI. The first vertex inserted in *μ *is 4, since it has the greatest number of edges. Then, suppose that *μ *= {4, 1}, the candidates to be inserted are vertices 0,2,6, 5 and 7. The next vertex that will be inserted in *μ *is 5, because vertex 5 has a greater number of edges pointing neighbors of vertices in *μ *(i.e. point to 2 and 7) than vertex 0. Even though the node 5 has fewer edges pointing to all remaining vertices (i.e. consider the edge 〈5, 8〉 for 5, and for 0 the edges 〈0, 3〉 and 〈0, 9〉), this case has less weight in in defined score.

Figure 3 reports an example of the RI search strategy. The first vertex inserted in *μ *is 4, since it has the highest number of edges. Then, suppose that *μ *= {4, 1}, the candidates to be inserted are vertices 0,2,6,5 and 7. The next vertex that will be inserted in *μ *is 5. The reason is that, even though it has the same number of edges pointing to vertices in *μ *as vertex 0, the vertex 5 has a higher number of edges pointing to neighbors of vertices in *μ *(i.e. point to 2 and 7). Even though the node 5 has fewer edges pointing to all remaining vertices (i.e. consider the edge 〈5, 8〉 for 5, and for 0 the edges 〈0, 3〉 and 〈0, 9〉), this case has less weight in the defined score.

Finally, we point out that our search strategy does not favor (i.e. put in the ordering first) more dense parts of the pattern graph nor the most central vertex according centrality measures such as between centralities and so on. In fact, in the example in Figure 3 the most central vertices 6 and 0 are not at the beginning of the ordering.

### Reduce search space procedure

Each node in the search space tree represents a mapping from a vertex in the pattern graph to a vertex in the target graph. All paths from the root downward in the tree correspond to the order of vertices *μ *in the pattern graph generated by GreatestConstraintFirst. The vertices from the pattern graph must be compared to every vertex in the target graph to see whether it satisfies the subgraph isomorphism conditions. Each step consists of choosing, at each level *i*, the candidate vertices in the target graph, $u_{i}^{\prime }=M\left(u_{i}\right)$ to match *u_i_*, among the neighbors of the matched vertices of the parents of *u_i_*. Parents of the pattern graph are constructed in GreatestConstraintFirst. The following isomorphism conditions prune away un-feasible paths.

1. Neither *u_i _*nor *M*(*u_i_*) is already matched in the current path.

2. The matched vertices are compatible, i.e., *lab*(*u_i_*) ≡ *lab*(*M*(*u_i_*)).

3. The number of edges connected to *M*(*u_i_*) in *V' *is greater than or equal to the number of edges connected to *u_i _*in *V*. That is |{(*v'*, *M*(*u_i_*)) ∈ *E'*}| ≥ |{(*w*, *u_i_*) ∈ *E*}| and |{(*M *(*u_i_*),*v'*) ∈ *E' *}| ≥ |{(*u_i_*, *w*) ∈ *E*}|. In the case of undirected graphs the it verifies that |〈*M*(*u_i_*), *v'*〉 ∈ *E'*}| ≥ | 〈*u_i_*, *w*〉 ∈ *E*}|.

4. The constraints deriving from the topology of the pattern graph up to this point in the path are met, ∀*u_i_*, *u_j _*∈ *V *where 0 ≤ *j *≤ *i *(*u_i_*, *u_j_*) ∈ *E *⇒ (*M*(*u_i_*), *M*(*u_j_*)) ∈ *E' *If edges are labeled, then *β *is defined, we would also verify the compatibility of the edge labels.

**Figure 4:**
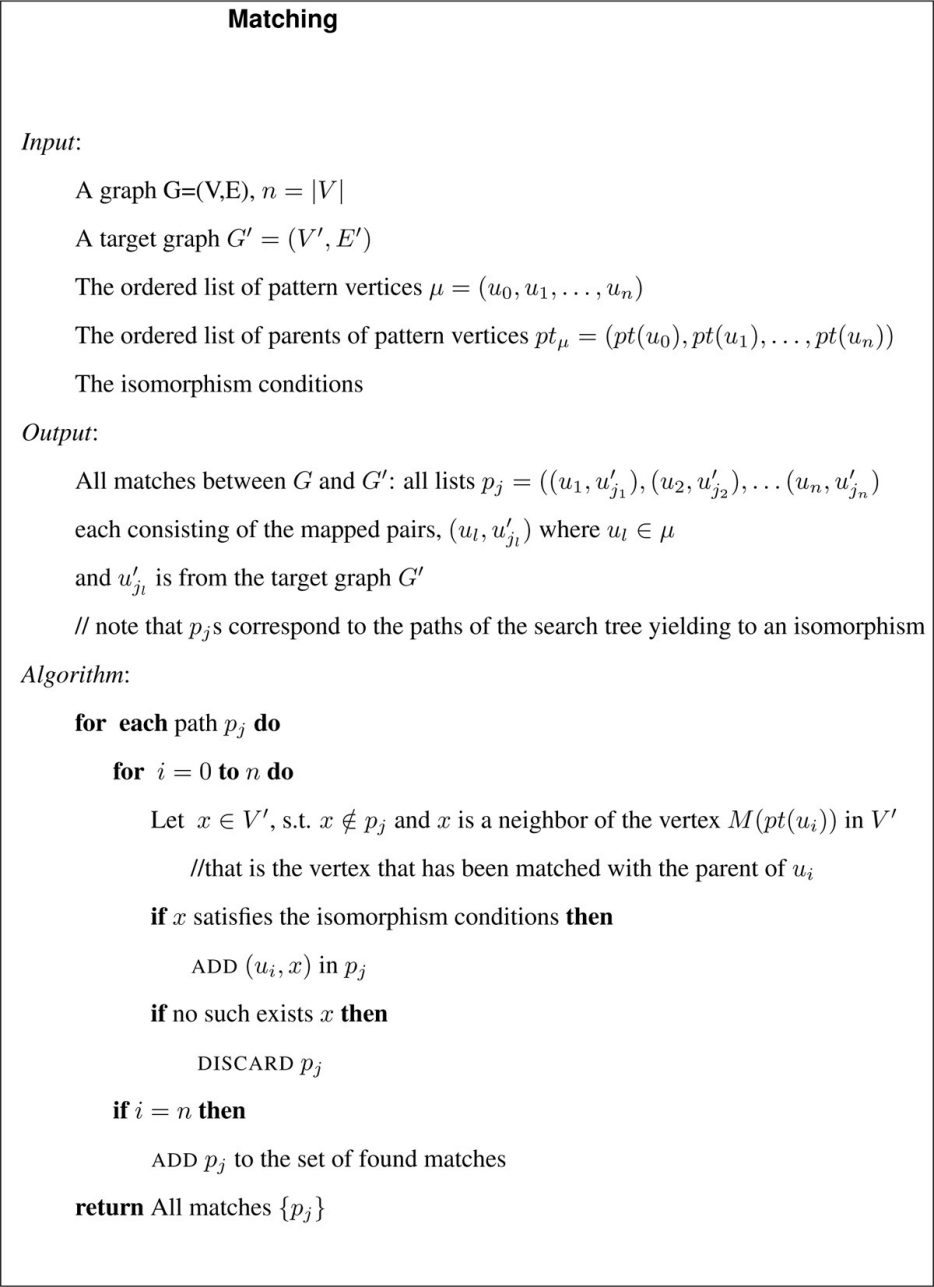
**Matching algorithm**. Matching algorithm in RI

The isomorphism conditions are tested in the above. Condition *i *is verified only if condition *i *− 1 does not fail. Conditions 1, 2, and 4 ensure the isomorphism, whereas the third one is a filtering test that often obviates the need for the substantial work needed to verify condition 4. The above matching procedure, called Matching, is illustrated in Figure 4.

### Algorithmic aspects of RI vs existing popular algorithms

RI is the first to suggest a static and target independent search strategy. It reduces the search space by applying only the subgraph isomorphism conditions rather than costly filtering rules as in [3] or inference procedures as in [2,29,30]. It's not a priori clear that this will be better. Inference or pruning rules that look at the target graph and that consider the actually partial solutions can reduce the space search more, but may be more expensive.

VFlib [3] and LAD [30] define a dynamic search strategy that looks at the target graph; FocusSearch [29] defines a partly dynamic search strategy, almost independent from the target graph. FocusSearch first looks at the target graph to run a domain reduction by filtering them using vertex invariants based on labels and topology. This is used to select the first vertex of the variable ordering. Then it constructs the rest of the sequence of vertices by applying topology constraints only on the pattern graph. FocusSearch has less pruning power than LAD, but the overhead is also less.

Besides the fact that RI is static, the rules are different too. For example, (i) in contrast to VFlib rules, RI does not distinguish among edges going out or into a vertex, (ii) FocusSearch can handle only integer label edges, and (iii) LAD cannot perform induced subgraph isomorphism and cannot deal with edge labels. Table 2 summarizes the main differences among RI, VFlib and FocusSearch.

**Table 2:** Comparison of subgraph isomorphism algorithms.

	Search Stratergy	Reduce Search Space	Preprocessing Data	x Data Structure
FocusSearch [29]	Static Semi-target dependent	Local domain reduction	Yes	List

Lad [30]	Dynamic Target dependent	Domain reduction until convergence	Yes	Matrix

VFlib [3]	Dynamic Target dependent	Two-Look-Head pruning rules	No	List

RI	Static Target independent	Fast and light pruning rules	No	List

Review of some algorithmic aspects of the most recent subgraph isomorphism algorithms.

## Competing interests

The authors declare that they have no competing interests.

## Authors' contributions

VB and RG conceived and designed the algorithm. VB implemented the algorithm. RG, AP, DS and AF contributed to analysis aspects. RG, AP, DS, and AF supervised the project. All authors wrote and approved the manuscript.

## Declarations

This article is published as part of a supplement. The publication costs for this article were funded by PO grant - FESR 2007-2013 Linea di intervento 4.1.1.2, CUP G23F11000840004.

## Supplementary Material

Additional file 1 **Results and applications**. It completes the Results and Discussion Section with the detailed description of all used datasets and obtained results. It reports an application of subgraph isomorphism on protein complexes searching on GO annotated *PPI *dataset. Click here for file

Additional file 2 **Average matching and total time performances on *AIDS *and *PDBS *datasets**. For *PDBSv3*, tests are grouped with respect to pattern densities. For each algorithm, the average of its result values (expressed in sec) is reported together with the standard deviation (see Additional File 1 for more detailed results). The best algorithm is highlighted in bold. Click here for file

Additional file 3 **Average space and memory requirements on *AIDS *and *PDBS *datasets**. For *PDBSv3*, tests are grouped with respect to pattern densities. For each algorithm, the average of its result values (expressed as the number of space's nodes and kilobytes) is reported together with the standard deviation (see Additional File 1 for more detailed results). The best algorithm is highlighted in bold. Click here for file

Additional file 4 **Average matching and total time performances on *Sansone et al *dataset**. Tests are grouped with respect to target topologies. For each algorithm, the average of its result values (expressed in sec) is reported together with the standard deviation (see Additional File 1 for more detailed results). The best algorithm is highlighted in bold. Click here for file

Additional file 5 **Average space and memory requirements on *Sansone et al *dataset**. Tests are grouped with respect to target topologies (see Additional File 1 for more detailed results). For each algorithm, the average of its result values (expressed as the number of space's nodes and kilobytes) is reported together with the standard deviation. The best algorithm is highlighted in bold. Click here for file

Additional file 6 **Average matching and total time performances on *Graemlin *dataset**. Tests are grouped with respect to the number of labels as shown in the plots (see Additional File 1 for more detailed results). For each algorithm, the average of its result values (expressed in sec) is reported together with the standard deviation. The best algorithm is highlighted in bold. Click here for file

Additional file 7 **Average space and memory requirements on *Graemlin *dataset**. Tests are grouped with respect to the number of labels as shown in the plots (see Additional File 1 for more detailed results). For each algorithm, the average of its result values (expressed as the number of space's nodes and kilobytes) is reported together with the standard deviation. The best algorithm is highlighted in bold. Click here for file

Additional file 8 **Average matching and total time performances on *PPI *dataset**. Tests are grouped with respect to the number of labels and label distributions as shown in the plots (see Additional File 1 for more detailed results). For each algorithm, the average of its result values is reported together with the standard deviation. The best algorithm is highlighted in bold. Click here for file

Additional file 9 **Average space and memory requirements on *PPI *dataset**. For each dataset, tests are grouped with respect to respect to the number of labels and label distributions as shown in the plots (see Additional File 1 for more detailed results). For each algorithm, the average of its result values (expressed as the number of space's nodes and kilobytes) is reported together with the standard deviation. The best algorithm is highlighted in bold. Click here for file

## References

[B1] McKay B Practical graph isomorphism Congressus Numerantium 1981 30 45 87

[B2] Ullmann J An algorithm for Subgraph Isomorphism Journal of the Association for Computing Machinery 1976 23 31 42 10.1145/321921.321925

[B3] Cordella L Foggia P Sansone C Vento M A (Sub)Graph Isomorphism Algorithm for Matching Large Graphs IEEE Transactions on Pattern Analysis and Machine Intelligence 2004 26 10 1367 1372 10.1109/TPAMI.2004.75 15641723

[B4] Milo R Shen-Orr S Itzkovitz S Kashtan N Chklovskii D Alon U Network motifs: simple building blocks of complex networks Science 2002 298 5594 824 827 10.1126/science.298.5594.824 12399590

[B5] Kashani Z Ahrabian H Elahi E Nowzari-Dalini A Ansari E Asadi S Mohammadi Schreiber FS Masoudi-Nejad A Kavosh: a new algorithm for finding network motifs BMC Bioinformatics 2009 10 318 10.1186/1471-2105-10-318 19799800 PMC2765973

[B6] Wernicke S Rasche F FANMOD: a tool for fast network motif detection Bioinformatics 2006 22 1152 1153 10.1093/bioinformatics/btl038 16455747

[B7] Ferro A Giugno R Pigola G Pulvirenti A Skripin D Bader GD Shasha D NetMatch: a Cytoscape plugin for searching biological networks Bioinformatics 2007 23 7 910 912 10.1093/bioinformatics/btm032 17277332

[B8] Daylight Chemical Information Systems http://www.daylight.com/

[B9] Frowns http://frowns.sourceforge.net/

[B10] Lemons NW Hu B Hlavacek W Hierarchical graphs for rule-based modeling of biochemical system BMC Bioinformatics 2011 12 45 21288338 10.1186/1471-2105-12-45 PMC3152790

[B11] Kucukural A Szilagyi A Sezerman U Zhang Y Chemoinformatics: Advances in Chemoinformatics and Computational Methods, Protein Homology Analysis for Function Prediction with Parallel Sub-Graph Isomorphism 2009 IGI global

[B12] Garey M Johnson D Computers and Intractability: A Guide to the Theory of NP-Completeness 1979 Freeman and Company

[B13] Giugno R Shasha D GraphGrep: A Fast and Universal Method for Querying Graphs Proceeding of the International Conference in Pattern recognition (ICPR), ICPR ’02 2002 112 115

[B14] Yan X Yu P Han J Graph indexing: a frequent structure-based approach Proceedings of the ACM SIGMOD international conference on Management of data 2004 SIGMOD '04 335 346

[B15] Cheng J Ke Y Ng W Lu A Fg-index: towards verification-free query processing on graph databases Proceedings of the 2007 ACM SIGMOD international conference on Management of data 2007 SIGMOD '07 857 872

[B16] Di Natale RD Ferro A Giugno R Mongiovì M Pulvirenti A Shasha D SING: Subgraph search In Non-homogeneous Graphs BMC Bioinformatics 2010 11 96 10.1186/1471-2105-11-96 20170516 PMC2850364

[B17] Bonnici V Ferro A Giugno R Pulvirenti A D S Enhancing Graph Database Indexing by Suffix Tree Structure Proceedings of the 5th IAPR international conference on Pattern recognition in bioinformatics 2010 195 203

[B18] Zhang S Hu M Yang J TreePi: A Novel Graph Indexing Method Proceedings of IEEE 23rd International Conference on Data Engineering 2007 181 192

[B19] He H Singh AK Closure-Tree: An Index Structure for Graph Queries ICDE '06: Proceedings of the 22nd International Conference on Data Engineering 2006 3822915836

[B20] Zou L Chen L Yu JX Lu Y A novel spectral coding in a large graph database Proceedings of the 11th international conference on Extending database technology: Advances in database technology, EDBT ’08 2008 181 192

[B21] Inokuchi A Washio T Motoda H An Apriori-Based Algorithm for Mining Frequent Substructures from Graph Data Proceedings of the 4th European Conference on Principles of Data Mining and Knowledge Discovery 2000 PKDD '00 13 23

[B22] Kuramochi M Karypis G Frequent Subgraph Discovery Proceedings of the 2001 IEEE International Conference on Data Mining 2001 ICDM '01 313 320

[B23] Yan X Han J gSpan: Graph-Based Substructure Pattern Mining Proceedings of the 2002 IEEE International Conference on Data Mining 2002 ICDM '02 721

[B24] Zhu F Qu Q Lo D Yan X Han J Yu PS Mining Top-K Large Structural Patterns in a Massive Network PVLDB 2011 4 11 807 818

[B25] Fan W Li J Ma S Tang N Wu Y Wu Y Graph pattern matching: from intractable to polynomial time Proc. VLDB Endow 2010 3 1-2 264 275

[B26] Gallagher B Matching structure and semantics: A survey on graph-based pattern matching AAAI FS 2006 6 45 53

[B27] Cheng J Yu JX Ding B Yu PS Wang H Fast Graph Pattern Matching Proceedings of the 2008 IEEE 24th International Conference on Data Engineering 2008 ICDE '08 913 922

[B28] Fan W Li J Luo J Tan Z Wang X Wu Y Incremental graph pattern matching Proceedings of the 2011 ACM SIGMOD International Conference on Management of data 2011 SIGMOD '11 925 936

[B29] Ullmann JR Bit-vector algorithms for binary constraint satisfaction and subgraph isomorphism J Exp Algorithmics 2011 15 1.6 1.1 1.6 1.64

[B30] Solnon C AllDifferent-based filtering for subgraph isomorphism Artificial Intelligence 2010 174 850 864 10.1016/j.artint.2010.05.002

[B31] Tarjan R Yannakakis M Simple linear-time algorithms to test chordality of graphs,test acyclicity of hypergraphs, and selectively reduce acyclic hypergraphs SIAM J Comput 1984 13 566 579 10.1137/0213035

[B32] Shier D Some aspects of perfect elimination orderings in chordal graphs Discr Appl Math 1984 325 331

[B33] Bacchus F van Run P Dynamic variable reordering in CSPs CP '95 Proceedings of the First International Conference on Principles and Practice of Constraint Programming 1995 258 27523444276

[B34] Lecoutre C Constraint Networks: Techniques and Algorithms 2009 ISTE/Wiley

[B35] Messmer BT Bunke H Subgraph Isomorphism Detection in Polynominal Time on Preprocessed Model Graphs Proceedings of Asian Conference on Computer Vision 1995 373 382

[B36] Akinniyi F Wong A Stacey D A new algorithm for graph monomorphism based on the projections of the product graph Trans Systems, Man and Cybernetics 1986 740 751

[B37] Cortadella L Valiente G A relational view of subgraph isomorphism Proceedings of fifth international seminar on relational methods in computer science 2000 45 54

[B38] Barrow H Burstall RM Subgraph Isomorphism, Matching Relational Structures and Maximal Cliques Information Processing Letters 1976 4 83 84 10.1016/0020-0190(76)90049-1

[B39] Henderson TC Discrete Relaxation Techniques 1990 Oxford University Press

[B40] Horaud R Skordas T Stereo Correspondence Through Feature Grouping and Maximal Cliques IEEE Transactions on Pattern Analysis and Machine Intelligence 1989 11 11 1168 1180 10.1109/34.42855

[B41] Levi G A note on the derivation of maximal common subgraphs of two directed or undirected graphs Journal of Calcols 9 1972 341 354

[B42] Myaeng SH Lopez-Lopez A Conceptual graph matching: a flexible algorithm and experiments Journal of Experimental Theoretical Artificial Intelligence 1992 4 107 126 10.1080/09528139208953741

[B43] Nilsson N Principles of artificial intelligence 1980 Palo Alto CA: Tioga

[B44] Sanfeliu A Fu K A Distace Measure between Attributed Relational Graphs for Pattern Recognition IEEE Transactions on Systems Man and Cybernetics 1983 13 3 353 362

[B45] Wong A You M Entropy and Distance of Random Graphs with Application to Structural Pattern Recognition IEEE Transactions Pattern Analysis and Machine Intelligence 1985 7 5 599 609 10.1109/tpami.1985.4767707 21869297

[B46] Lipets V Vanetik N Gudes E Subsea: an efficient heuristic algorithm for subgraph isomorphism Data Min Knowl Disc 2009 19 320 350 10.1007/s10618-009-0132-7

[B47] National Cancer Institute http://www.nci.nih.gov/

[B48] Huehne R Suehnel J The Jena Library of Biological Macromolecules Nature-precedings 2009

[B49] Protein Data Bank http://www.rcsb.org/pdb/

[B50] Vehlow C Stehr H Winkelmann M Duarte JM Petzold L Dinse J Lappe M CMView: Interactive contact map visualization and analysis Bioinformatics 2011 27 11 1573 1577 10.1093/bioinformatics/btr163 21471016

[B51] Flannick J Novak A Srinivasan B McAdams H Batzoglou S Graemlin: general and robust alignment of multiple large interaction networks Genome research 2006 16 9 1169 10.1101/gr.5235706 16899655 PMC1557769

[B52] Dickinson P Bunke H Dadej A Kraetzl M On graphs with unique node labels Lecture Notes in Computer Science 2003 2726

[B53] Szklarczyk D Franceschini A Kuhn M Simonovic M Roth A Minguez P Doerks T Stark M Muller J Bork P Jensen L von Mering C The STRING database in 2011: functional interaction networks of proteins, globally integrated and scored Nucleic Acids Res 2011 39 D561 D568 10.1093/nar/gkq973 21045058 PMC3013807

[B54] Foggia P Sansone C Vento M A Database of Graphs for Isomorphism and Sub-Graph Isomorphism Benchmarking Proceedings of the 3rd IAPR TC-15 Workshop on Graph-based Representations in Pattern Recognition 2001 176 187

