# Supplementary material for: A subgraph isomorphism algorithm and its application to biochemical data
Source: BMC Bioinformatics. 2013 Apr 22;14(Suppl 7):S13. doi: 10.1186/1471-2105-14-S7-S13 (PMC3633016; doi:10.1186/1471-2105-14-S7-S13)
Supplement: Additional file 1 — Results and applications. It completes the Results and Discussion Section with the detailed description of all used datasets and obtained results. It reports an application of subgraph isomorphism on protein complexes searching on GO annotated PPI dataset. [file 1471-2105-14-S7-S13-S1.pdf]

Results and Application of RI

Results

Tests are performed on real datasets (*AIDS*, *PDBSv1*, *PDBSv2*, *PDBSv3*, *Graemlin* and *PPI*) and the synthetic dataset distributed by Sansone et al. All datasets are labeled using the real information (such as atoms, protein domains, protein names). Since in *Graemlin* and *PPI* each vertex has a unique label, we performed tests using the networks with their unique labels and with randomly assigned labels varying the number from 32, 64, 128, 256, 512, 1024, to 2048. Label assignment follows uniform and normal distributions in different tests. Patterns are extracted from the target graphs varying the density and the dimension. For each dataset we report in Table 1 and/or in plots the number of matches. All algorithms are deterministic and correct therefore they report the same number of matches. We refer to the main paper for a complete description of the datasets and the related patterns.

We point out that all algorithms end. However, tests are run with a timeout of 3 minutes to the total execution time of the algorithms. We chose this timer since it reflects in proportion the results reported in [7] (where test are run with a timeout of 1 hour). For each dataset we report how many subgraph isomorphism runs each algorithm completes before the timeout. When an algorithm times out, we exclude the related running times from the means of all algorithms.

The total time needed by an algorithm includes the time to read graphs from files, build data structures, run preprocessing operations, run the real matching phase and so on. Therefore, we distinguish between the total time and the matching time. The matching time for RI and VF2[1] regards the matching process; instead for LAD[7] and FocusSearch[8] it also includes the preprocessing time. Notice that, the preprocessing steps are the first parts of the matching processes. The space size is the number of visited nodes of the hypothetical search space tree and the memory size is the amount of kilobytes required to store all data structures. Each algorithm uses several data structures besides those to store the graphs.

In Figures 1, 2, 3, 4, 5, 6, 7, 8, 9 and 10 we give a summery view of the results in each dataset. Next, for each dataset, we report all plots separately.

Algorithm timeout RI shows overall the best behavior. Results for VF2 on *PPI* datasets are not reported since they often time out. FocusSearch times out on dense datasets with small queries (*Graemlin* and *PPI* datasets). LAD sometimes times out on large graphs (*PPI* dataset).

Number of matches The number of matches is related to the density of the patterns and to the number of labels. For example, in *PDBSv1* (see Table 1), the number of subgraph isomorphisms for patterns of sizes 64 and 128 is larger than for smaller pattern sizes. This may due to the fact those patterns match parts of the backbones of the proteins and parts of their surfaces. Protein surfaces are rich in atoms of the same type (such as hydrogen). Thus, the number of possible subgraph isomorphisms increases. However, larger patterns may have fewer matches (see the case of size 256 for sparse patterns (see Table 1, *PDBSv3*). As can be expected, dense patterns have fewer subgraph isomorphisms than semidense patterns (see Table 1, *PDBSv3*, Figure 5) and patterns on target graphs with 512 labels have more matches

than patterns on target graphs with 2048 labels (see Figure 7).

Search space and memory requirement Since LAD and FocusSearch run a preprocessing phase to filter out the variable domains before the matching phase begins, they can potentially generate a smaller search space as in Figures 1, 2, 3,5, 8, 9. However, in *Graemlin* and *PPI* with 32 labels datasets and in the synthetic dataset (see Figures 4, 8 and 10), LAD has a larger search space than all other algorithms. On the other hand, FocusSearch maintains low search spaces in all datasets. This is due to the fact that the datasets have a small number of different labels, in the spirit of [8], Section 7.7 ”Molecular graph retrieval experiments”.

In otherdatasets (*AIDS*, *PDBSv1*, and *PDBSv2* in Figures 1, 2 and 3), the extensive reduction operations of LAD prune the search space very well and better than FocusSearch at the price of a greater computational cost. FocusSearch applies low-priced reduction operations decreasing the running time but generating a larger search space.

The fact that topology-based filtering is more effective than label-based reduction procedures is consistent with our strategy because *the static order of RI is based on the pattern topology*.

By looking at all Search Space and Matches plots we notice that the search space curves are, for all algorithms and in particular for FocusSearch, dependent on the number of matches in almost all datasets (see for example the plots for *PDBSv3* or *PPI* datasets).

Concerning the memory requirement, all plots show that RI consumes a little memory compared to FocusSearch and LAD, which need extra memory to store compatible maps, domains, and to perform reduction operations. RI-DsPm increases the consumption of memory compared to RI-Ds whose consumption is similar to RI . RI-DsPm and RI do not perform inference or pruning, so the search space is larger than for FocusSearch and LAD. FocusSearch, by using also bit vectors, requires less memory than LAD. In Figure 3 the memory required by LAD in dense targets is comparable with all other algorithms and again increases in the sparse targets (*PDBSv2*) which may be due to the fact that LAD uses adjacent matrices. Matrices are an advantage in dense graphs. For such graphs therefore, LAD uses less memory than other algorithms.

Total time and Matching time The total and matching time plots in all datasets, obtained by varying label distributions, pattern dimensions, pattern densities in sparse and dense targets, show that RI and RI-Ds outperforms all other algorithms. Note that, even though RI has to explore larger search spaces than LAD and FocusSearch, it explores the search space faster since it does not perform reductions. Summarizing we make the following observations.

- RI always outperforms VF2.
- RI outperforms all other algorithms in sparse target graphs such as *AIDS*, *PDBSv1*, *PDBSv2* and synthetic data.
- RI is comparable with LAD and FocusSearch on small dense pattern graphs, *PDBSv3*, with dense patterns, and with semidense small-medium patterns.
- RI outperforms LAD but not FocusSearch on small dense pattern graphs *PDBSv3* with large semidense or sparse patterns.

- RI-Ds outperforms LAD on medium dense *Graemlin* datasets using a small number of labels, for example, 32 labels. It is comparable with LAD and FocusSearch in all target graphs of the datasets.
- In total time, RI-Ds is comparable with FocusSearch and outperforms all others across the number of labels, pattern dimensions, and densities on large dense *PPI* datasets. In particular it outperforms LAD. In matching time RI-Ds also outperforms FocusSearch.
- RI outperforms all algorithms on all graph types (bounded, meshes and random) of the synthetic datasets.
- Plots show that by adding any other even light reduction steps on RI does not improve performances (see the behavior of RI-DsPm).
- RI has a low memory requirement and times out less than other algorithms.

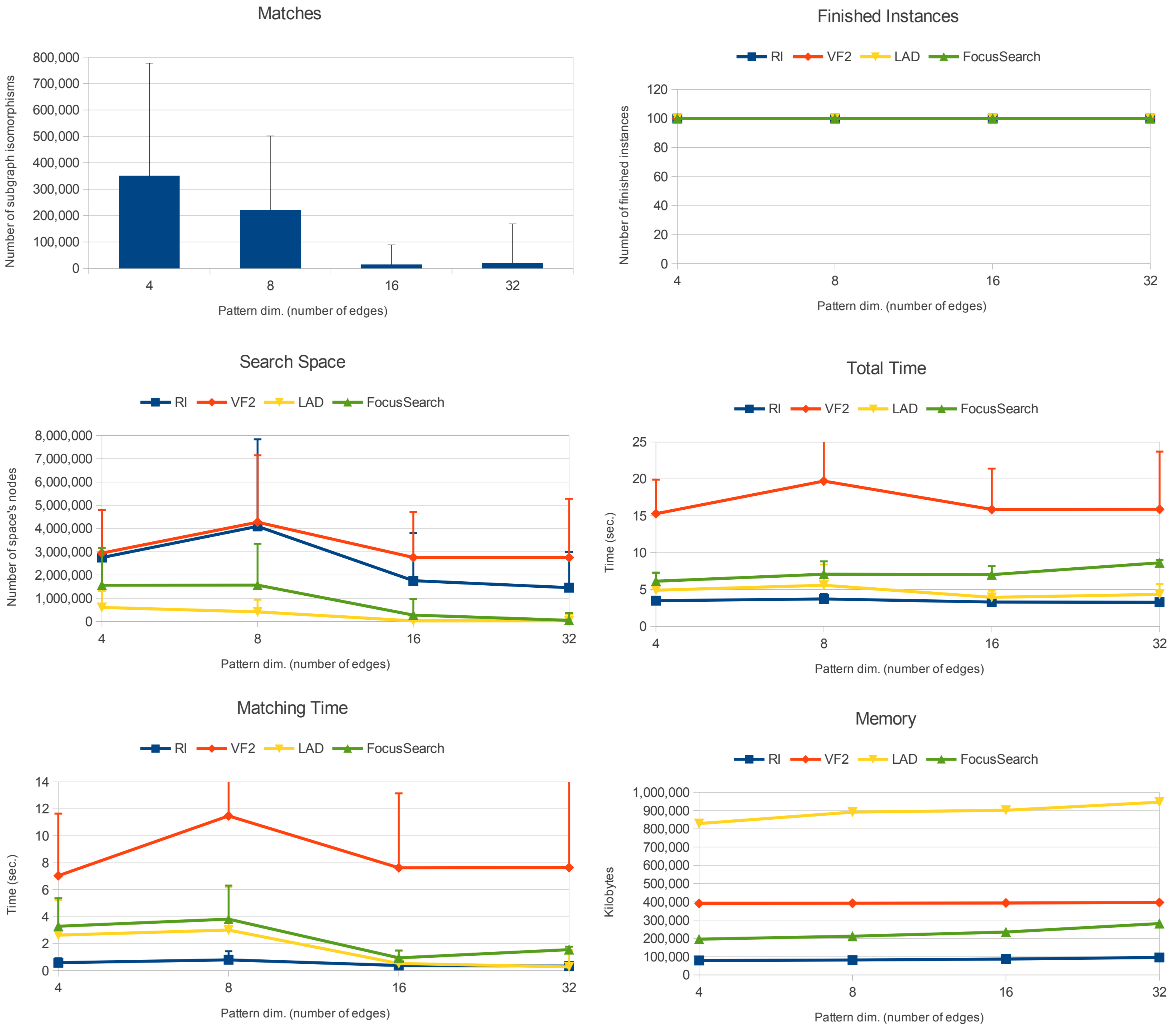

Figure 1: Results on *AIDS* dataset.

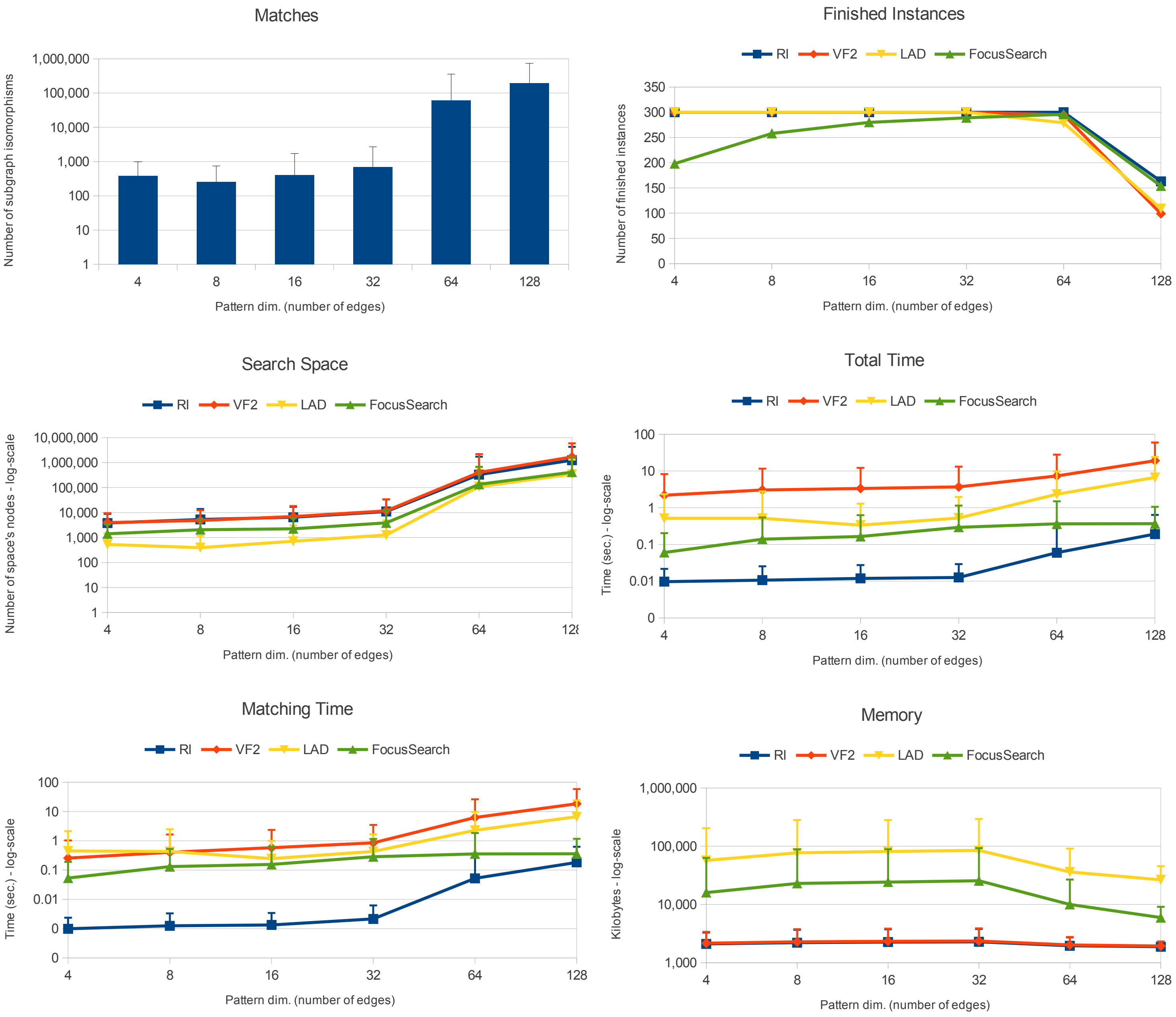

Figure 2: Results on *PDBSv1* dataset.

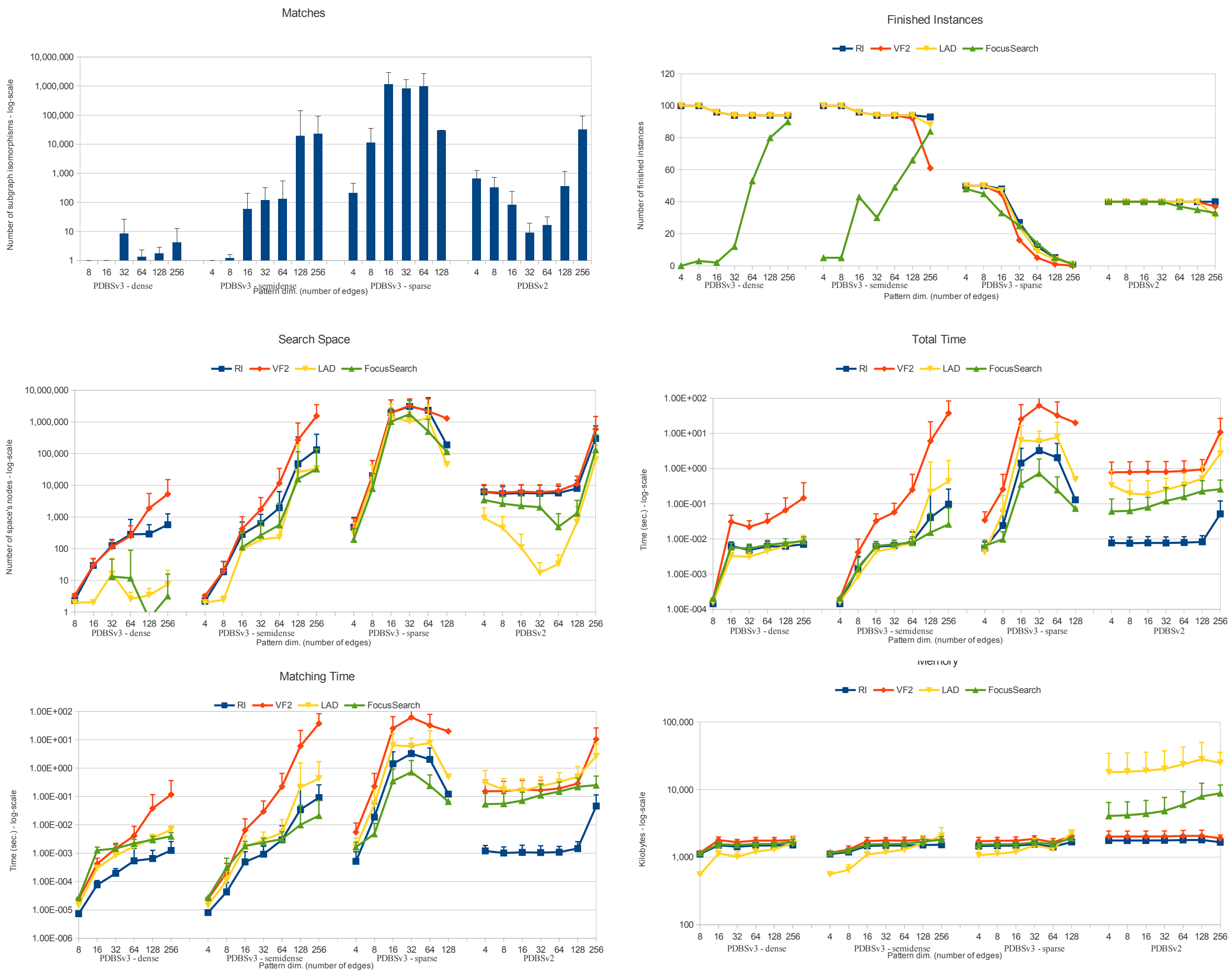

Figure 3: Results on *PDBSv2* and *PDBSv3* datasets.

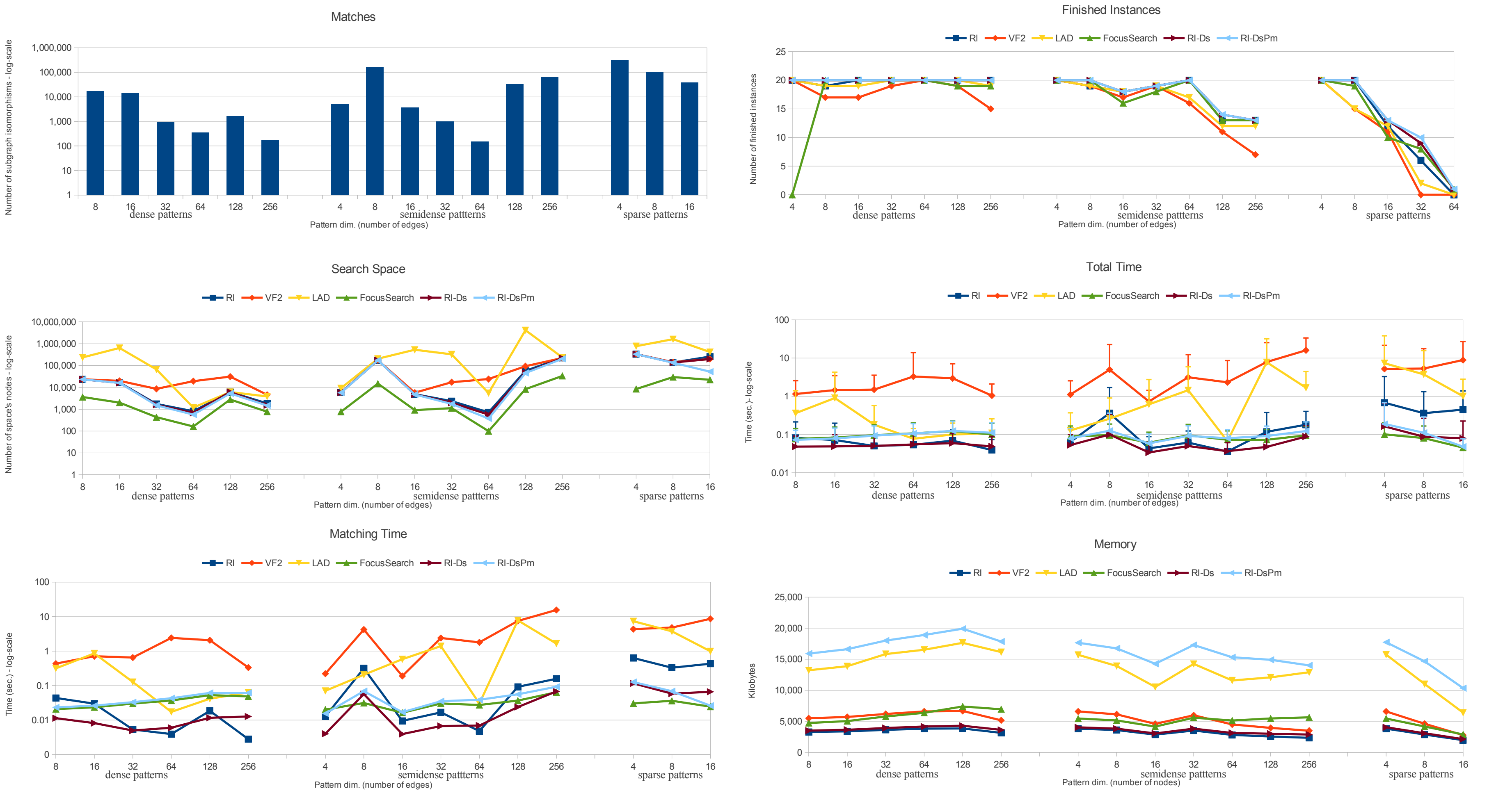

Figure 4: Results on *Graemlin* dataset. Graphs are labeled using 32 labels uniformly distributed.

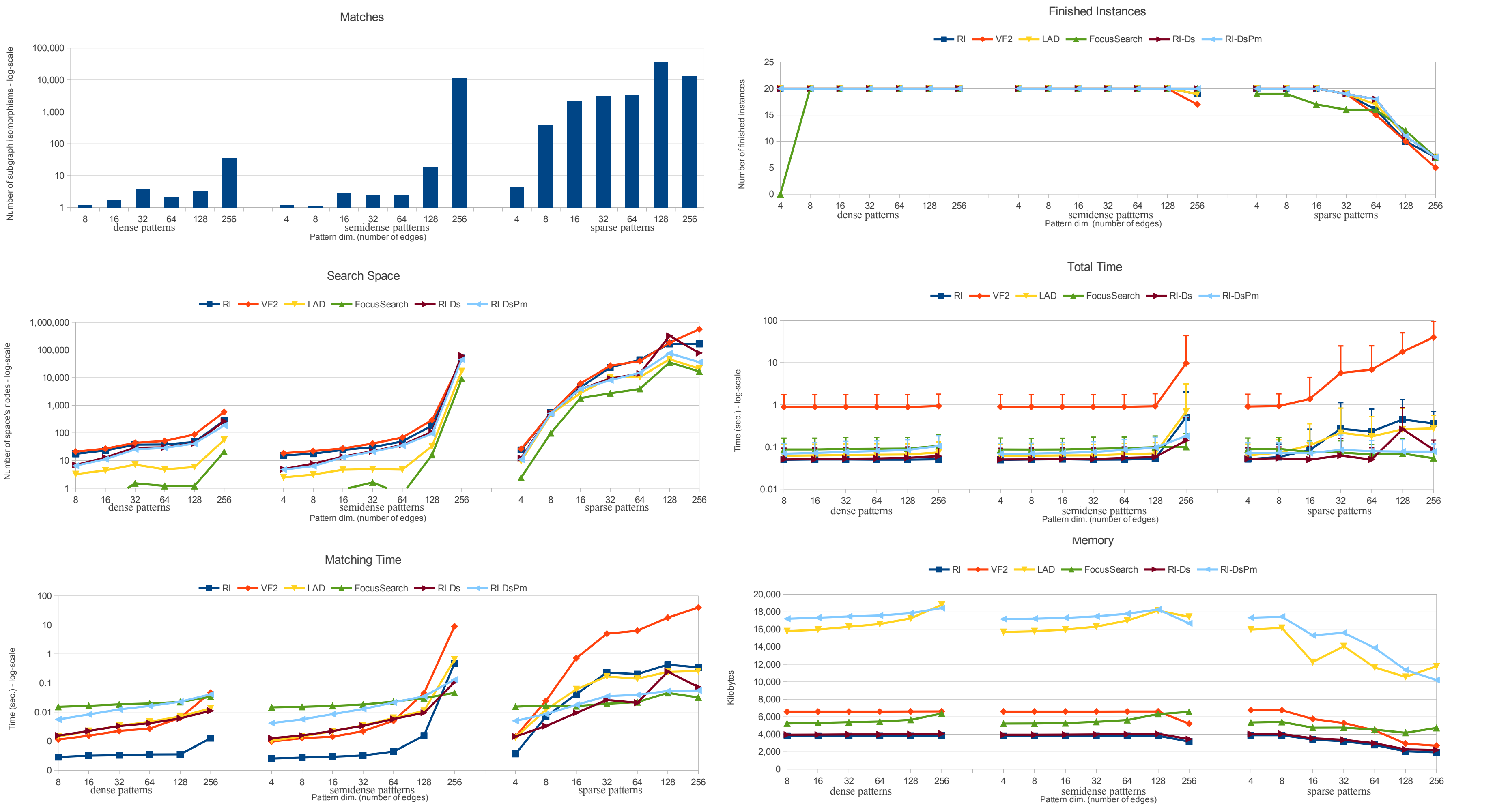

Figure 5: Results on *Graemlin* dataset. Graphs are labeled using 256 labels uniformly distributed.

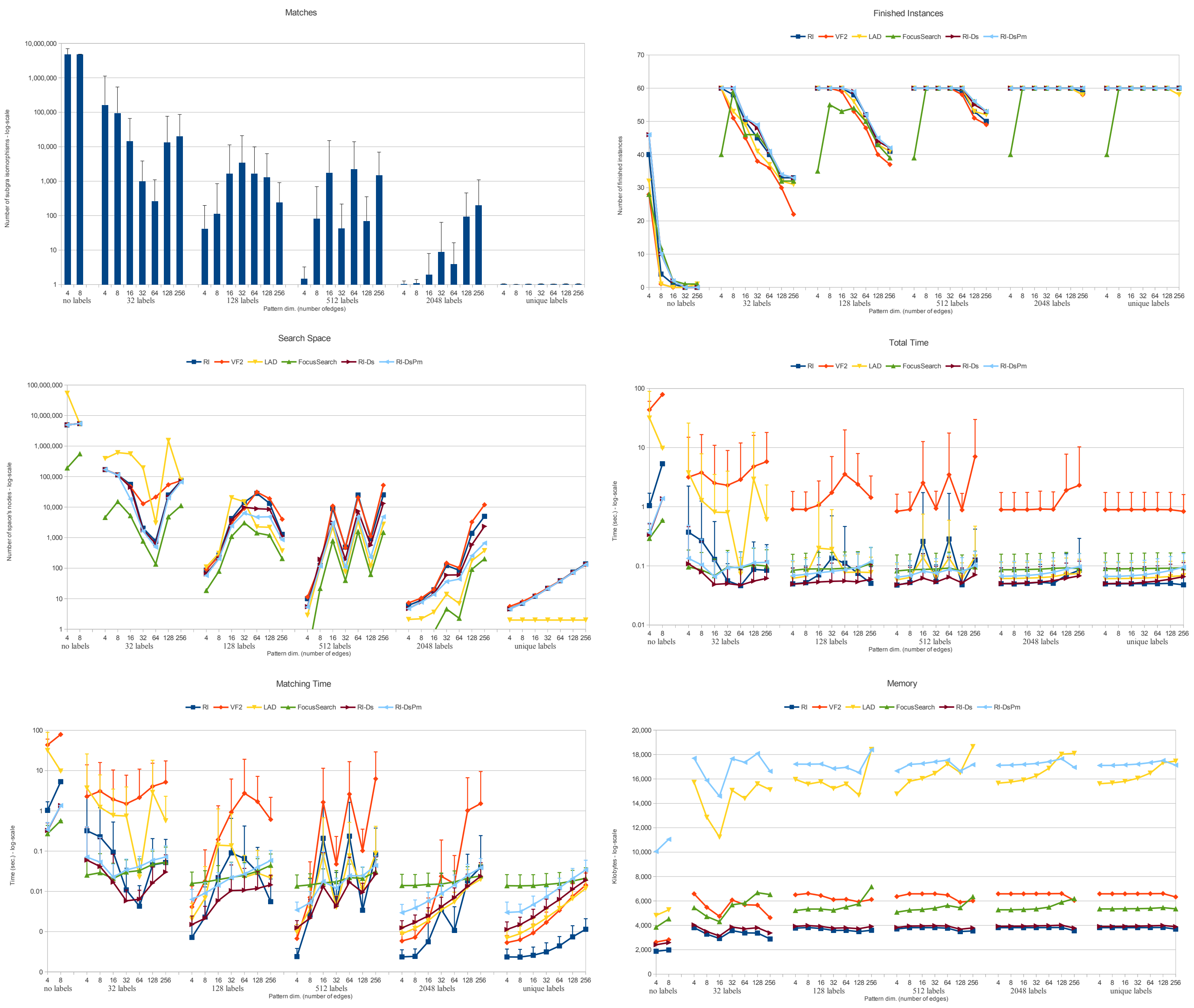

Figure 6: Results on *Graemlin* dataset varying the number of labels.

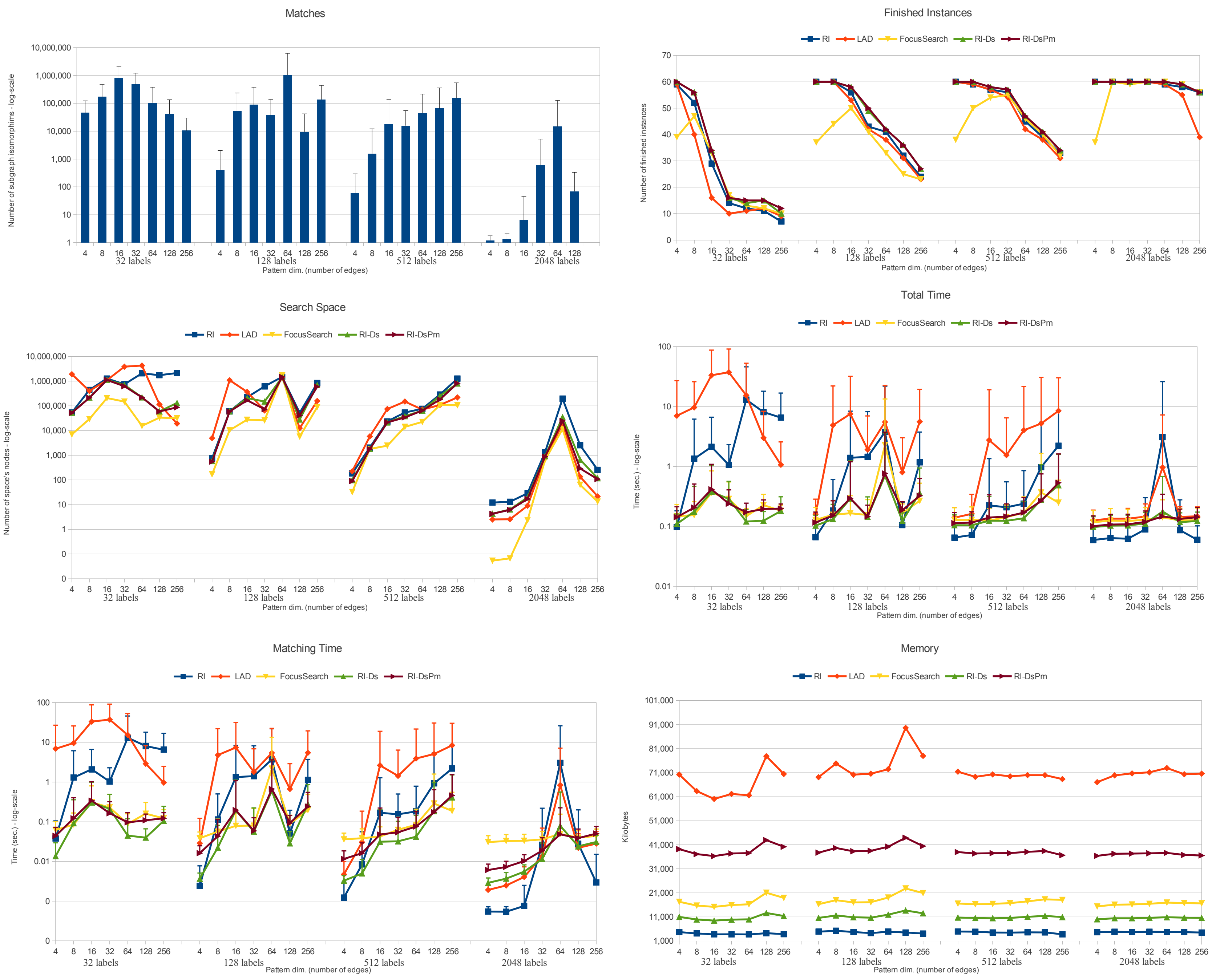

Figure 7: Results on *PPI* dataset. Graphs are labeled using a normal distribution

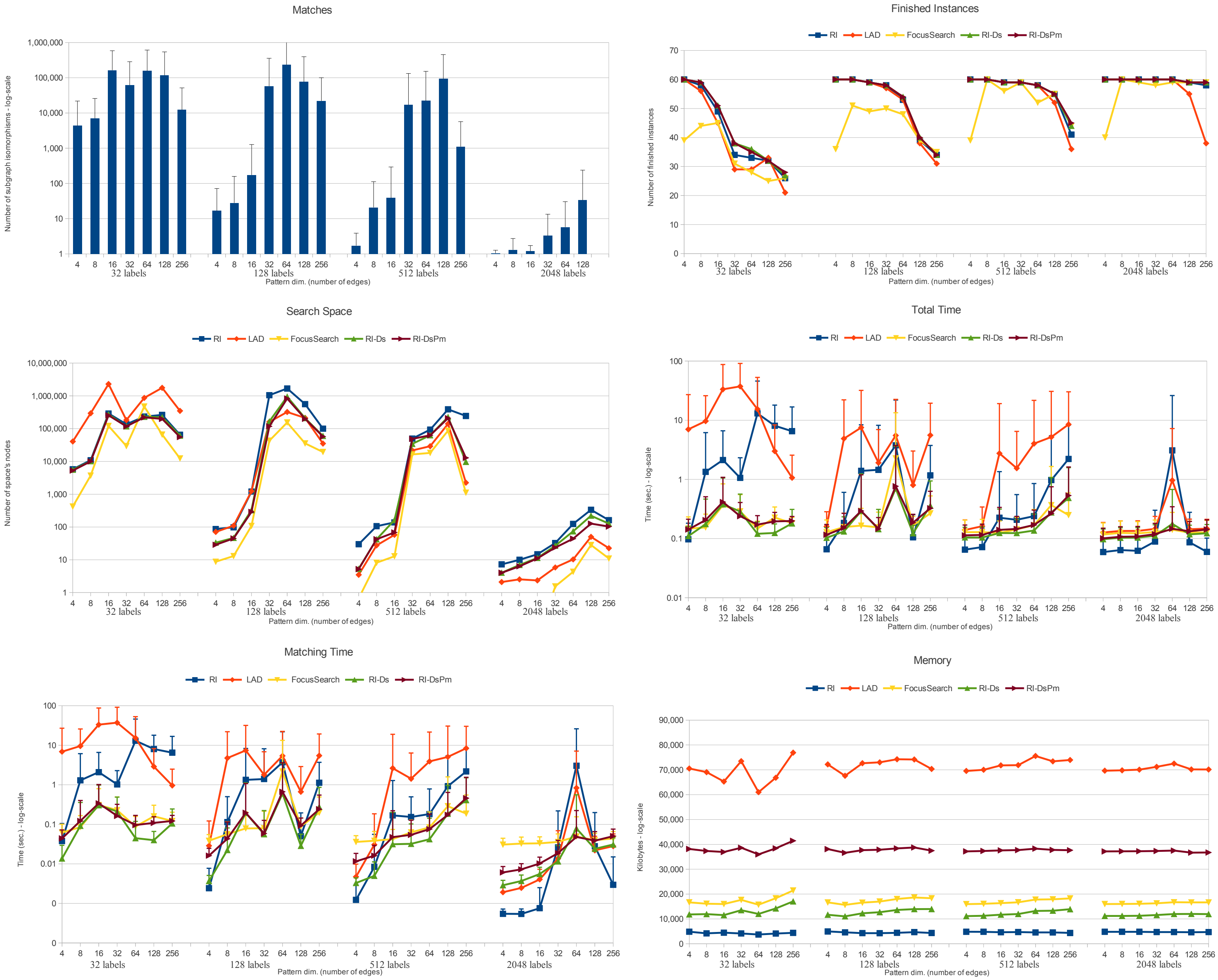

Figure 8: Results on *PPI* dataset. Graphs are labeled using a uniform distribution.

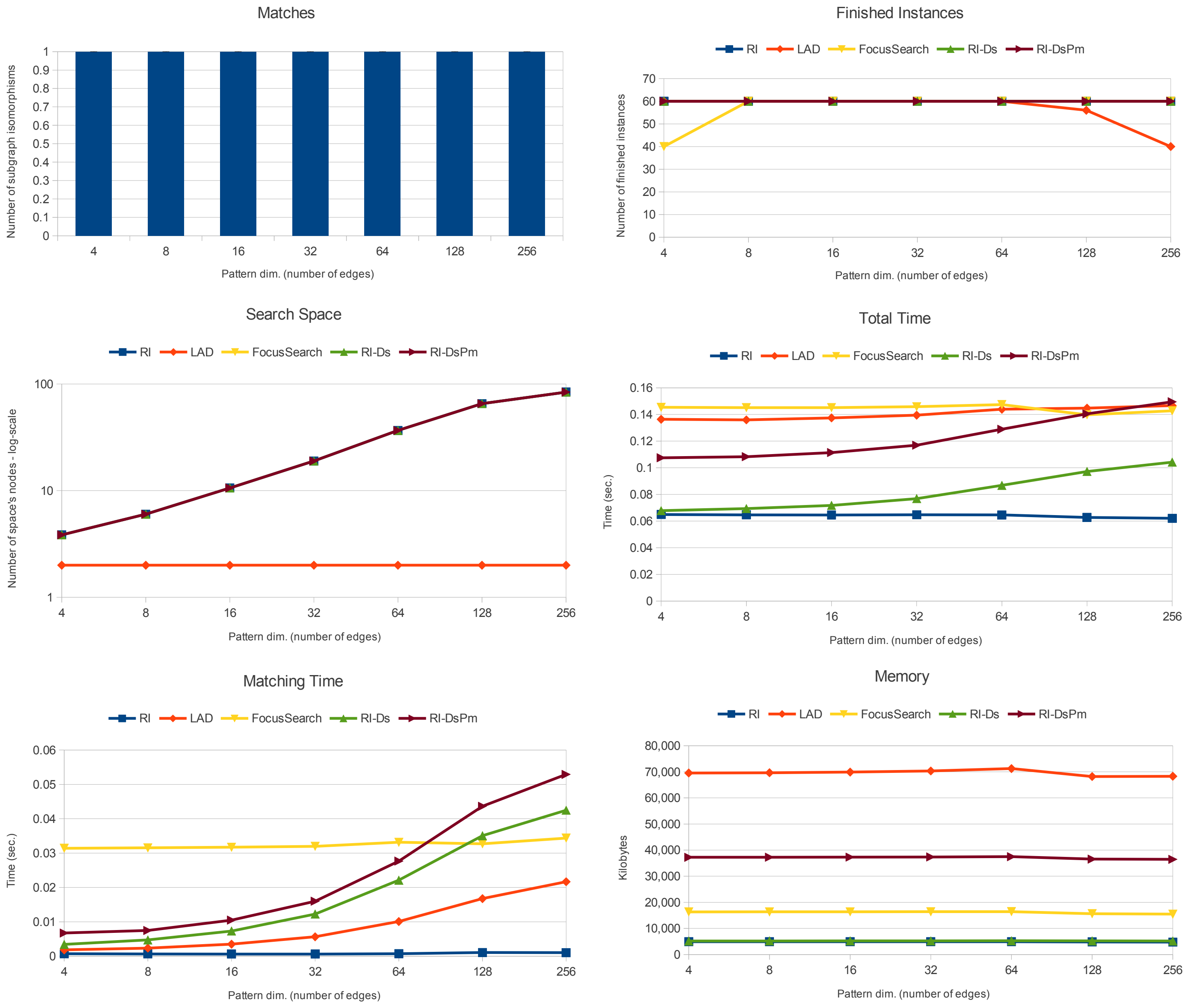

Figure 9: Results on *PPI* dataset. Graphs are labeled using the original protein names. Each vertex has a unique label.

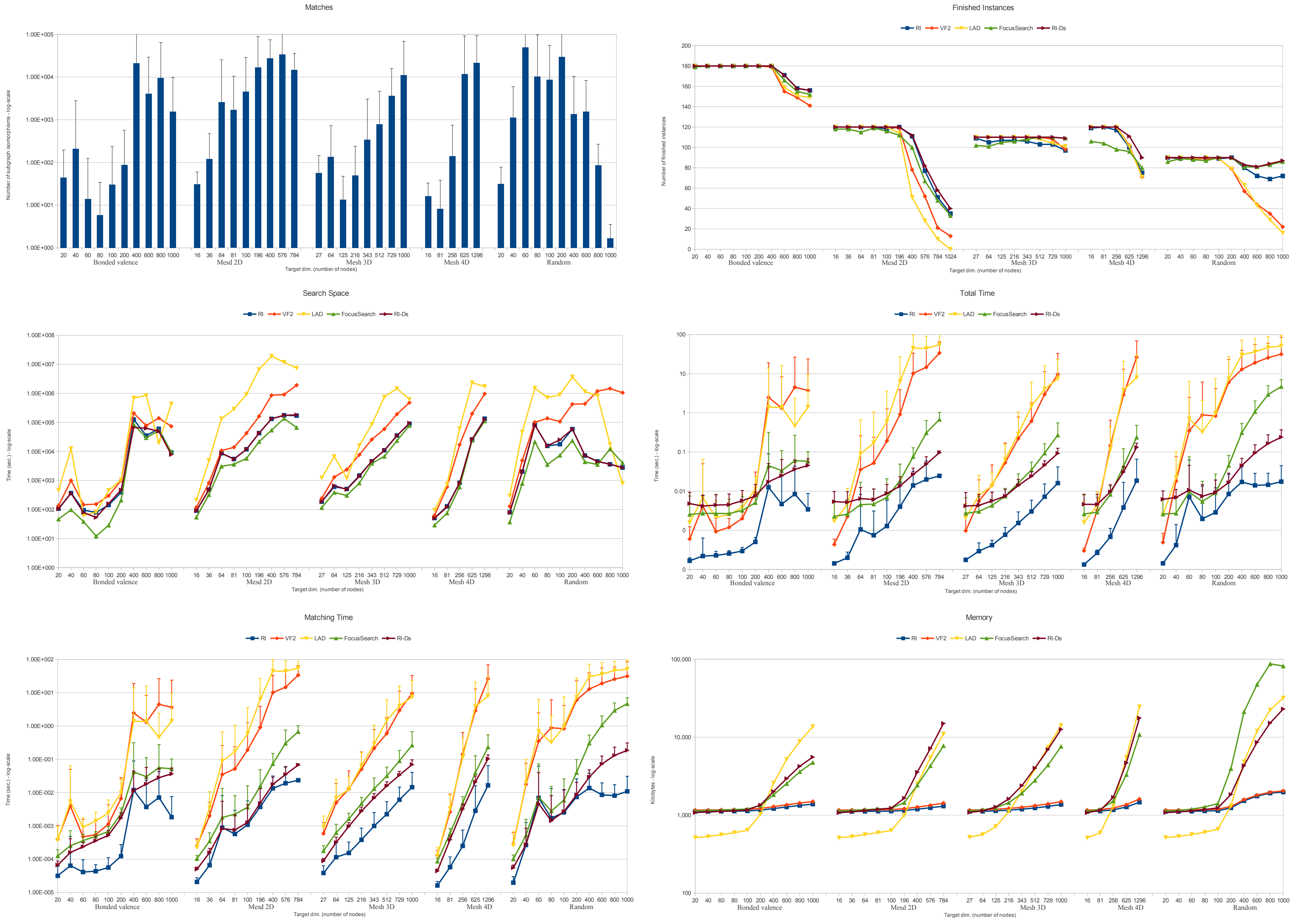

Figure 10: Results on synthetic datasets distributed by Sansone et al.

AIDS dataset

Figure 11 shows the total search space size in terms of the number of visited tree nodes. As expected, LAD generates the smallest search space, FocusSearch outperforms RI due to the preprocessing and reduction operations and RI outperforms VF2. Figures 12 , 13, and 14 show the means of the memory requirements, matching and total running time. RI outperforms all the other algorithms.

PDBSv1 dataset

Figure 15 shows the number of times the algorithms end before the timeout. Figure 16 shows that LAD and FocusSearch have a better search space pruning while in Figure 17 RI and VF2 show the low memory requirements. RI outperforms all other systems in matching and total time (see Figures 18 and 19).

PDBSv2 and *PDBSv3* datasets

Figure 20 shows the number of times the algorithms end before the timeout. For the *PDBSv2* dataset, Figure 21 shows the space size. CSP based methods (LAD and FocusSearch) have an effective size reduction on increasing the density of the patterns. Figure 22 shows the memory requirements. RI and VF2 maintain a low profile. By contrast, since LAD and FocusSearch store compatibility maps and variables domains, their memory requirements increase with the number of vertices of the target graphs. Furthermore, since LAD uses adjacency matrices, it gives better results when the number of edges per vertex increases. For each pattern vertex, LAD and FocusSearch must store a structure containing the domain of that variable. This results in a potential quadratic space requirement to store compatibility maps and domains. However, FocusSearch uses bit-vector representations for compatibility maps and domains, so it may maintain a more flat profile though still quadratic. Figures 23 and 24 show the matching and the total time. RI outperforms the other methods except for the some patterns matched into dense targets (i.e. Contact Map, *PDBSv3*). This behavior is due to the small number of constraints present in the patterns. RI and VF2 seem to explode as pattern sizes (number of edges) increase in sparse graphs (proteins backbones) but this is due to the absence of compatibility maps. As discussed before, VF2 and RI need linear time to check for edge existence versus the constant time needed when compatibility maps are used.

Graemlin dataset

Figures 25 and 26 show the number of matches and the number of times the algorithms end before the timeout, respectively. The number of matches decreases increasing the number of labels. Notice that, in Figure 27, FocusSearch has a smaller space requirement than LAD. This is due to the vertex labeling and the prematch process of FocusSearch which is based on neighborhood labels. However, LAD outperforms all other methods on targets graph with unique labels. Figure 28 shows the memory requirements. RI has the lowest memory requirement. Figures 29 and 30 show the matching and the total times, respectively. The total time of RI is comparable with all other systems besides VF2

using unique or 2048 labels, whereas on the same targets, RI outperforms all other systems. By decreasing the number of labels, RI-Ds shows the best performances both in matching and total time.

PPI dataset

We do not report the performance of VF2 on this dataset because it solved just a few instances. Figures 31 and 32 show the number of matches using a normal and uniform distribution, respectively. The number of matches decreases with increasing numbers of labels. Figures 33, 34 and 35 show the number of times the algorithms end before the timeout using unique label, normal, and uniform label distribution, respectively. RI results the more robust. Figures 36, 37 and 38 show the space size using unique label, normal, uniform label distribution, respectively. LAD has a very low space size using unique labels. FocusSearch has the best performances using normal and uniform distribution. Figures 39, 40 and 41 and 42, 43 and 44 show the matching and the total times using unique label, normal, uniform label distribution, respectively. RI shows very low matching and total time using unique labels. RI-Ds outperforms all other systems in the rest of the target garphs.

Synthetic dataset

Our synthetic dataset consists of the graphs distributed by Sansone et al. They are pairs of unlabeled graphs having sizes varying from 20 to 1000 vertices. We refer to their main paper for the dataset detail statistics. The dataset contains the following kinds of graphs:

- *Bounded Valence*: The number of edges per vertex varies from 3, 6, 9.
- *Mesh*: 2D, 3D and 4D, where 2,3,4 indicate the dimensionality of the meshes.
- *Random*: Edges are added according to a probability; edges are independent and the probability distribution is uniform.

Since the synthetic dataset is composed of a pair of (target and pattern) graphs, we do not need to generate patterns for it. Results are showed in Figures 45, 46, 47, 48, 49 and 50. RI outperforms FocusSearch and LAD on matching, memory and total time. This is due to the fact that those graphs are unlabeled, so the inference rules of FocusSearch and LAD have less power because they are partly (note that LAD and FocusSearch reduce better the space) based on labels.

Table 1 - Statistics of Biochemical Patterns Subgraph Isomorphisms.

All algorithms are exact and deterministic and find the same number of subgraph isomorphisms. Patterns are divided per size and/or per density. For each pattern type, the table reports the average number of subgraph isomorphisms (SI) and its related standard deviation (SD).

	Pattern Size	Avg Number of SI	SD of Avg Number of SI
AIDS	4	350120.86	427210.75
	8	220855.34	280818.33
	16	14703.55	73942.02
	32	20495.16	148230.81
PDBSv1	4	379.83	607.87
	8	257.74	483.74
	16	400.44	1307.04
	32	696.16	1998.14
	64	61484.71	295970.22
	128	193612.03	542854.14
PDBSv2	4	655.53	584.92
	8	326.18	390.59
	16	82.18	157.66
	32	9	10.17
	64	16.54	15.01
	128	361.37	793.5
PDBSv3			
Dense	8	1	0
	16	1	0
	32	8.5	17.72
	64	1.32	0.99
	128	1.71	1.14
	256	4.11	8.39
Semi-dense	8	1.2	0.4
	16	58.86	145.48
	32	119.97	198.94
	64	129.8	417.9
	128	18953.57	123154.79
	256	23128.58	67948.13
Sparse	8	208.5	466.96
	16	11104.38	20141.36
	32	1135928.3	1949694.37
	64	814667.64	3094065.45
	128	974924.8	2325178.8
	256	30120.02	189904

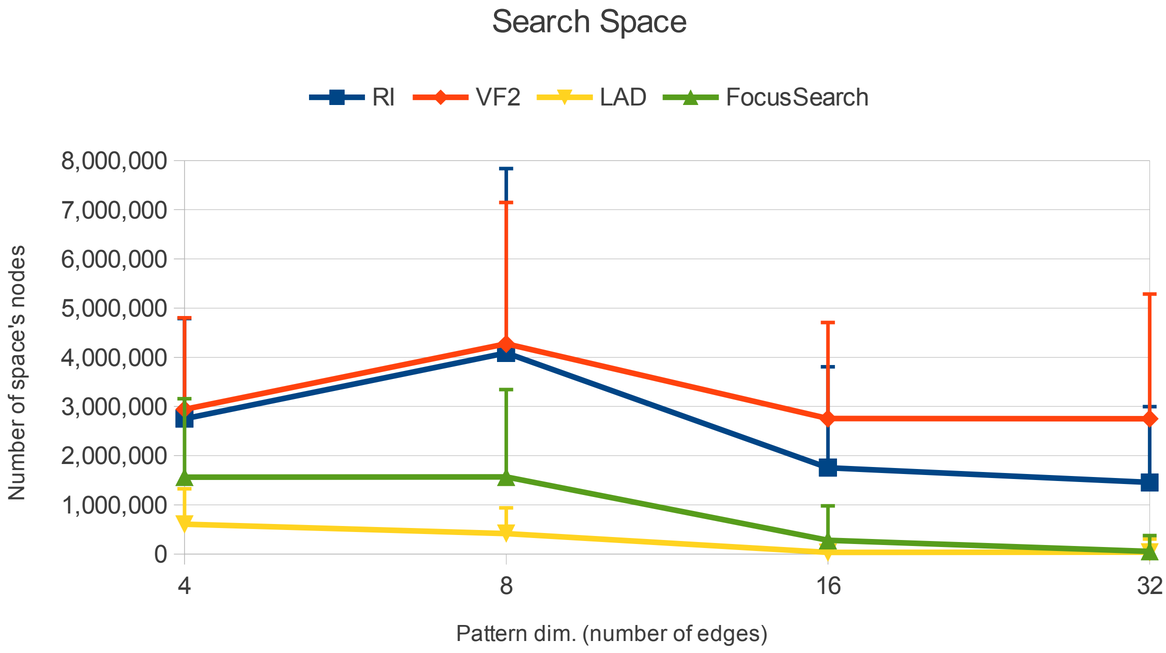

Figure 11: Averages of search space sizes on *AIDS* dataset for different patterns having 4, 8, 16 and 32 edges. The chart shows the number of visited nodes. Reduction based methods (LAD and FocusSearch) outperform RI and VF2. RI outperforms the VF2 heuristics. The efficiency of the search strategy of RI increases with the number of pattern edges.

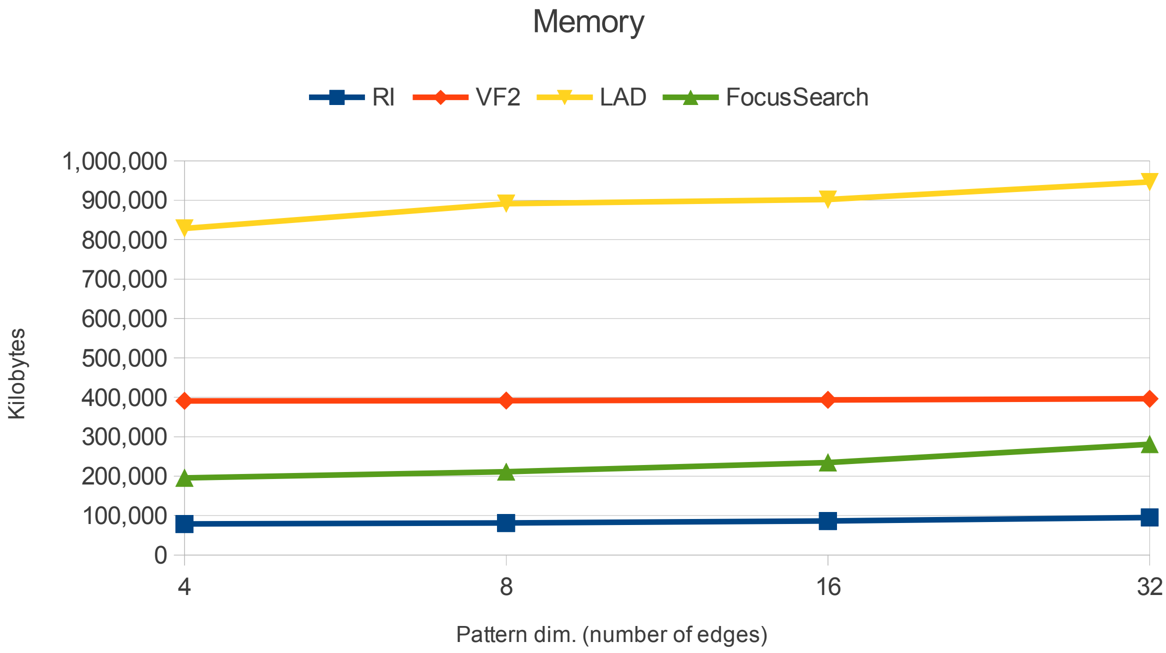

Figure 12: Averages of memory requirement on *AIDS* dataset for different patterns having 4, 8, 16 and 32 edges. RI outperforms all other algorithms.

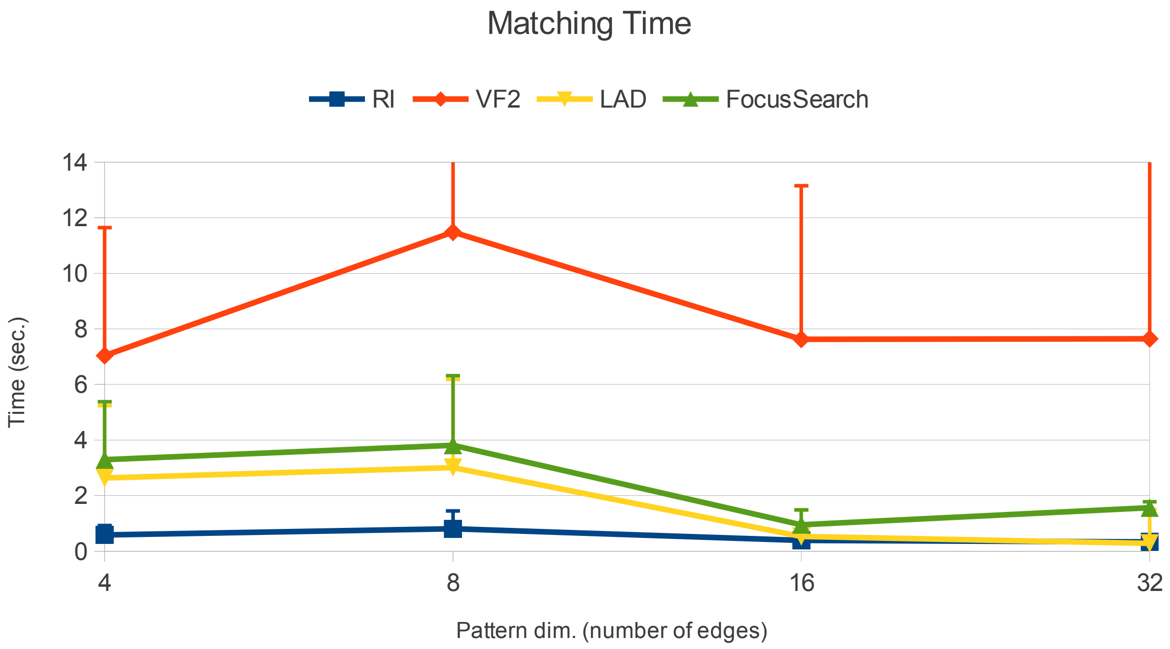

Figure 13: Averages of matching time on *AIDS* dataset for different patterns having 4, 8, 16 and 32 edges. RI outperforms all other algorithms.

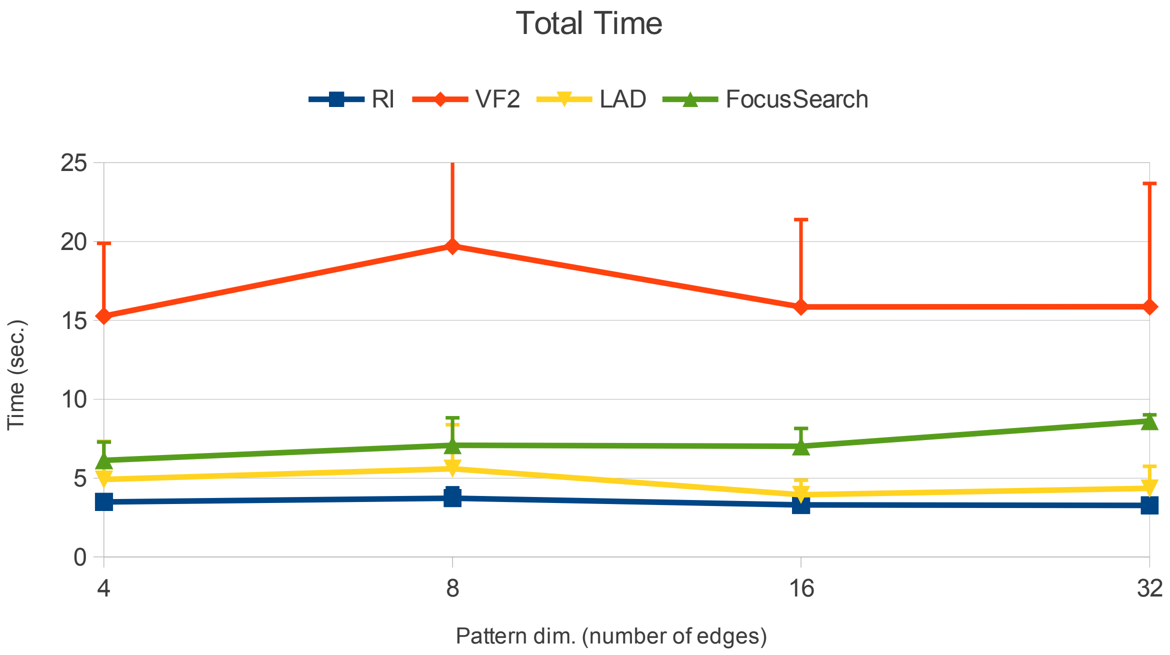

Figure 14: Averages of the total times on *AIDS* dataset for different pattern graph dimensions having 4, 8, 16 and 32 edges. The ratio between the size of the generated search space and the time needed to explore the search space allows RI to outperform all other methods.

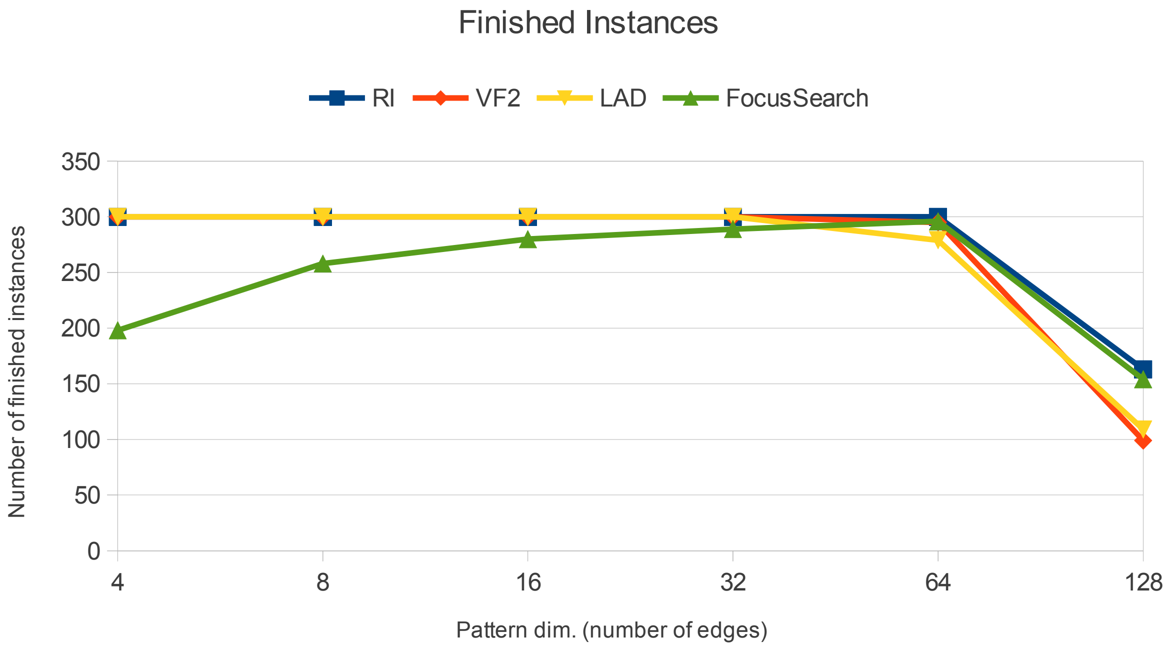

Figure 15: Number of pattern subgraph isomorphisms completed by the algorithms before the set time-out on *PDBSv1* dataset.

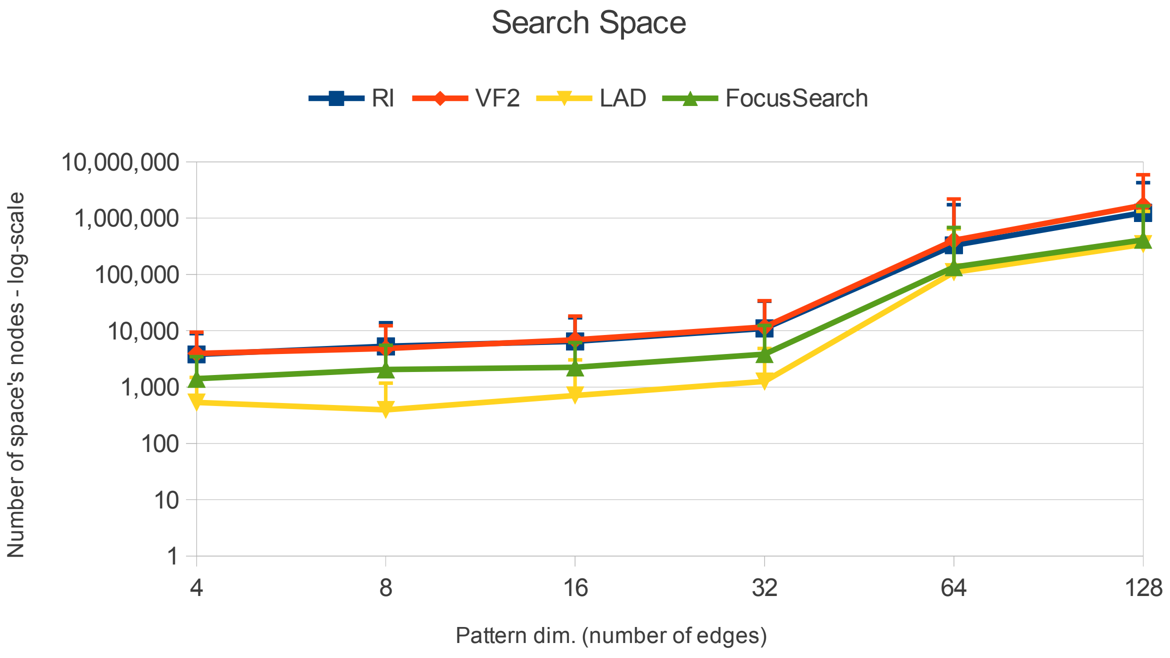

Figure 16: Averages of search space sizes on *PDBSv1* dataset for different patterns having 4, 8, 16 and 32, 64 and 128 edges. The chart shows the number of visited nodes. Reduction based methods (LAD and FocusSearch) outperform RI and VF2.

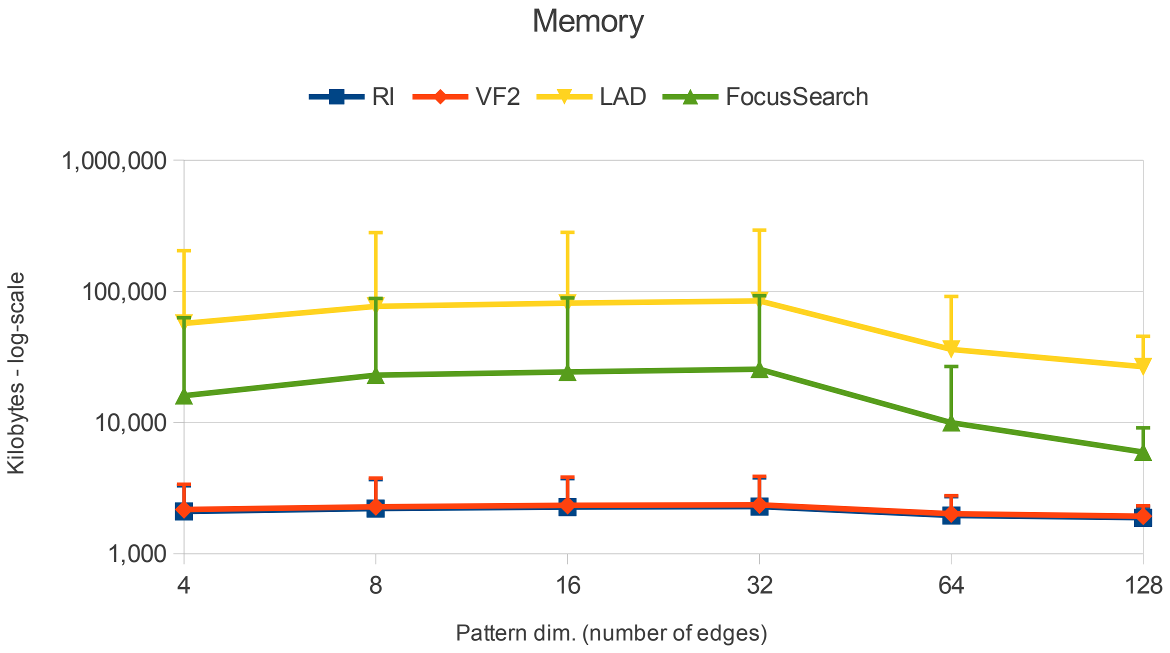

Figure 17: Averages of memory requirement on *PDBSv1* dataset for different patterns having 4, 8, 16 and 32, 64 and 128 edges. RI outperforms LAD and FocusSearch algorithms.

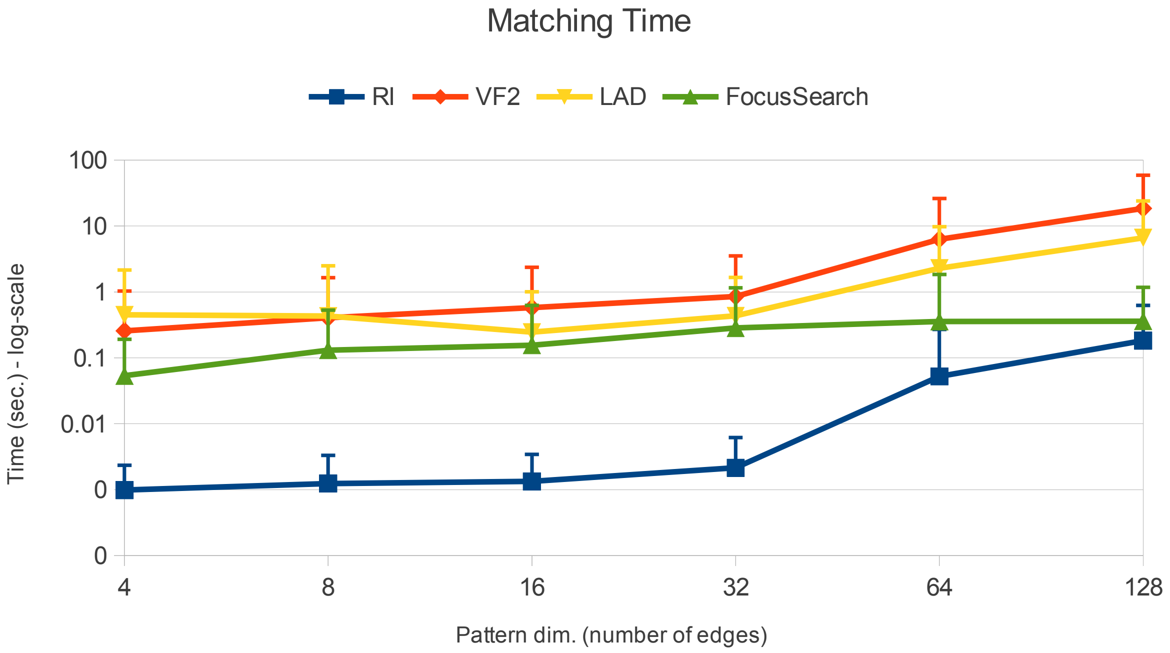

Figure 18: Averages of matching time on *PDBSv1* dataset for different patterns having 4, 8, 16 and 32, 64 and 128 edges. RI outperforms all other algorithms.

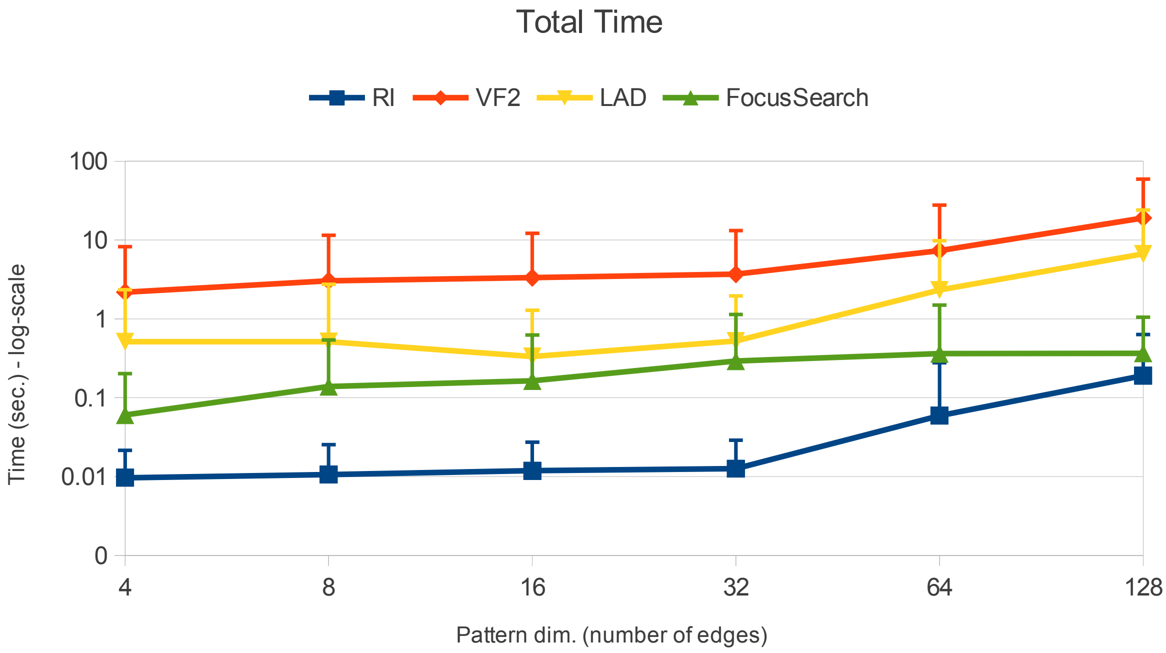

Figure 19: Averages of the total times on *PDBSv1* dataset for different pattern graph dimensions having 4, 8, 16 and 32, 64 and 128 edges. The ratio between the size of the generated search space and the time needed to explore the search space allows RI to outperform all other methods.

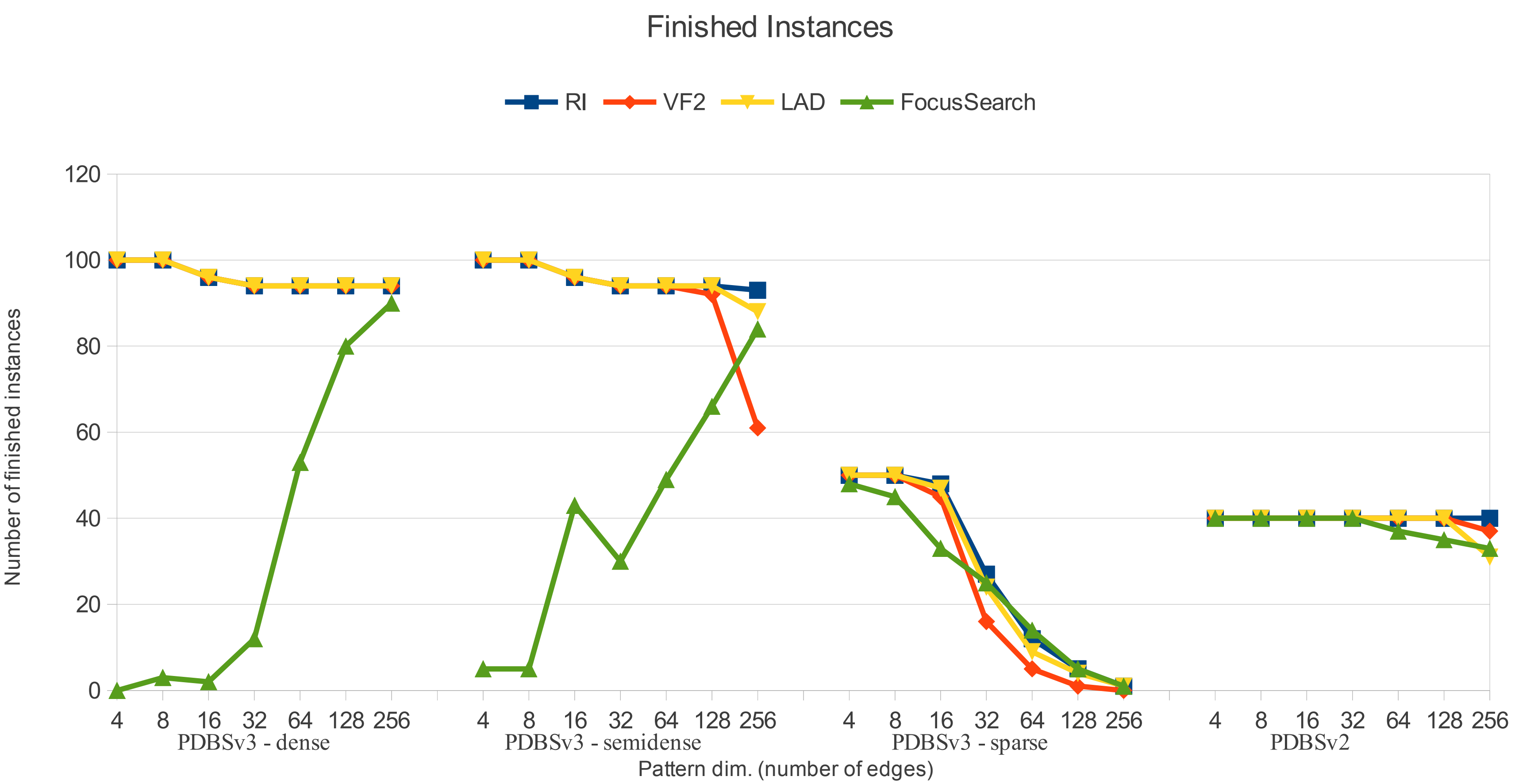

Figure 20: Number of pattern subgraph isomorphisms completed by the algorithms before the set time-out on *PDBSv2* and *PDBSv3* datasets.

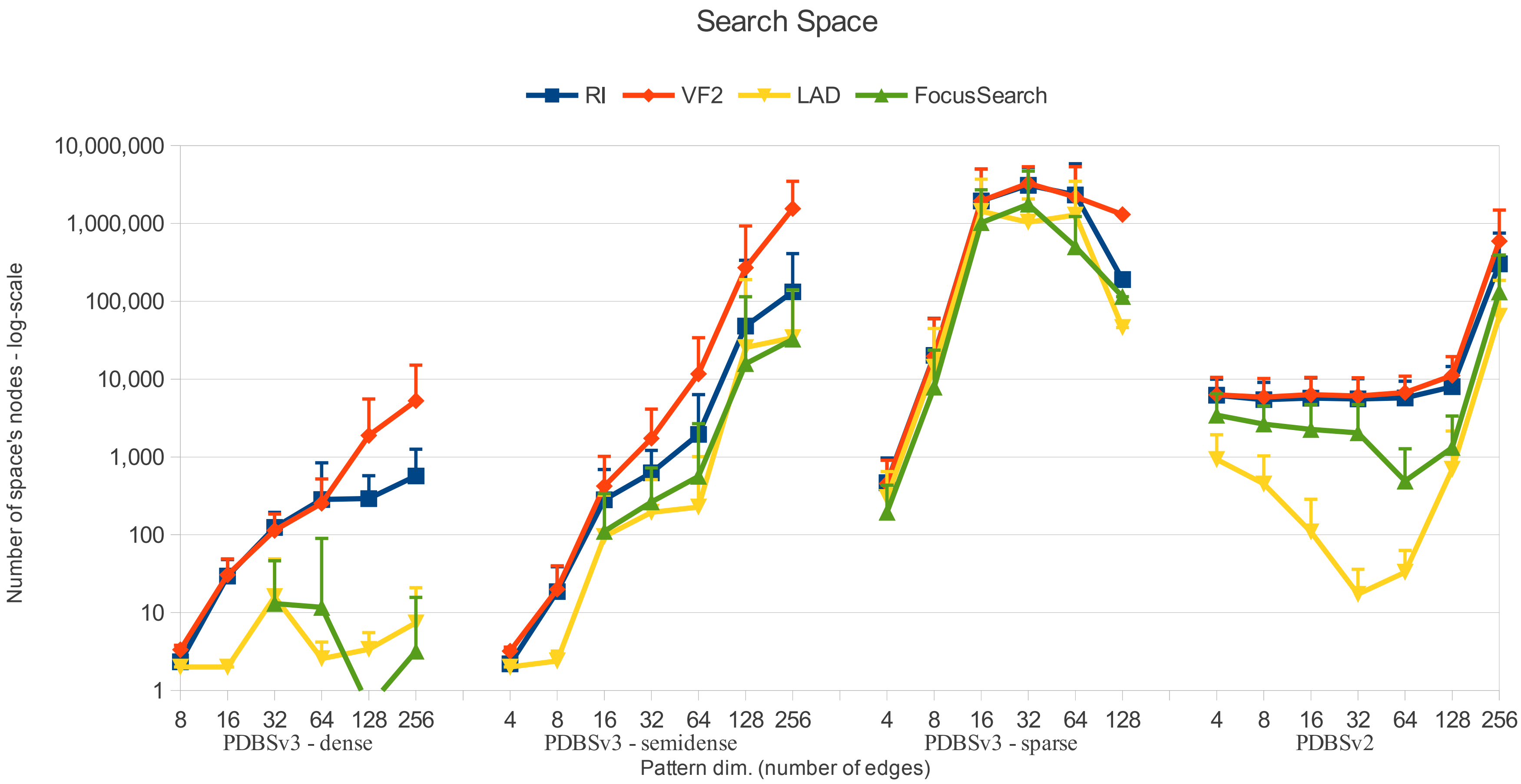

Figure 21: Averages of the search spaces sizes on *PDBSv2* and *PDBSv3* datasets. The chart shows the number of visited nodes. Results are divided by pattern density and pattern dimension. Dense indicates that the number of vertices of the pattern is 50% of the number of pattern edges. Semidense indicates that the number of vertices of the pattern is the 25% of the number of pattern edges. In the sparse patterns the number vertices of the pattern is the 90% of the number of pattern edges. Sparse patterns on contact maps,*PDBSv3*, (which are dense graphs) generate a larger search space for all the methods. However, the reduction procedures of FocusSearch and LAD outperform the other methods in particular on medium sparse graphs (*PDBSv2*) at the cost of memory usage (see next plot-Memory).

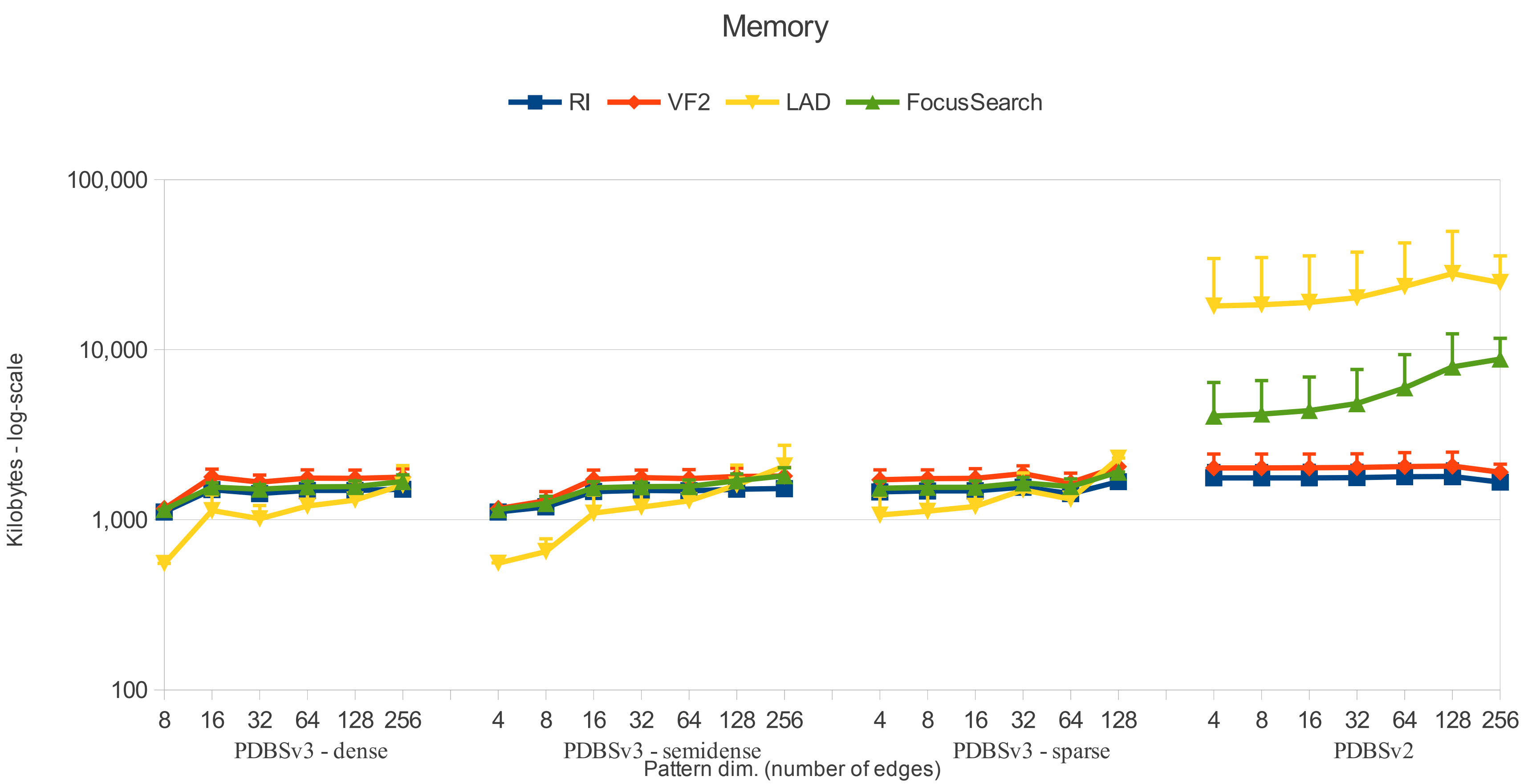

Figure 22: Averages of memory requirements on *PDBSv2* and *PDBSv3* datasets. The chart measures the maximum peaks of memory usage. Results are divided by pattern density and pattern dimension (number of edges). Dense indicates that the number of vertices of the pattern is 50% of the number of pattern edges. Semidense indicates that the number of vertices of the pattern is the 25% of the number of pattern edges. In the sparse patterns the number vertices of the pattern is the 90% of the number of pattern edges. On medium sparse graphs, *PDBSv2*, domain-based methods require much memory to store domains and compatibility maps. This makes it possible for them to have a low search space (see the above plot-Search Space).

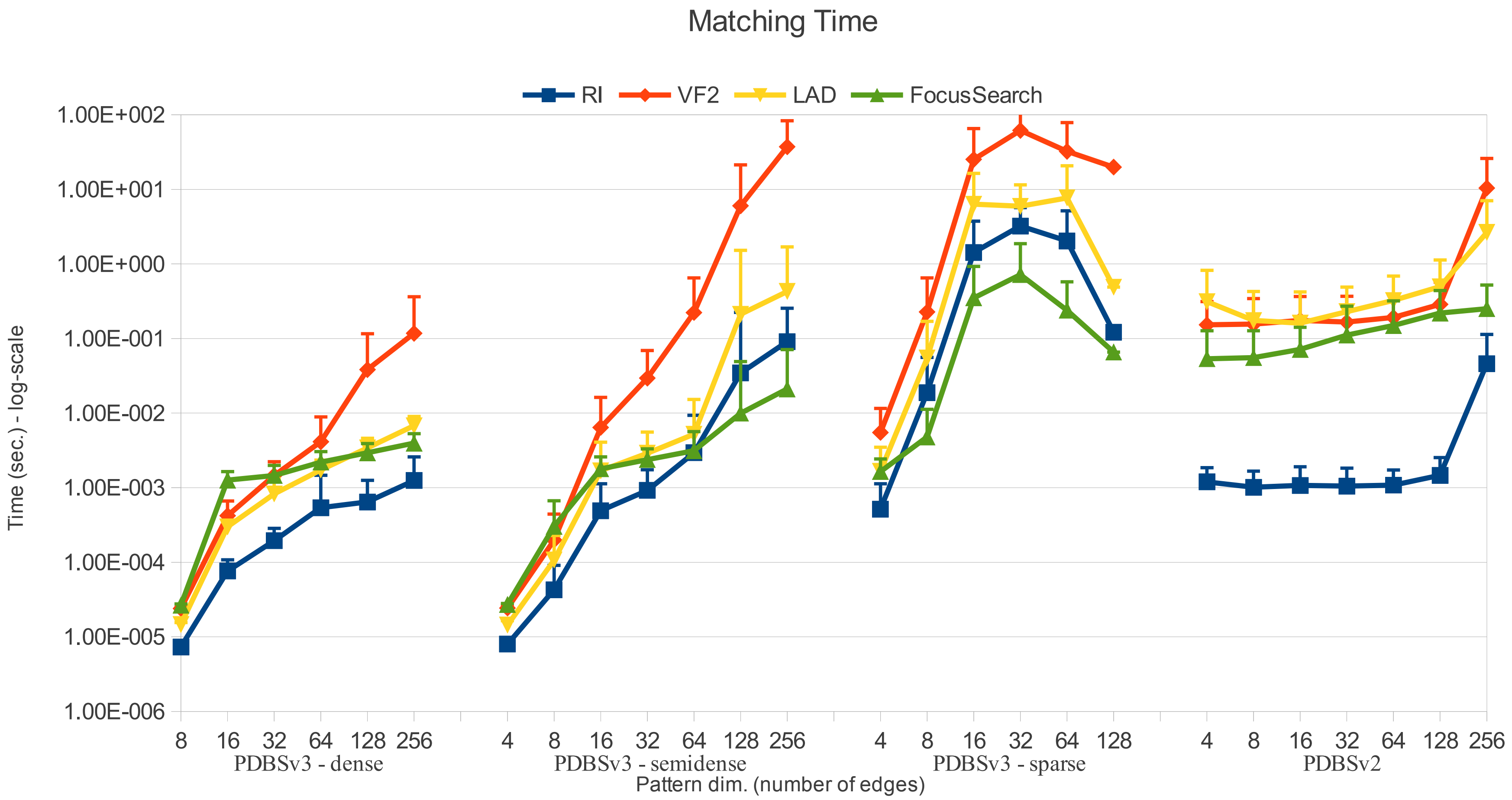

Figure 23: Averages of matching times on *PDBSv2* and *PDBSv3* datasets. Results are divided by target type, patterns density and patterns dimension. Dense indicates that the number of vertices of the pattern is 50% of the number of pattern edges. Semidense indicates that the number of vertices of the pattern is the 25% of the number of pattern edges. In the sparse patterns the number vertices of the pattern is the 90% of the number of pattern edges. The RI search strategy keeps RI competitive on contact maps (*PDBSv3*) and outperforms other methods on sparse targets such as proteins backbones (*PDBSv2*).

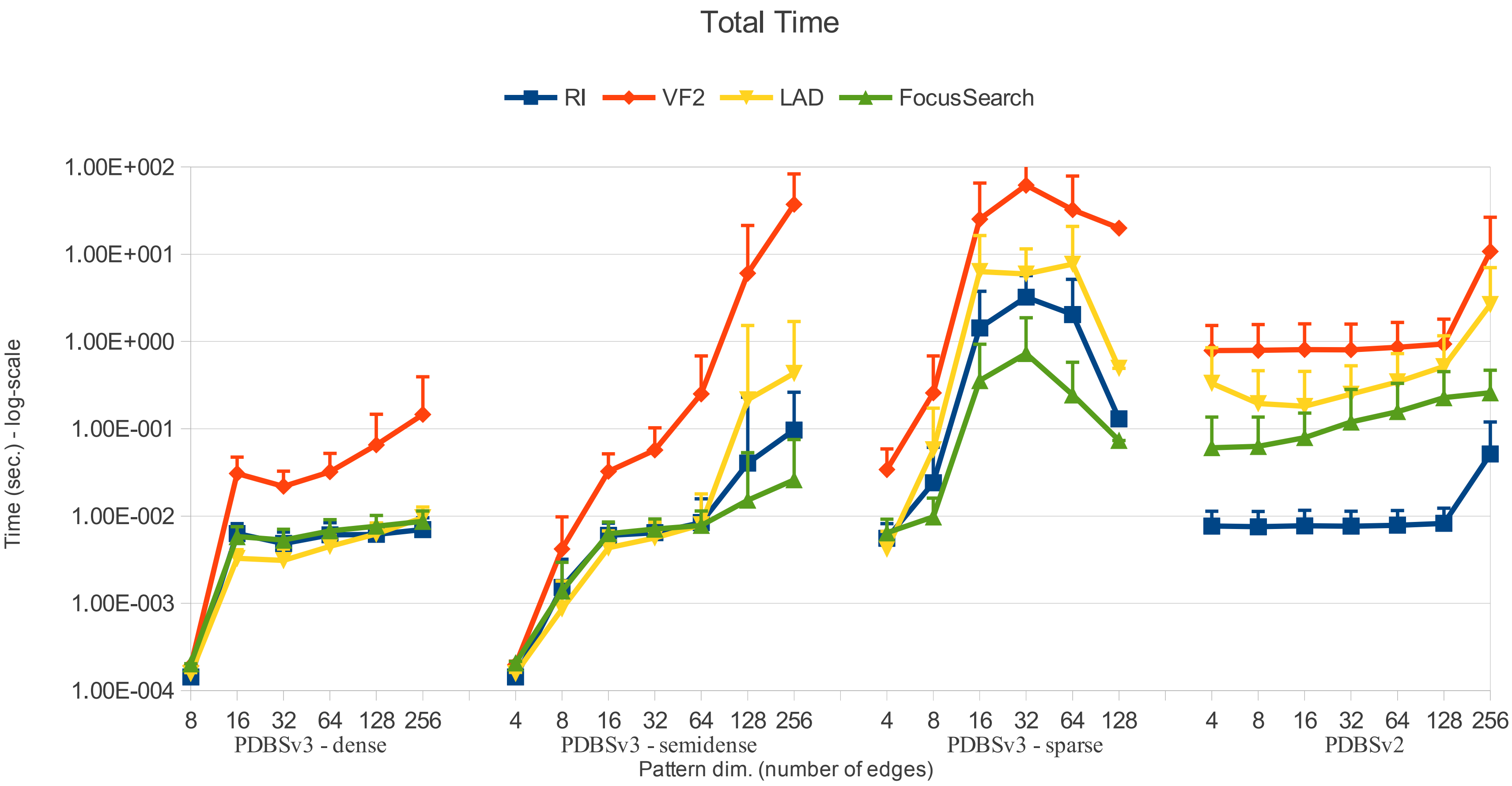

Figure 24: Averages of total times on *PDBSv2* and *PDBSv3* datasets. Results are divided by target type, patterns density and patterns dimension. Dense indicates that the number of vertices of the pattern is 50% of the number of pattern edges. Semidense indicates that the number of vertices of the pattern is the 25% of the number of pattern edges. In the sparse patterns the number vertices of the pattern is the 90% of the number of pattern edges. The RI search strategy keeps RI competitive on contact maps (*PDBSv3*) and outperforms other methods on sparse targets such as proteins backbones (*PDBSv2*).

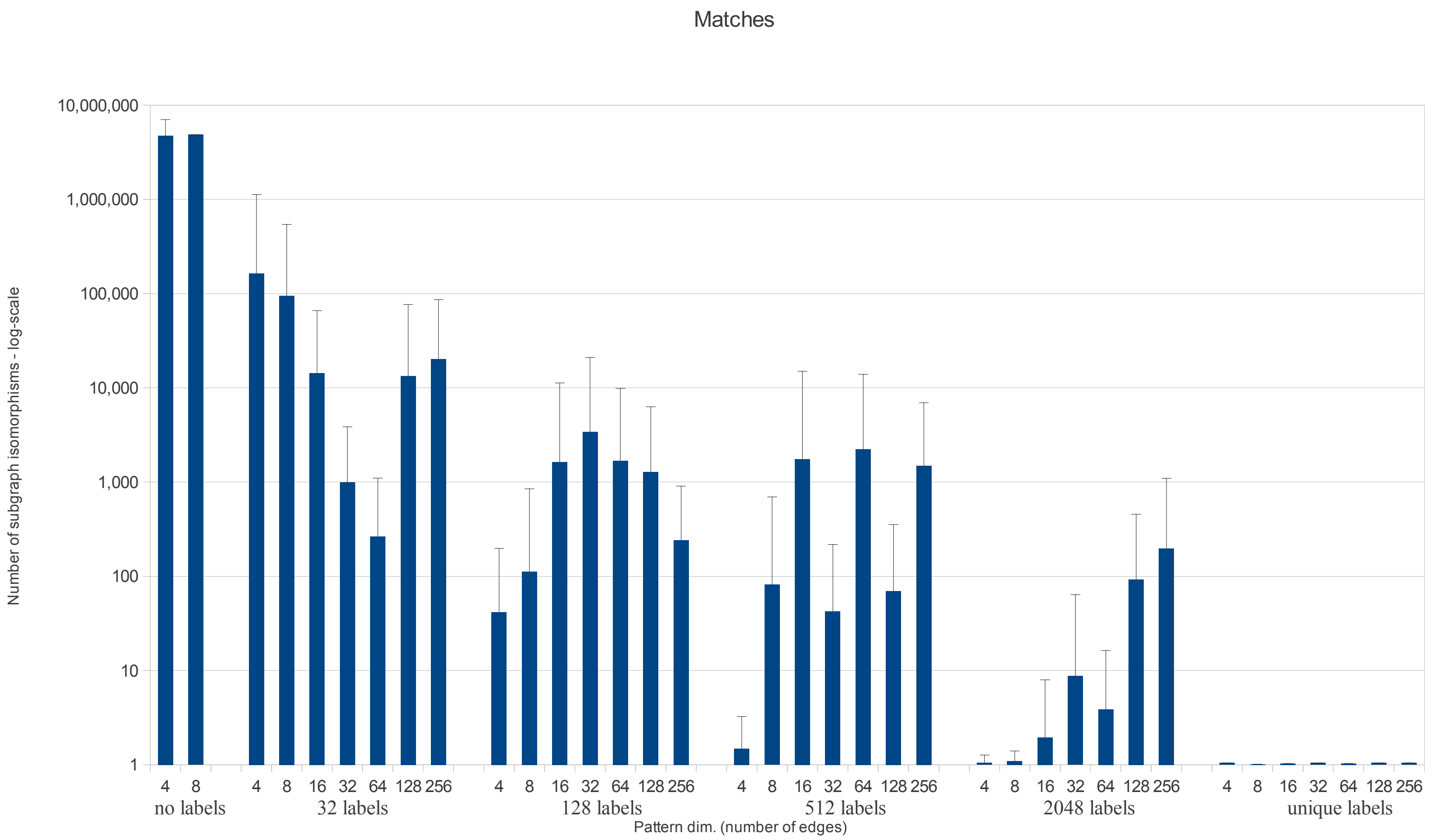

Figure 25: Number of pattern subgraph isomorphisms on *Graemlin* dataset

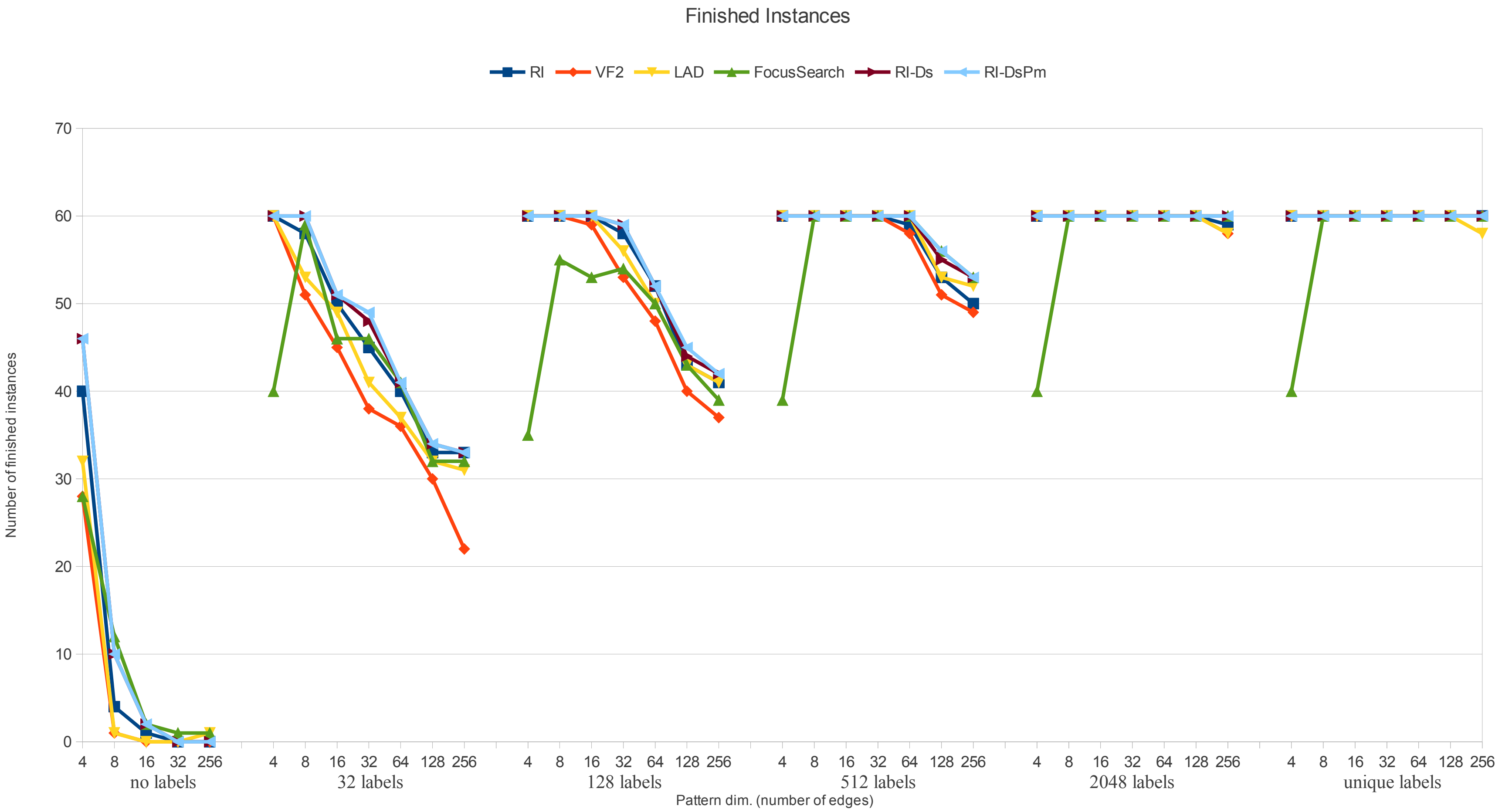

Figure 26: Number of pattern subgraph isomorphisms completed by the algorithms before the set time-out on *Graemlin* dataset

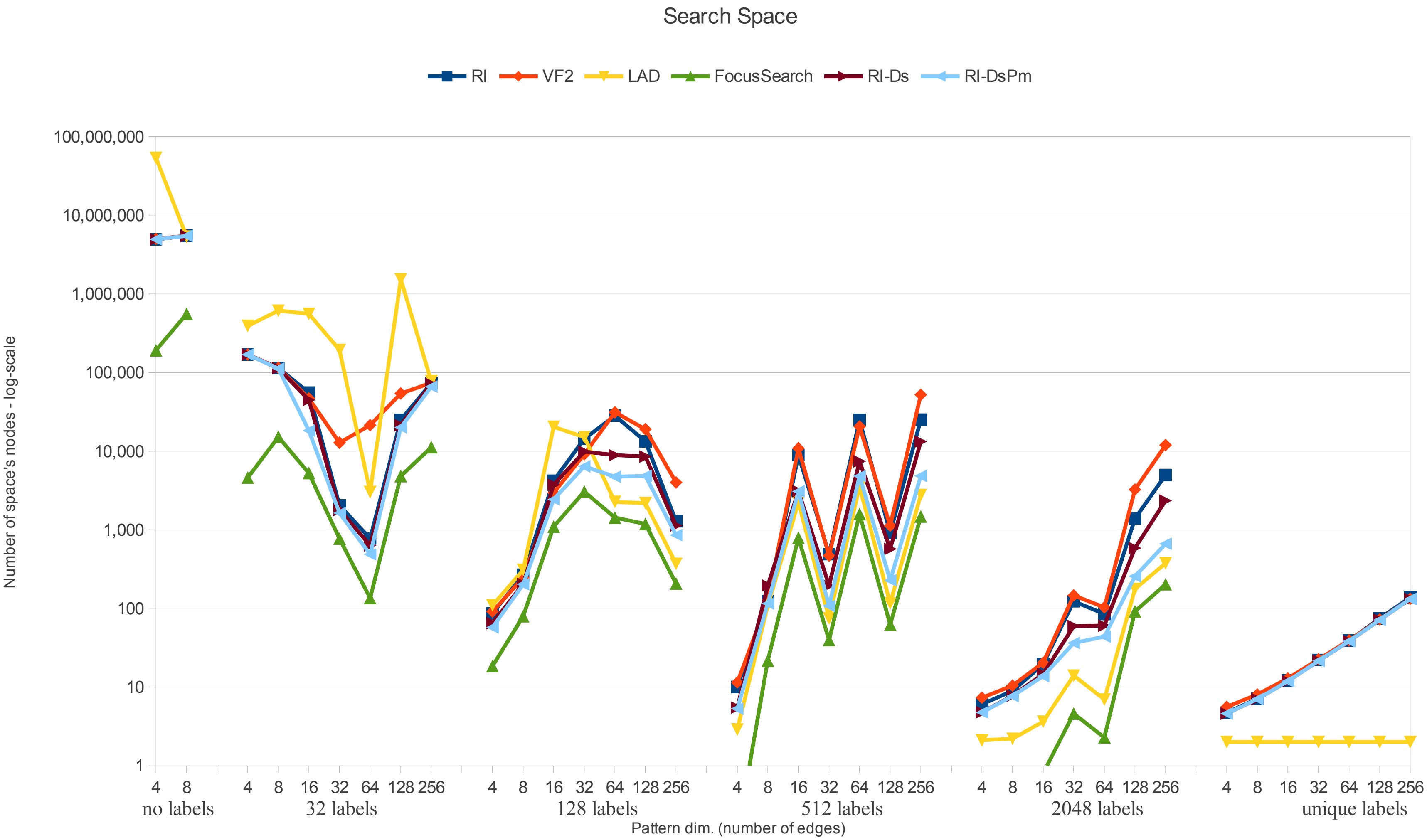

Figure 27: Averages of search space sizes on *Graemlin* dataset. The chart shows the number of visited search tree nodes. Networks are randomly uniformly labeled with 32, 64, 128, 512, 2048 and all different labels. Results are grouped by number of labels in the dataset and pattern dimension (number of edges). FocusSearch outperforms all other systems but LAD on the dataset labeled with unique labels.

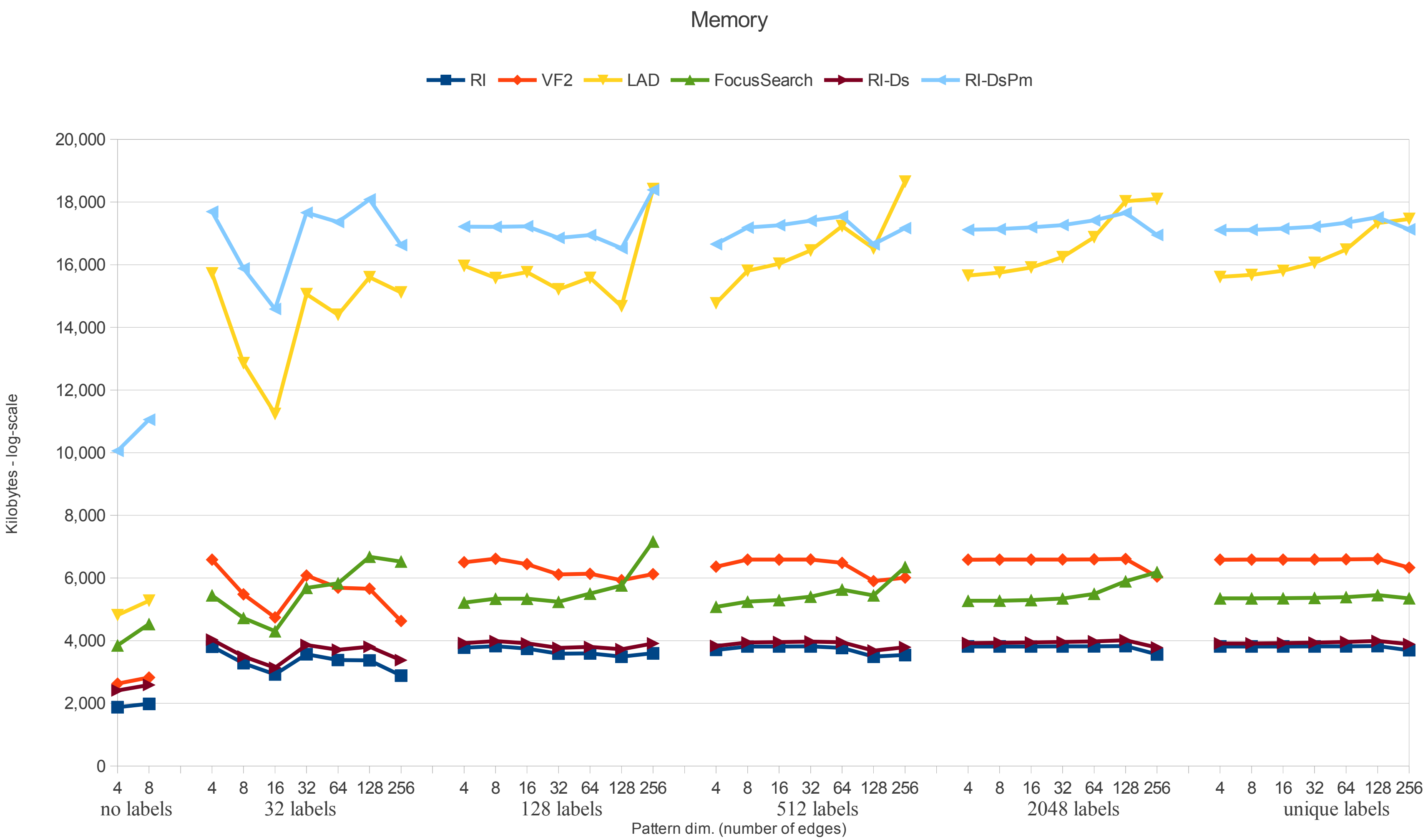

Figure 28: Averages of memory requirements for the *Graemlin* datasets. Networks are randomly uniformly labeled with 32, 64, 128, 512, 2048 and all different labels. Results are grouped by number of labels in the dataset and pattern dimension (number of edges). The chart measures the peaks of memory usage during the execution of the algorithms. The large number of nodes in the target graphs does not make the memory usage in LAD explode in contrast to the amount of memory requirement to store compatibility maps and domains. However, thanks to the bit vector domain representation, FocusSearch can maintain a reasonably low memory usage. RI outperforms all other systems.

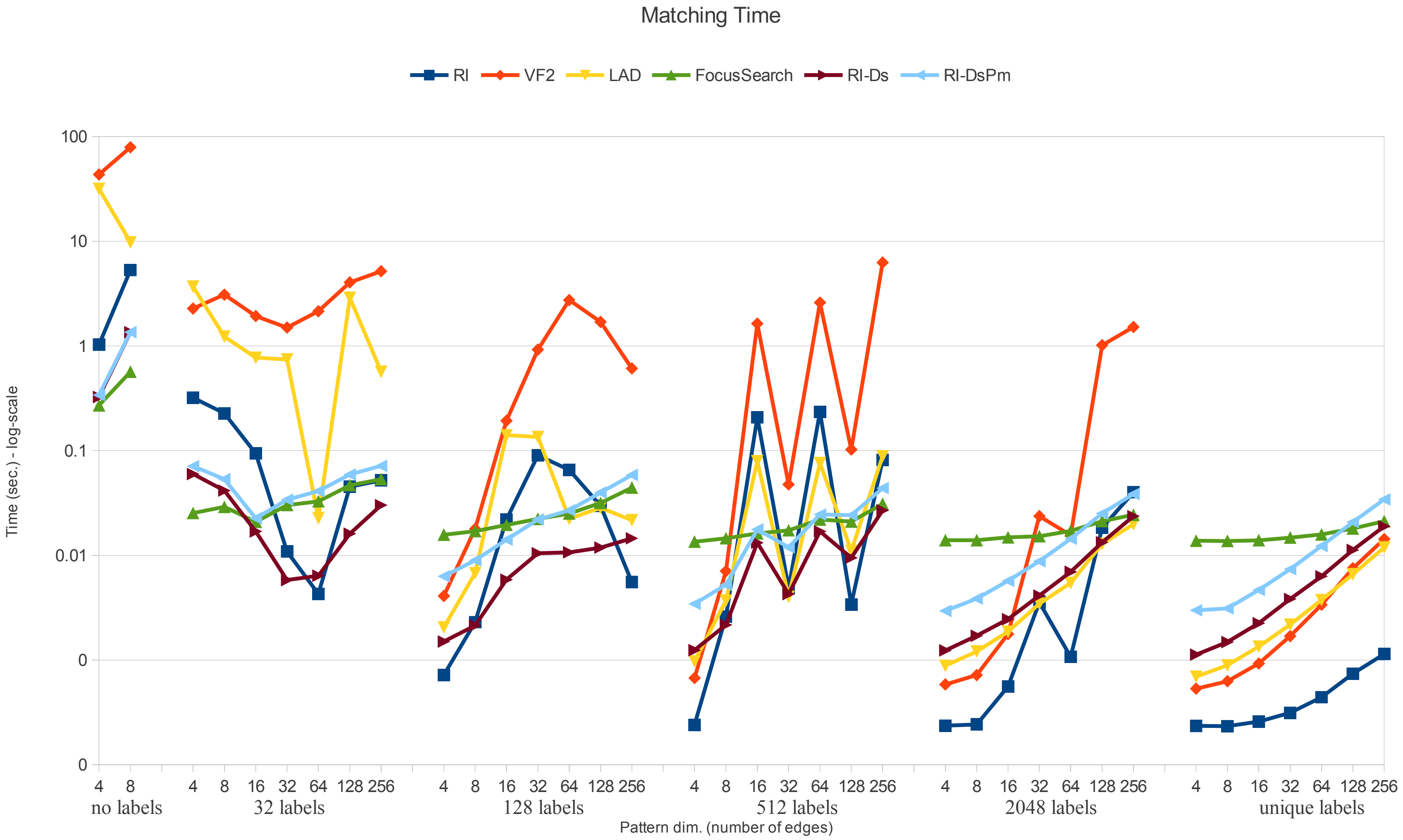

Figure 29: Averages of matching times on *Graemlin* dataset. Networks are randomly uniformly labeled with 32, 64, 128, 512, 2048 and all different labels. Results are grouped by number of labels in the dataset and pattern dimension (number of edges). RI outperforms the other methods by increasing the number of labels. For a small number of labels RI-Ds outperforms other systems.

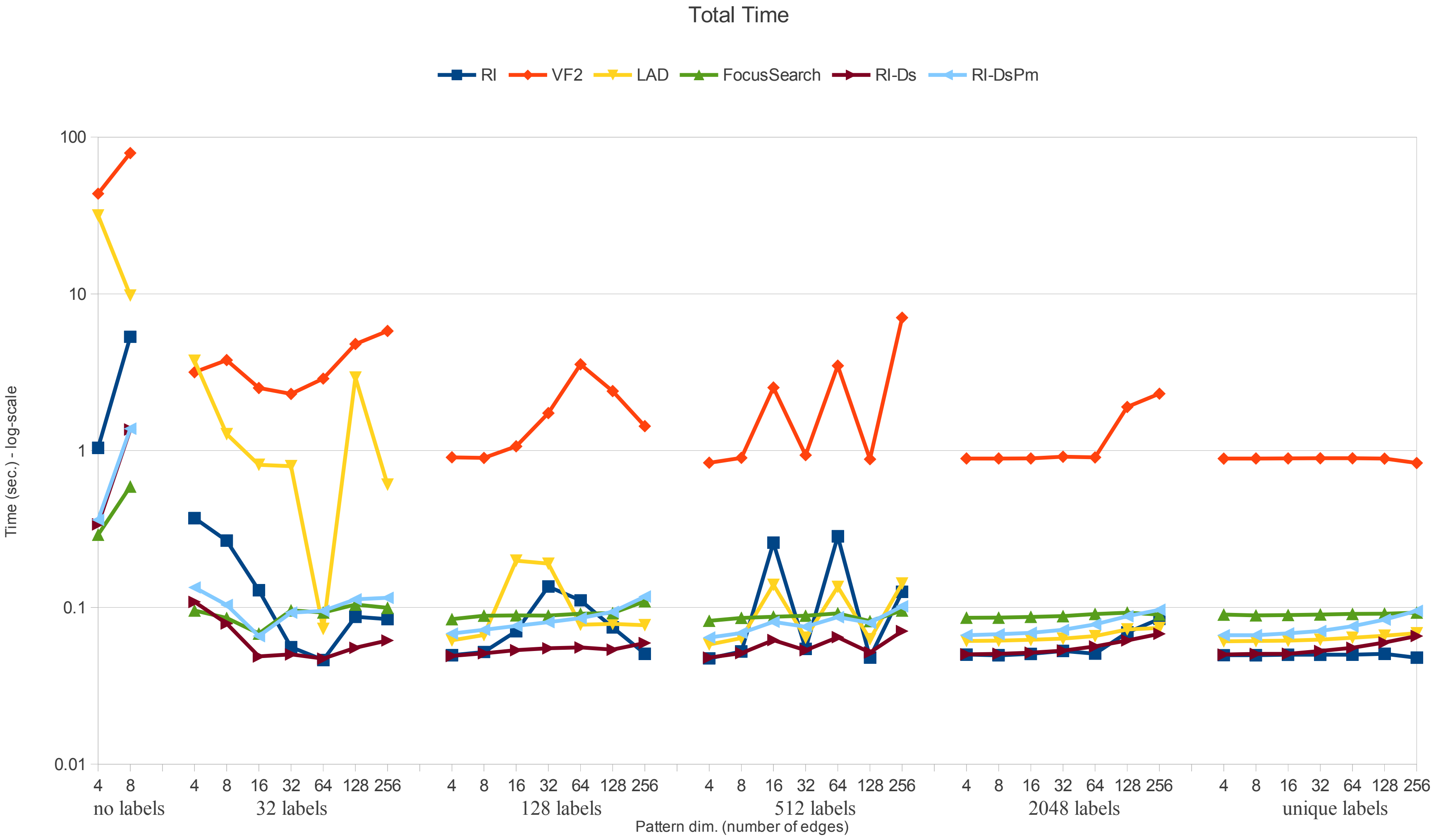

Figure 30: Averages of matching times on *Graemlin* dataset. Networks are randomly uniformly labeled with 32, 64, 128, 512, 2048 and all different labels. Results are grouped by number of labels in the dataset and pattern dimension (number of edges). RI-Ds is comparable with all other methods and outperforms LAD on a small number of labels.

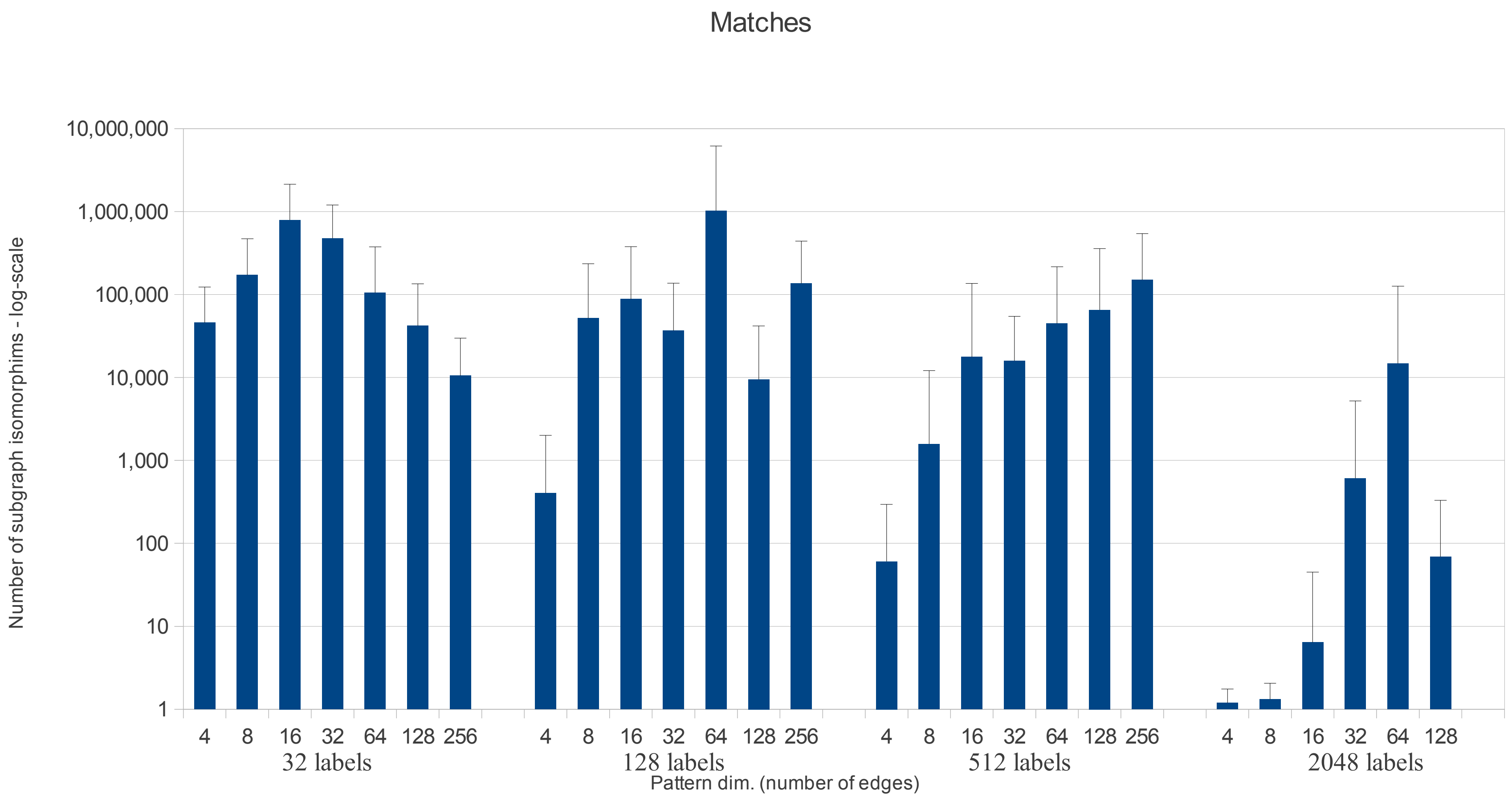

Figure 31: Number of subgraph isomorphisms on *PPI* dataset. The PPI networks are randomly labelled using a normal distribution.

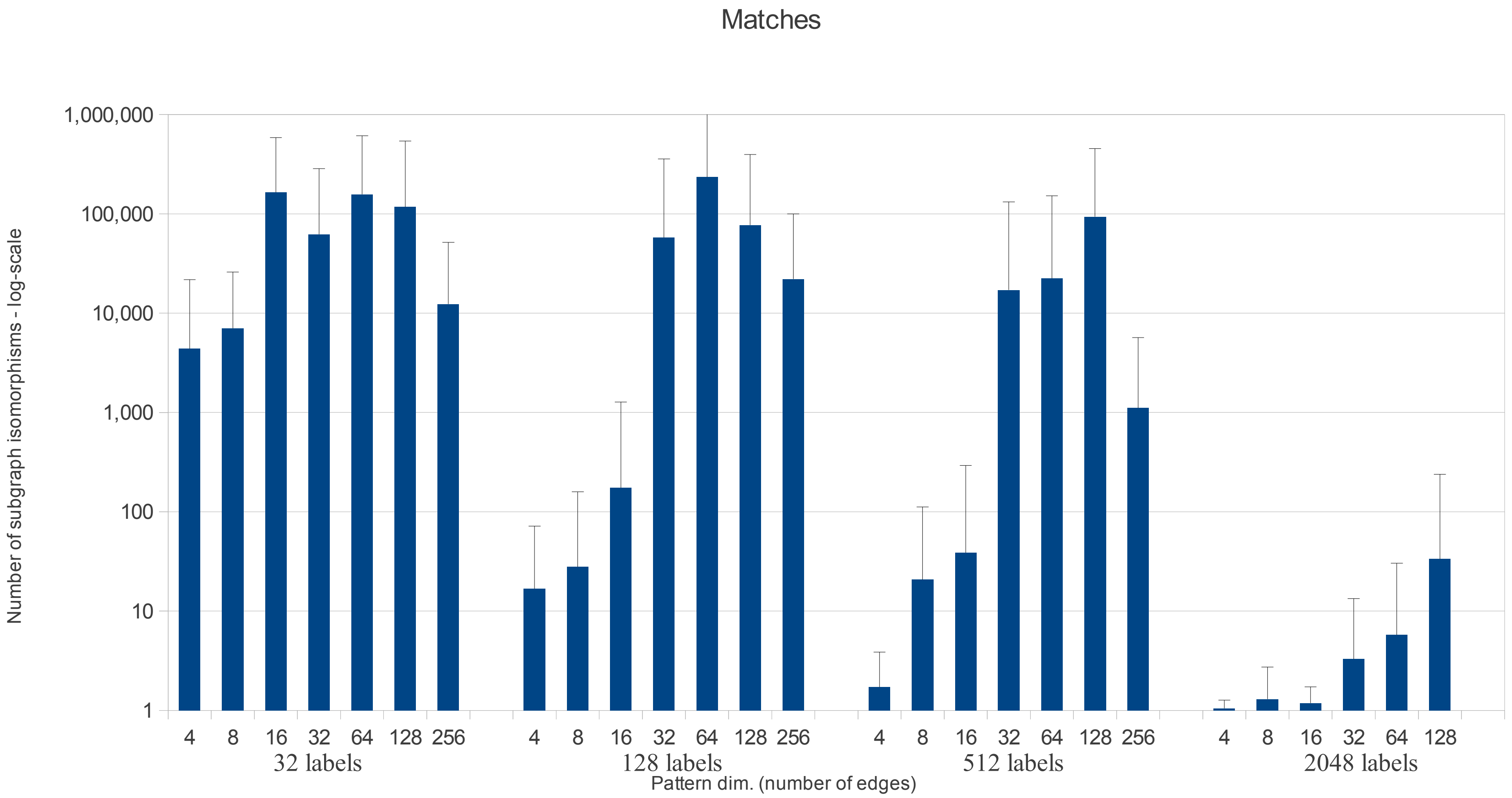

Figure 32: Number of subgraph isomorphisms on *PPI* dataset. The PPI networks are uniformly randomly labelled.

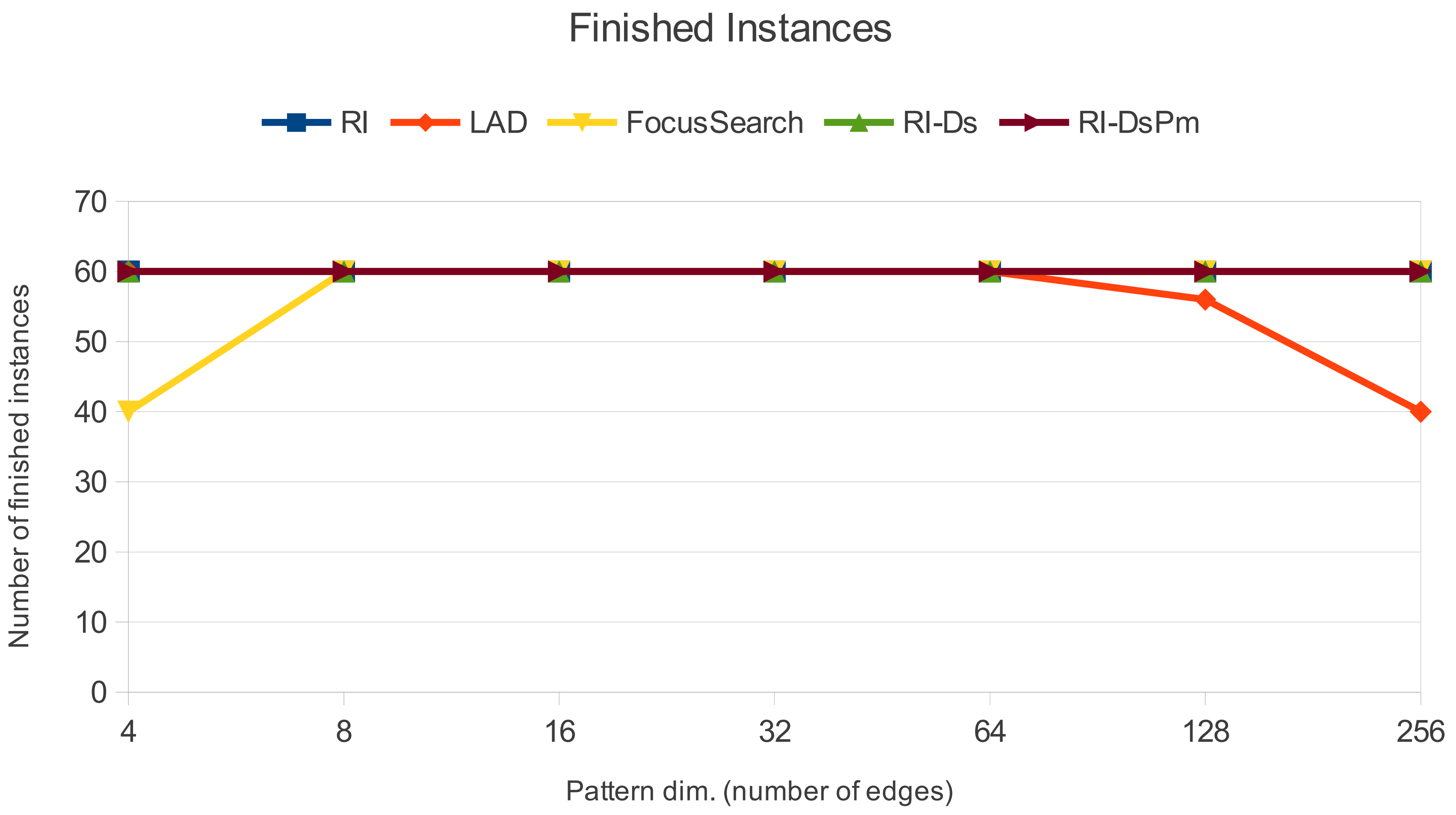

Figure 33: Number of pattern subgraph isomorphisms completed by the algorithms before the set time-out. The PPI networks are labeled with the names of proteins. Each vertex has a unique label.

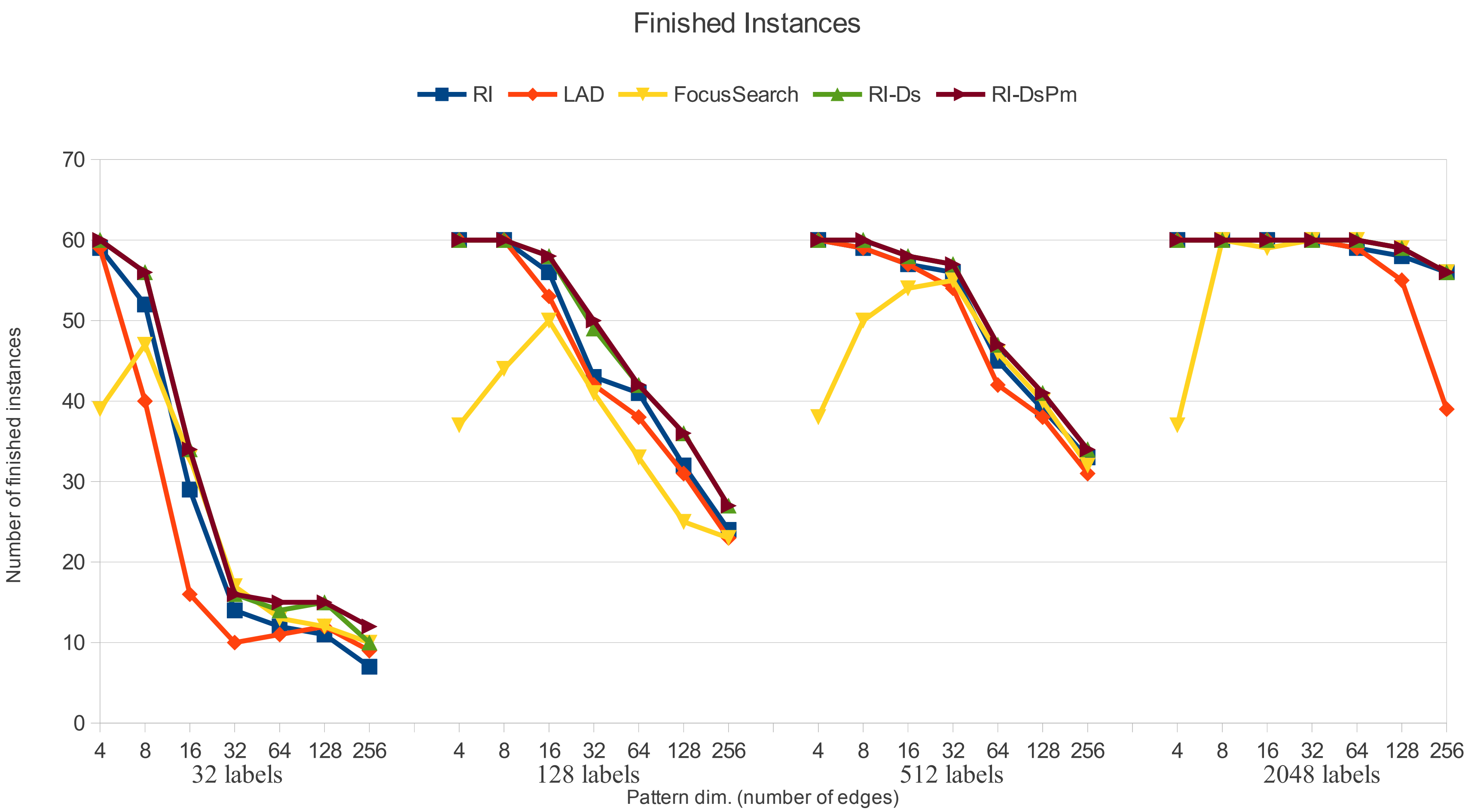

Figure 34: Number of pattern subgraph isomorphisms completed by the algorithms before the set time-out. The PPI networks are randomly labelled using a normal distribution.

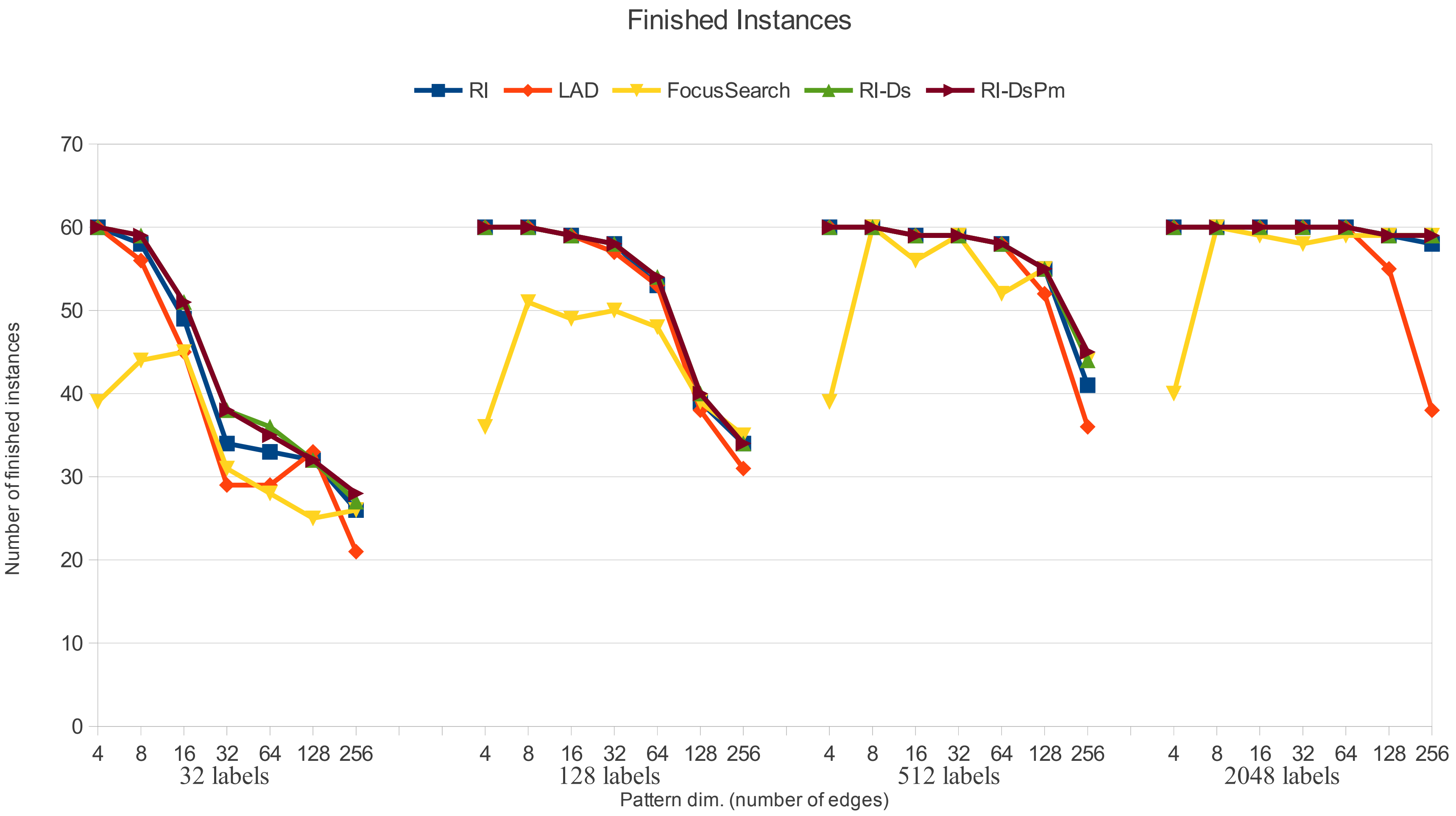

Figure 35: Number of pattern subgraph isomorphisms completed by the algorithms before the set time-out. The PPI networks are uniformly randomly labelled.

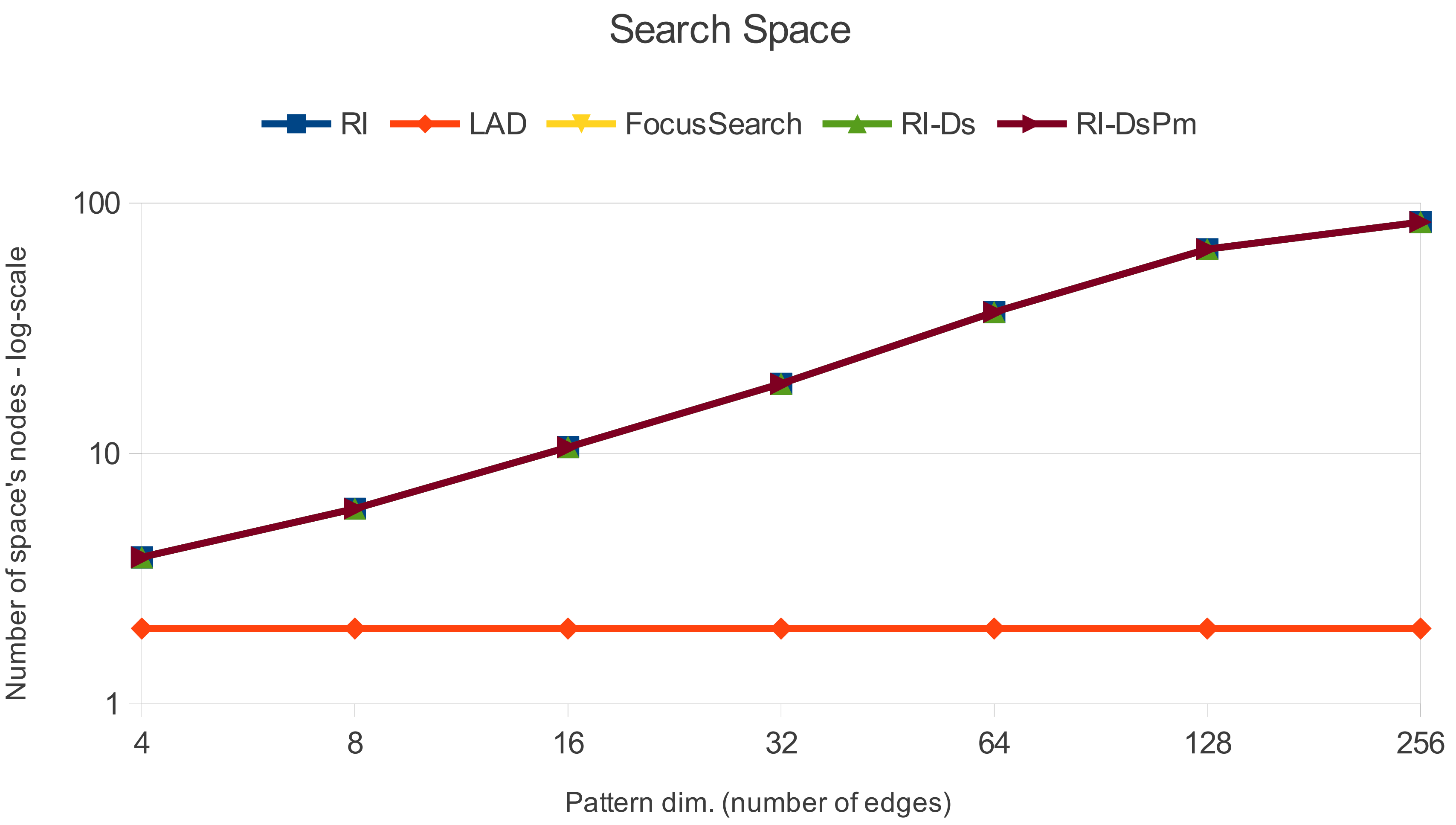

Figure 36: Averages of search space sizes on *PPI* dataset. The PPI networks are labeled with the names of proteins. Each vertex has a unique label. LAD outperforms all other systems.

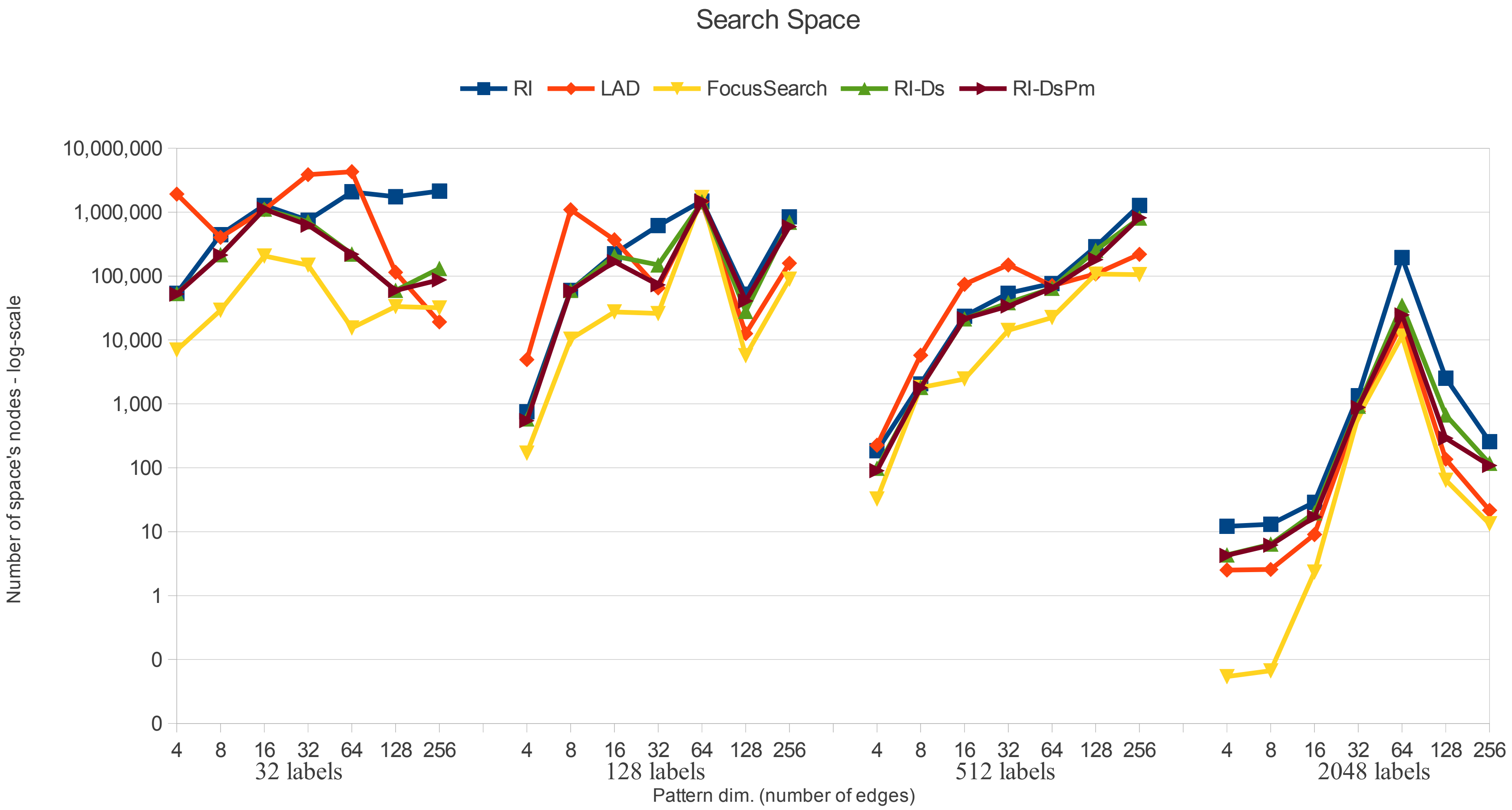

Figure 37: Averages of search space sizes on *PPI* dataset. The PPI networks are randomly labelled using a normal distribution. FocusSearch has the best performances.

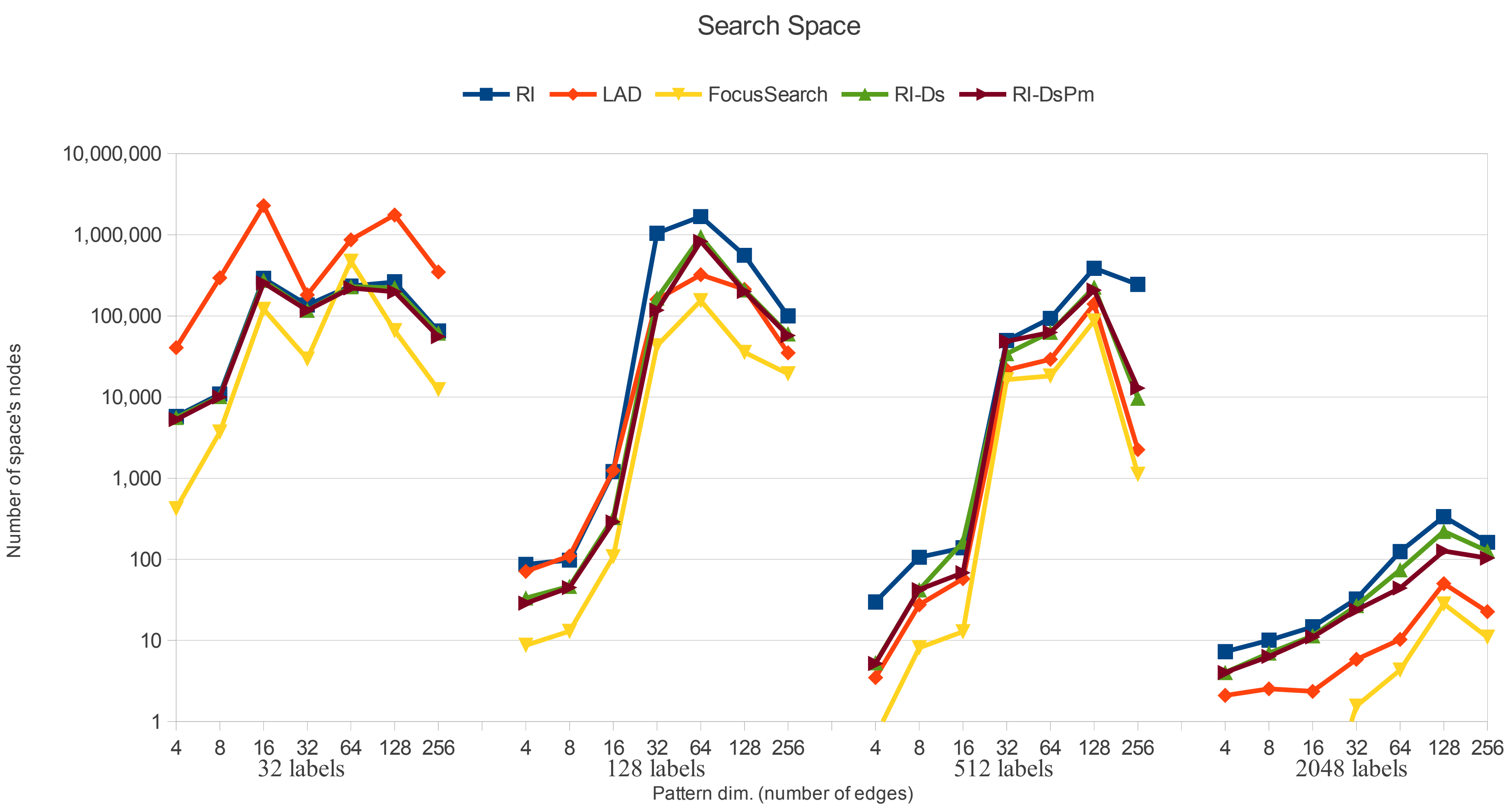

Figure 38: Averages of search space sizes on *PPI* dataset. The PPI networks are uniformly randomly labelled. FocusSearch has the best performances.

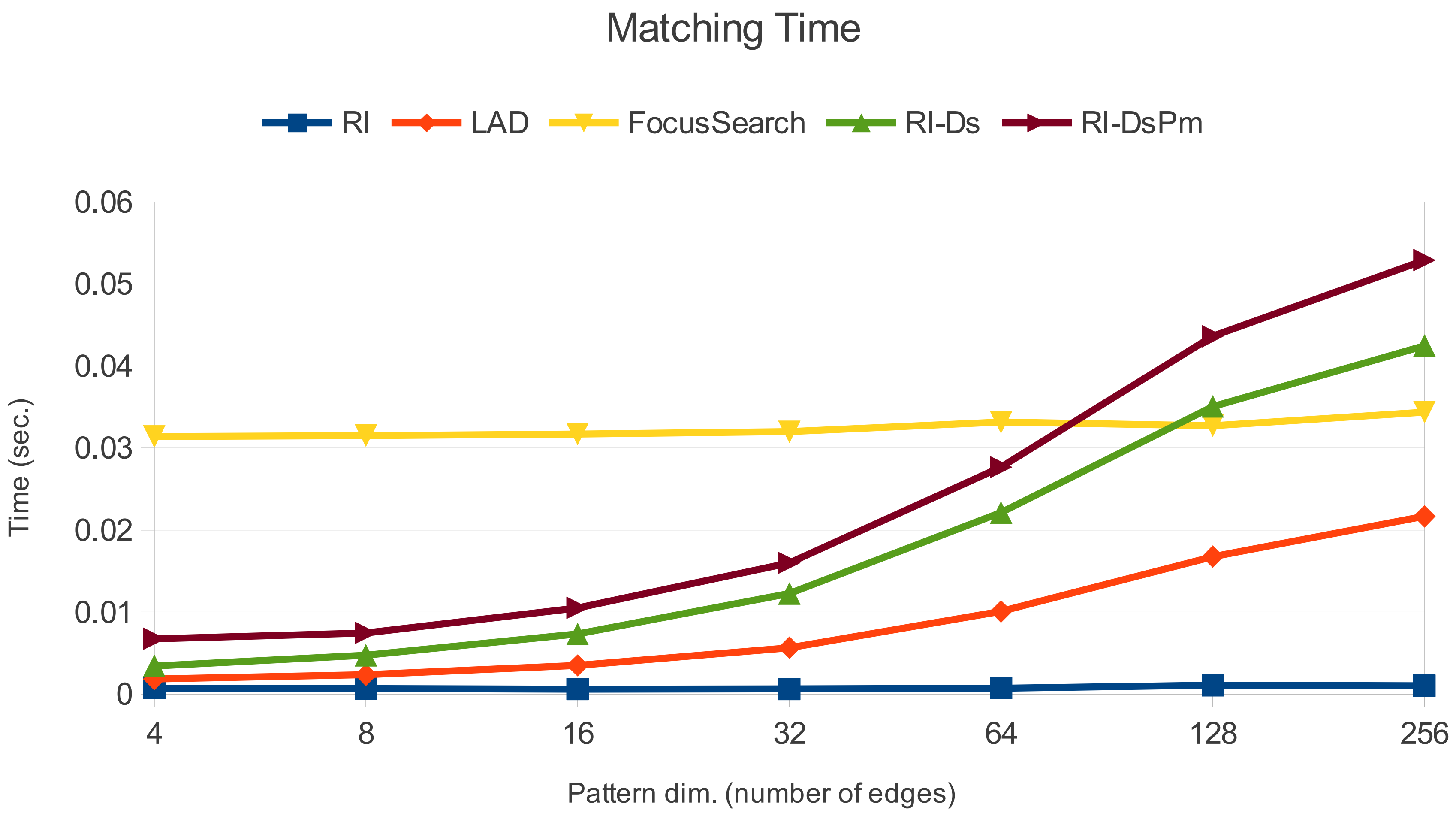

Figure 39: Averages of matching times on *PPI* dataset. The PPI networks are labeled with the names of proteins. Each vertex has a unique label. RI ouyperforms other systems.

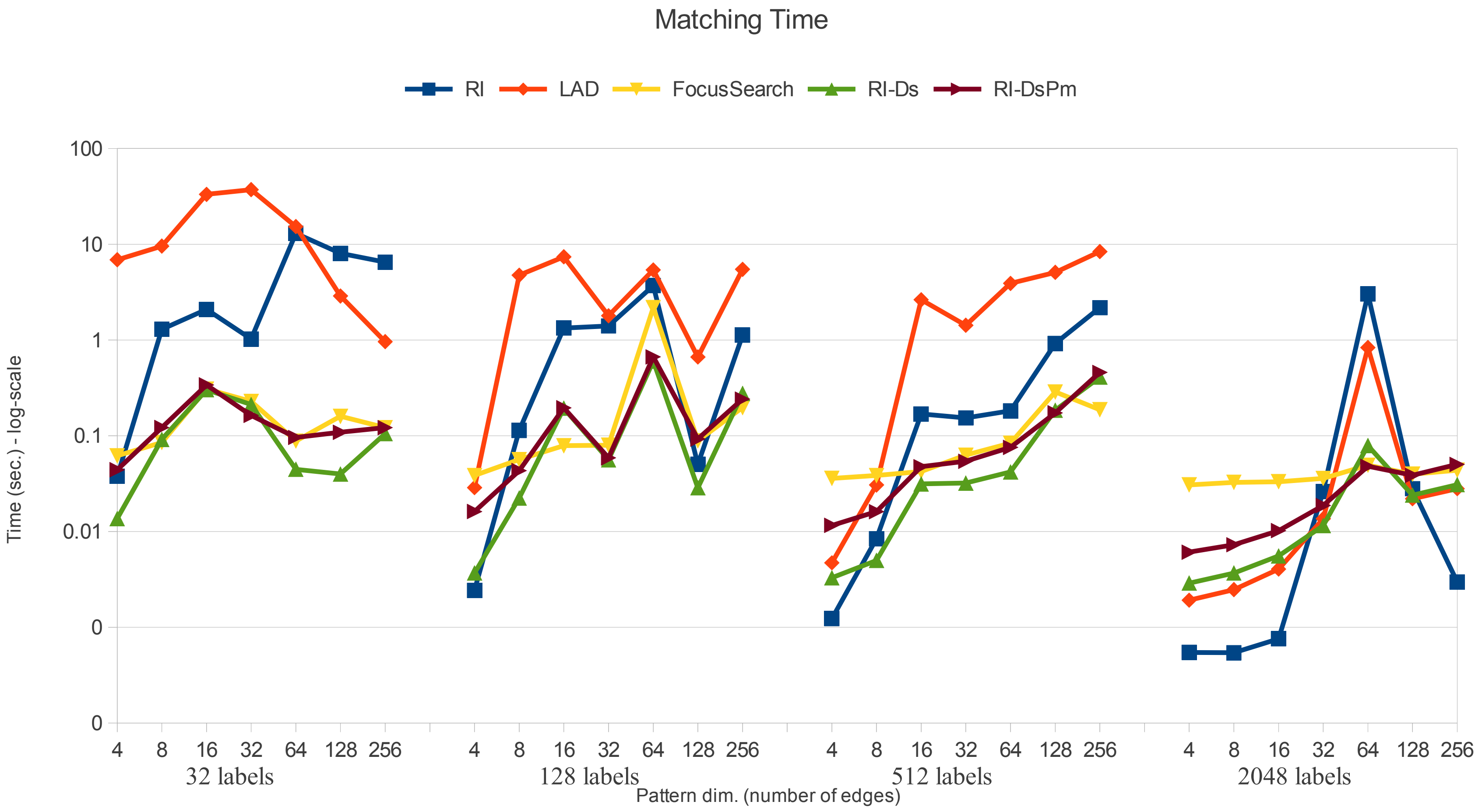

Figure 40: Averages of matching times on *PPI* dataset. The PPI networks are randomly labelled using a normal distribution. RI-Ds outperforms all other systems.

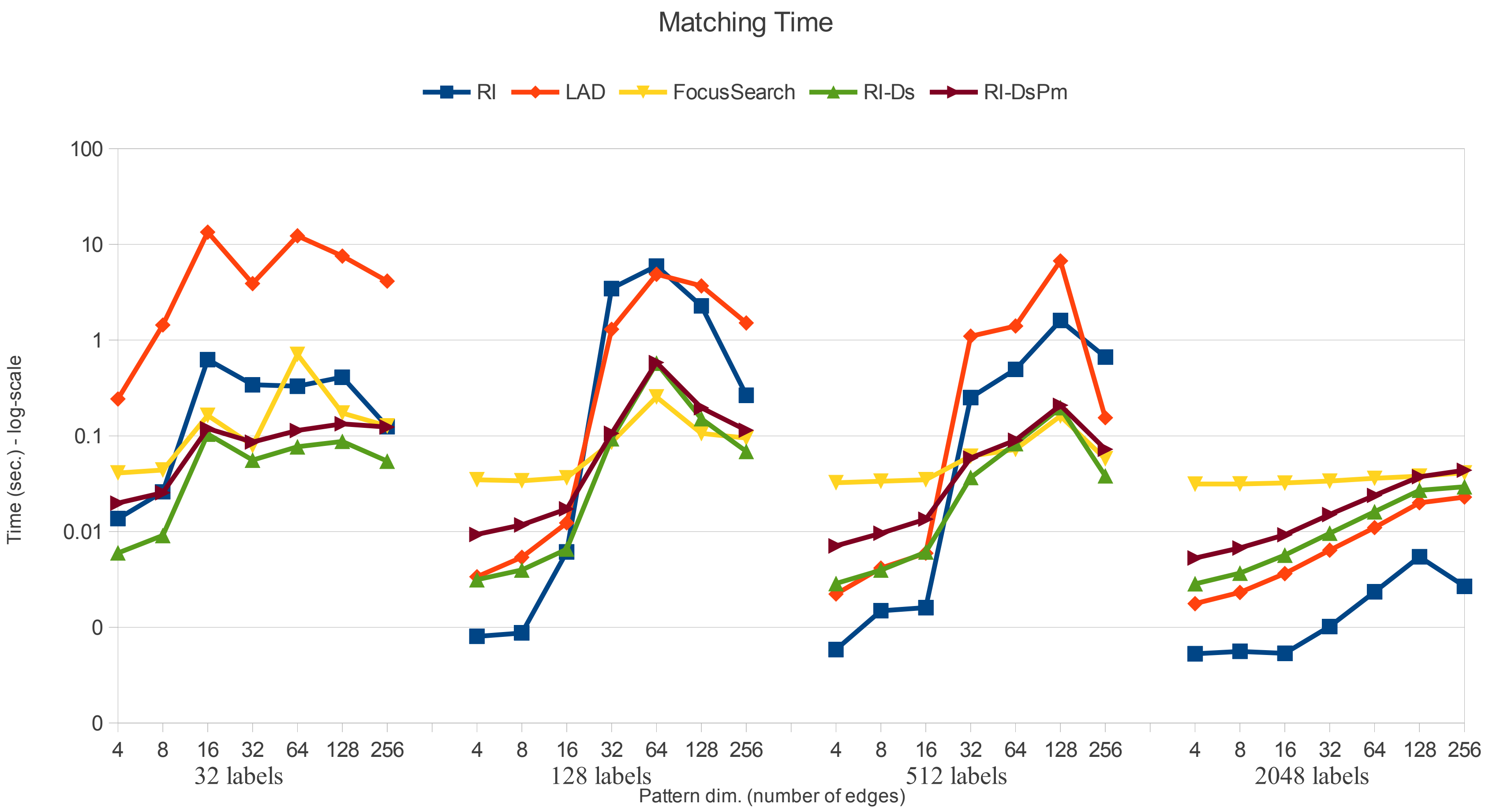

Figure 41: Averages of matching times on *PPI* dataset. The PPI networks are uniformly randomly labelled. RI-Ds outperforms all other systems.

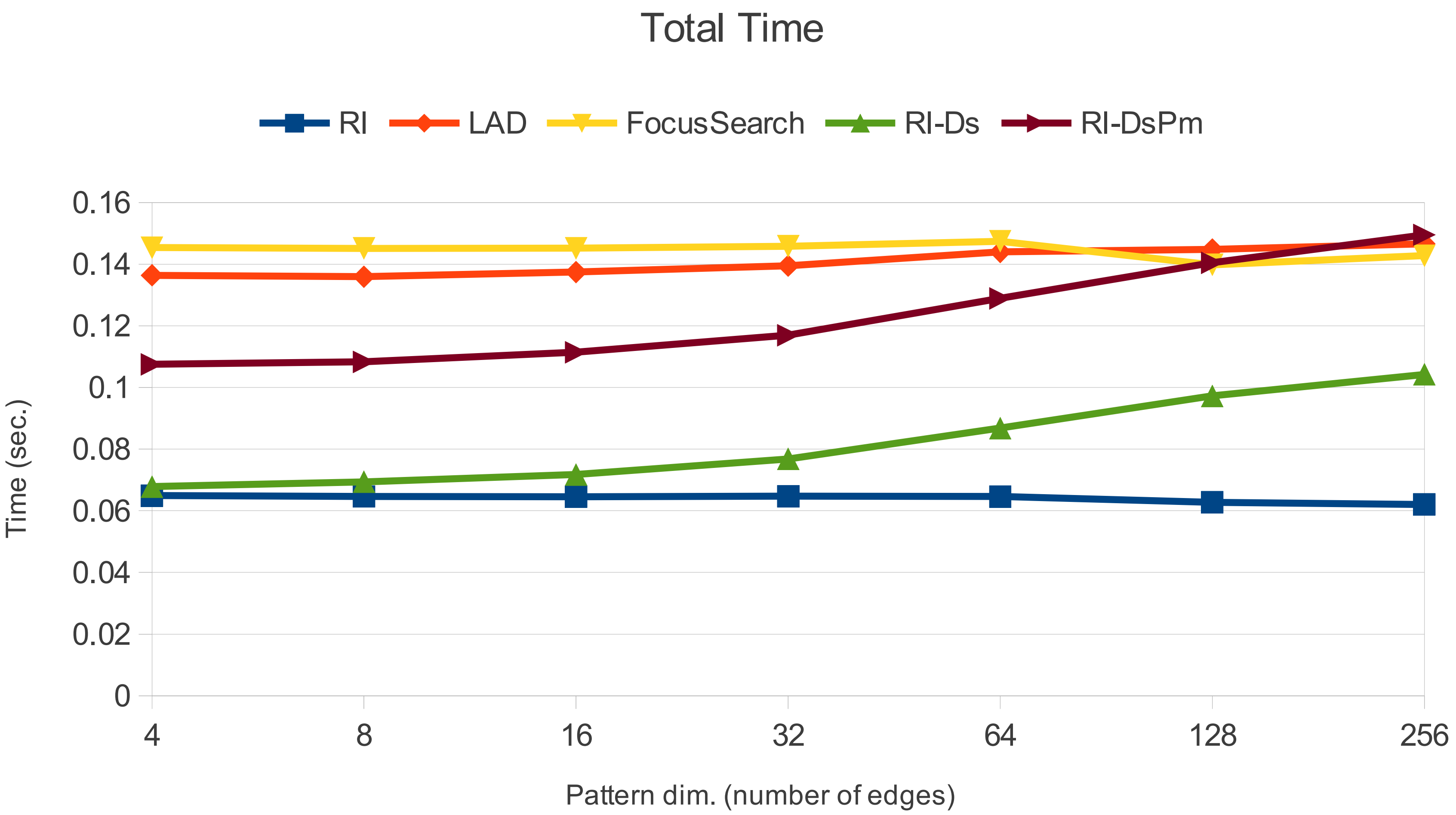

Figure 42: Averages of matching times on *PPI* dataset. The PPI networks are labeled with the names of proteins. Each vertex has a unique label. RI outperforms all other systems

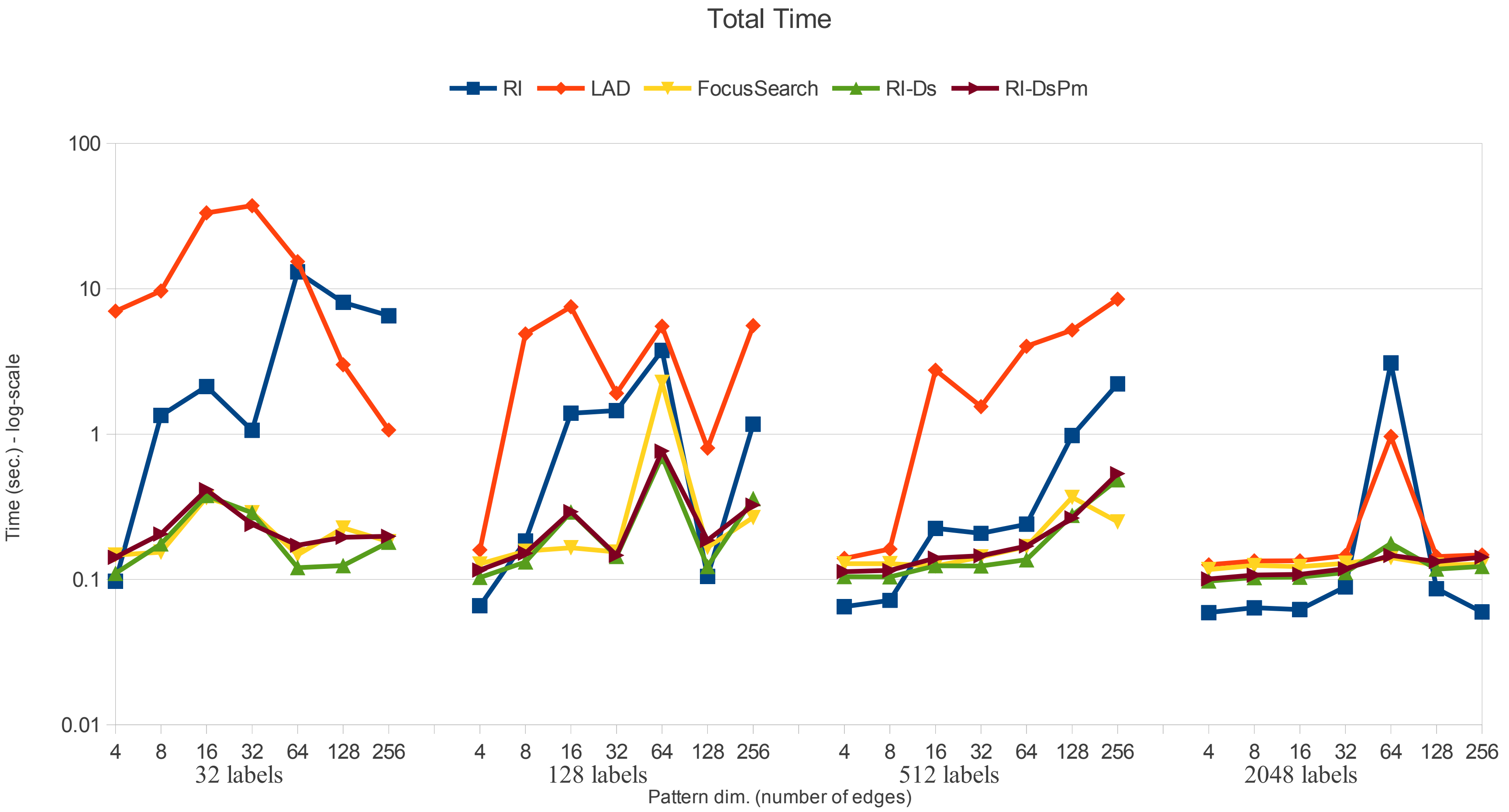

Figure 43: Averages of matching times on *PPI* dataset. The PPI networks are randomly labelled using a normal distribution. RI-Ds outperforms all other systems.

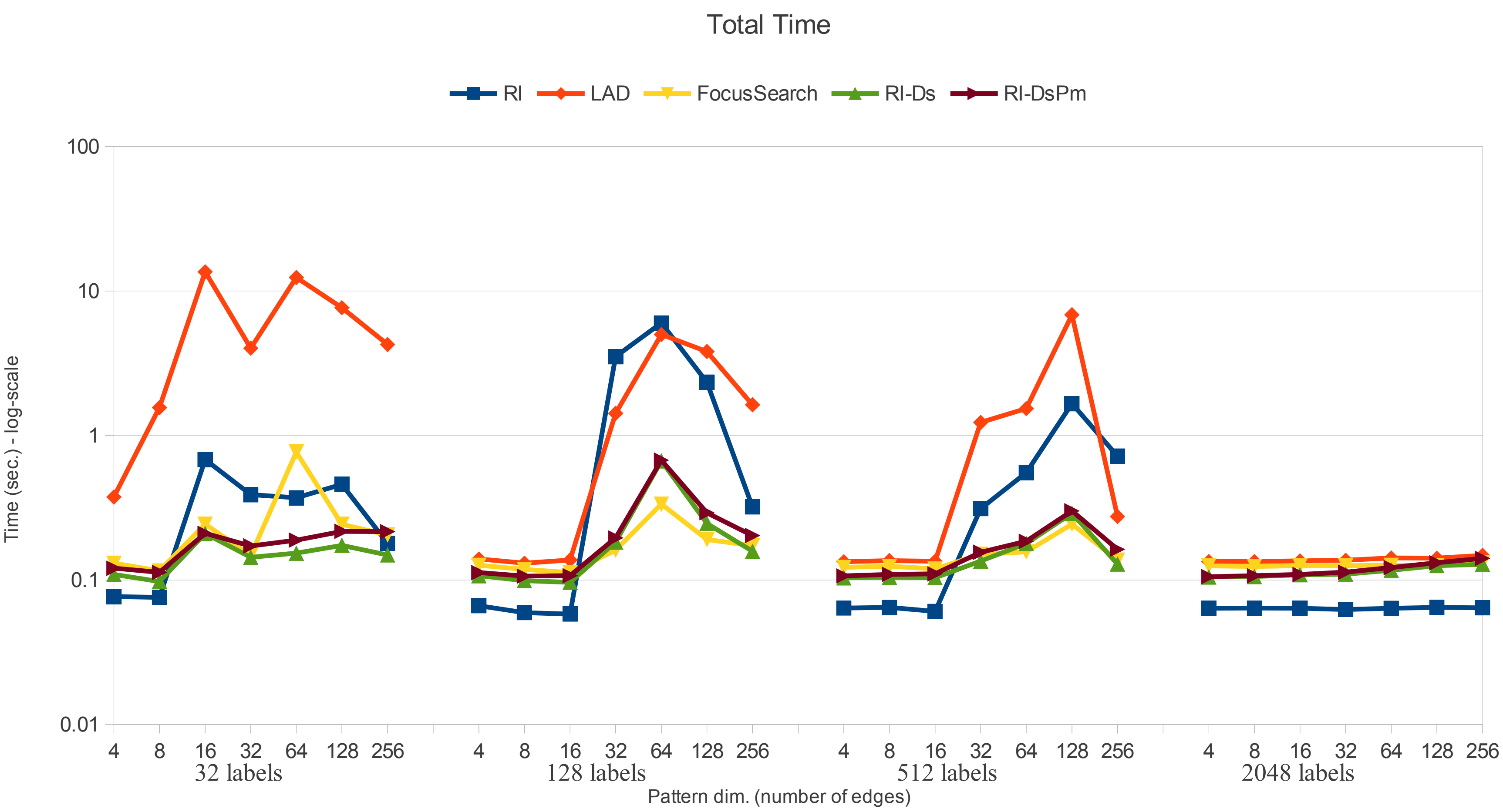

Figure 44: Averages of matching times on *PPI* dataset. The PPI networks are uniformly randomly labelled. RI-Ds outperforms all other systems.

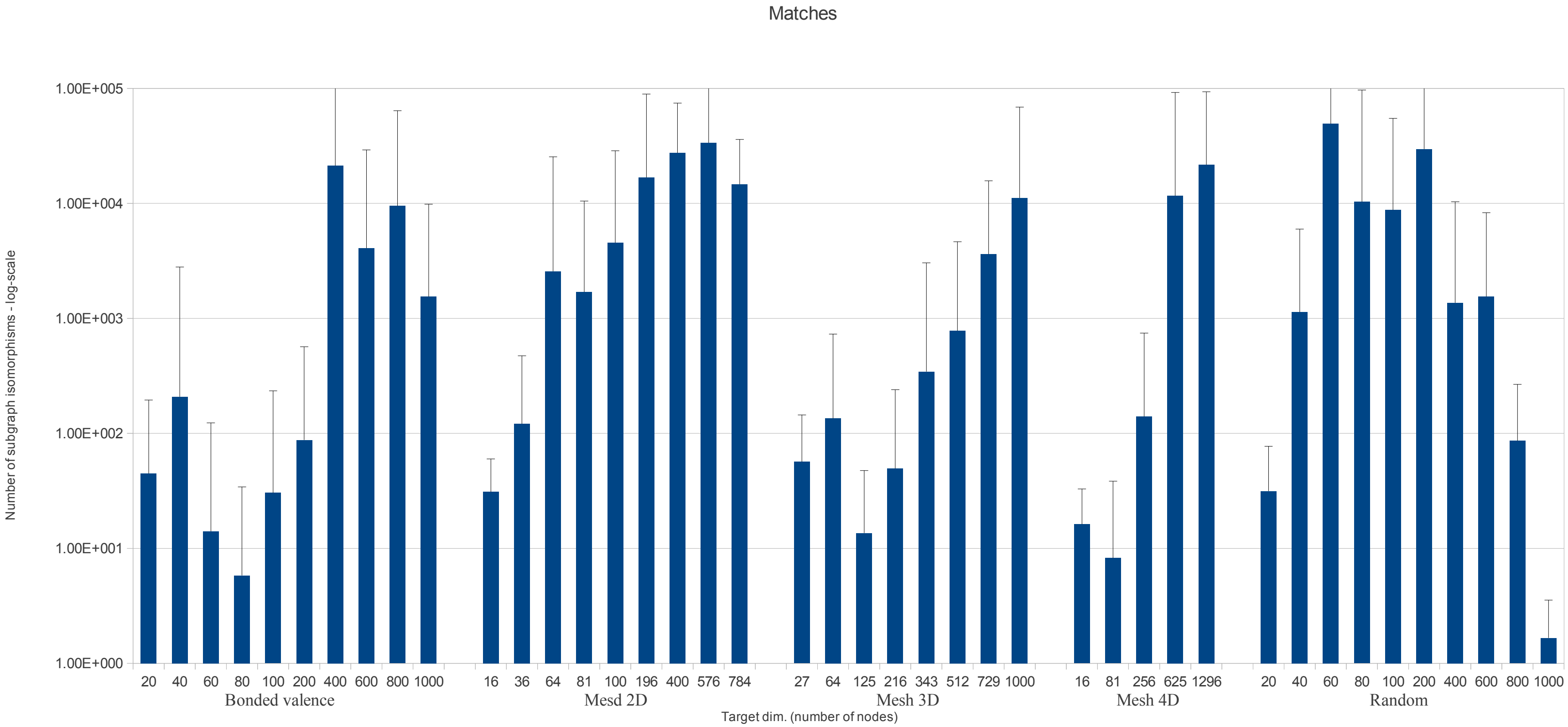

Figure 45: The number of matches on Sansone et al. dataset. Results are grouped by target type (Bounded valence, Mesh 2D, Mesh 3D, Mesh 4D, Random) and target dimension (number of vertices). Patterns come from the original dataset.

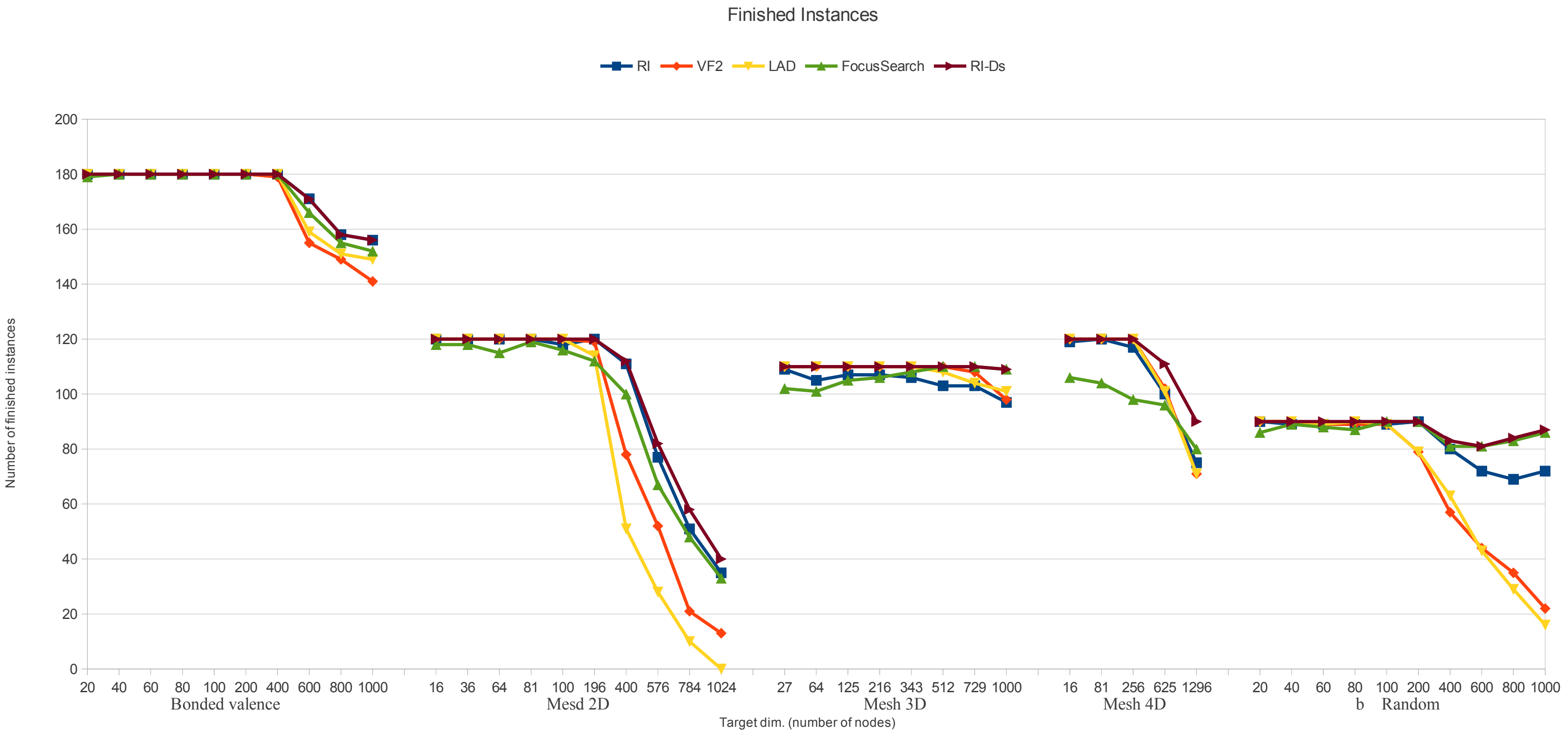

Figure 46: Number of times algorithms end before timeout.

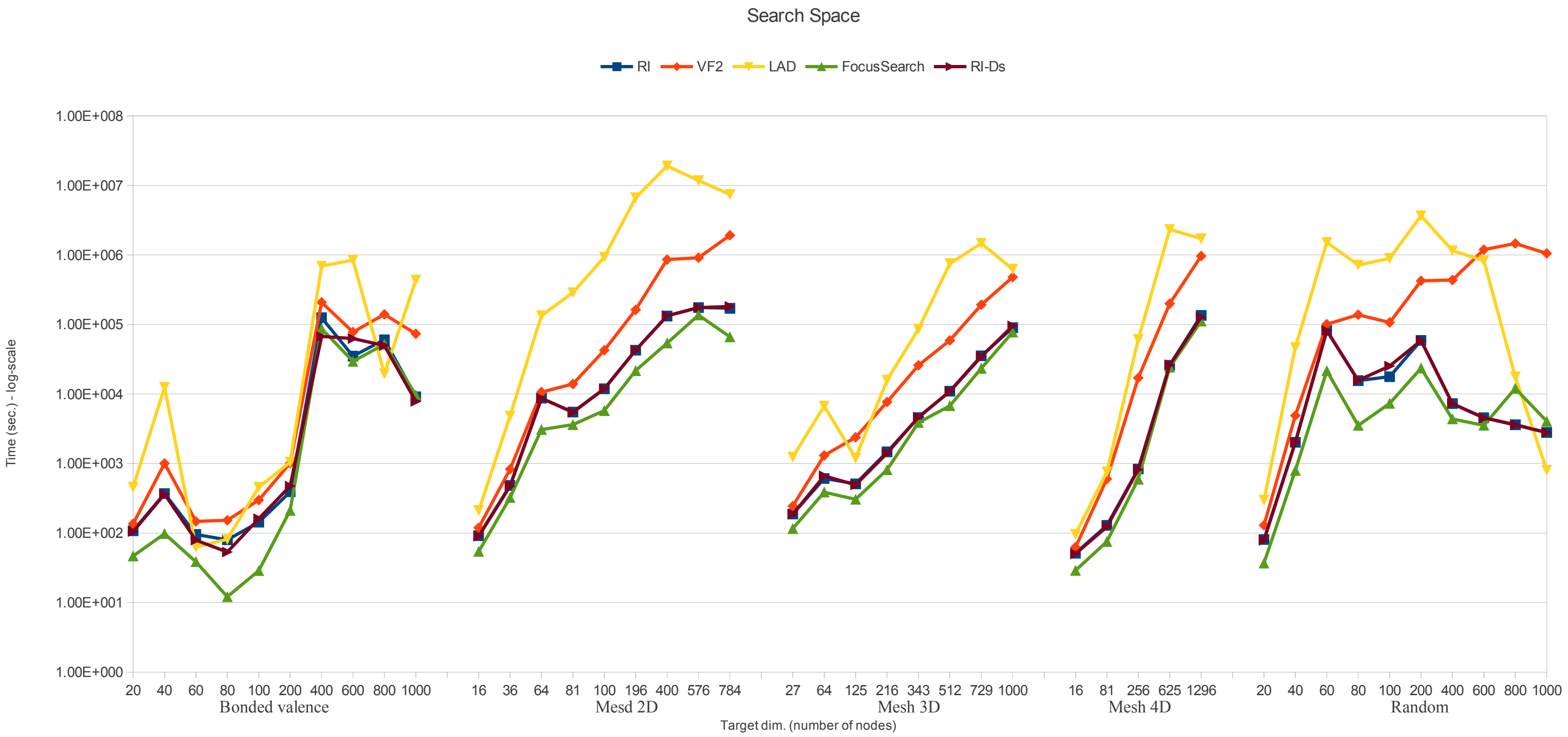

Figure 47: Averages of search spaces sizes on Sansone et al. dataset. The chart shows the number of visited nodes of the search tree by the five algorithms. Results are grouped by target type (Bounded valence, Mesh 2D, Mesh 3D, Mesh 4D, Random) and target dimension (number of vertices). Patterns come from the original dataset. On unlabeled graphs, FocusSearch does not take advantages by its prematch steps (which is based on degrees and labels neighborhood). The pruning power is due only to the reduction procedures.

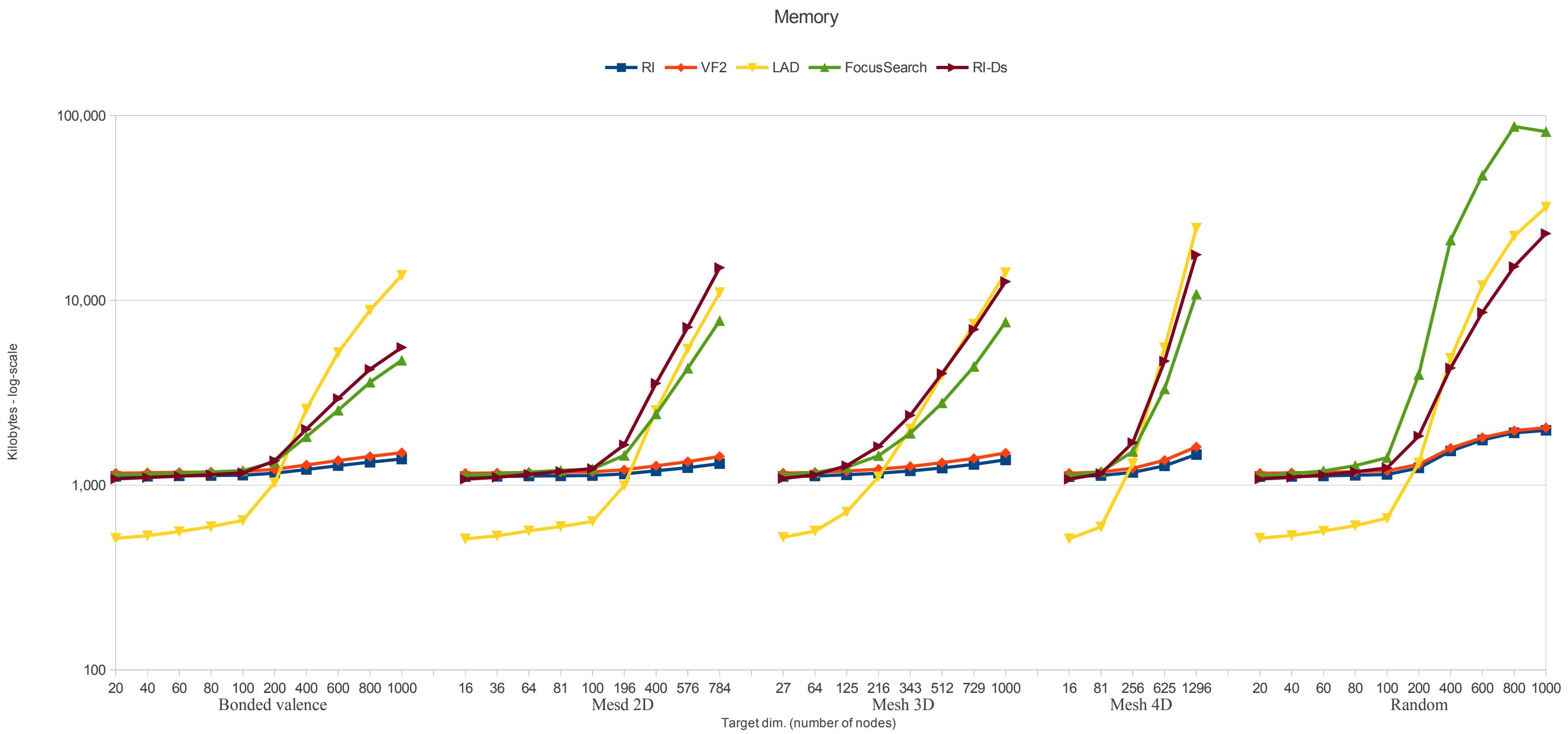

Figure 48: Memory requirement on Sansone et al. dataset. The chart measures the peak of memory usage during the executions of the algorithms. Results are grouped by target type (Bounded valence, Mesh 2D, Mesh 3D, Mesh 4D, Random) and target dimension (number of vertices). Patterns come from the original dataset. On increasing the target dimension, the domains of FocusSearch and LAD become large.

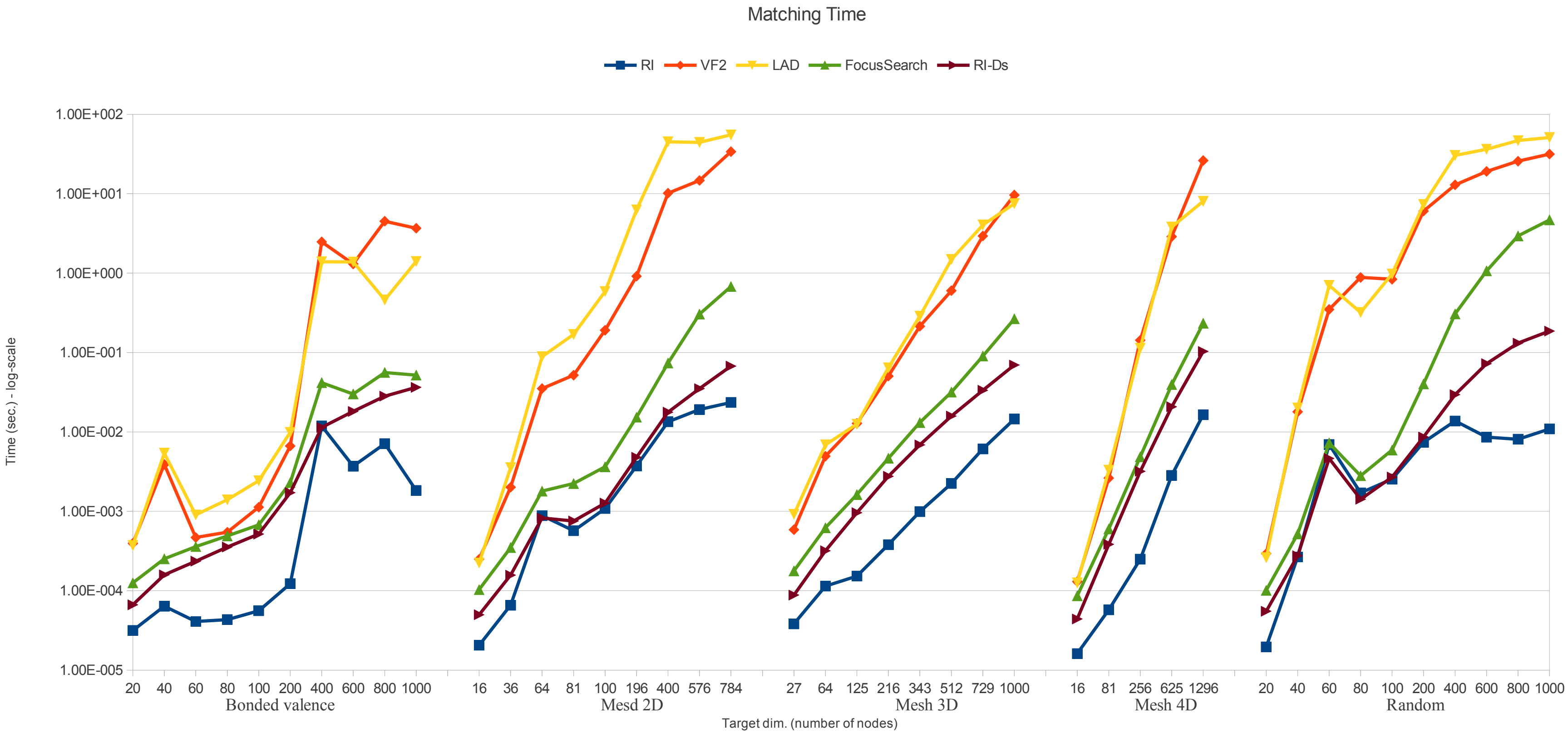

Figure 49: Matching time on Sansone et al. dataset. Results are grouped by target type (Bounded valence, Mesh 2D, Mesh 3D, Mesh 4D, Random) and target dimension (number of vertices). Patterns come from the original dataset. RI outperforms FocusSearch and LAD.

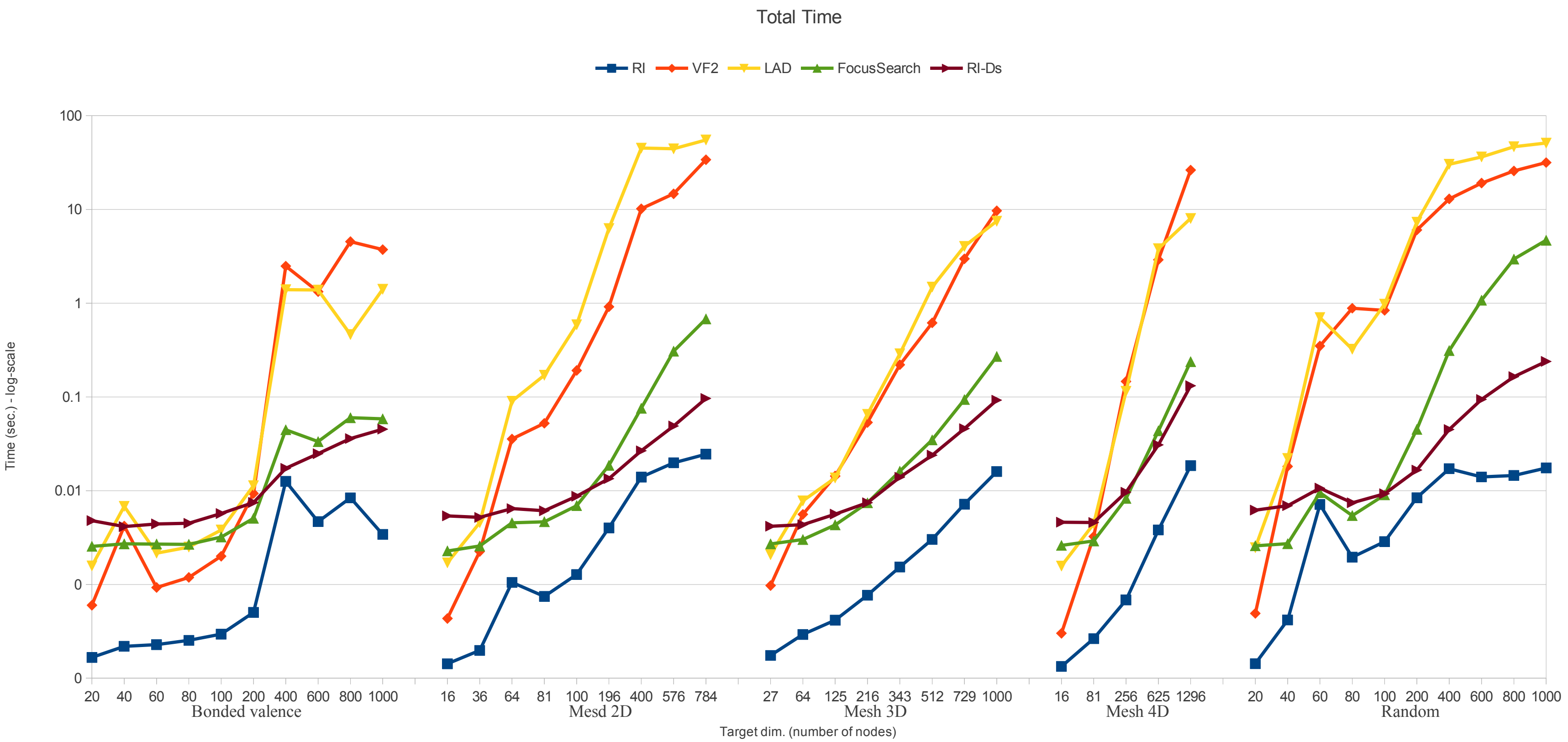

Figure 50: Total time on Sansone et al. dataset. Results are grouped by target type (Bounded valence, Mesh 2D, Mesh 3D, Mesh 4D, Random) and target dimension (number of vertices). Patterns come from the original dataset. Both the ratio between the size of the generated search space and the time needed to explore it allow RI to outperform FocusSearch and LAD.

Complexes Search in Biological Networks

The CORUM dataset[2] contains protein complexes from several species. Each complex extracted from a species is known to be highly conserved in different species. That is, proteins of a complex are high conserved in the other species via protein sequence similarity obtained from the SIMAP database[5]. This conservation does not necessarily imply that the molecular interactions within a complex in a species are also present in the other species. We test whether the implication nevertheless does apply.

We labeled the protein interaction networks used in the datasets *PPI* using the GO annotations taken from BioDBNet[4]. We extract the interactions from the species where some complexes of interest originated, and we look for all occurrences of such complexes graphs (patterns) in the other species (targets) using their known functional annotations and the similarity map computed with SIMAP. Using DAVID [6] we extracted the most relevant GO terms associated with the subunits of the complexes. The similarity map is used to select among the results the most similar to the complex patterns.

Let us use as example the complex *20S proteasome*[3] from *Mus musculus* (see Figure 52). It has 14 vertices and 182 edges. It is an essential component of the ATP-dependent proteolytic pathway in eukaryotic cells and is responsible for the degradation of most cellular proteins. The *20S proteasome* in our *PPI* dataset is high conserved in *Homo sapiens, Rattus norvegicus, Bos taurus, Xenopus tropicalis, Danio rerio,* and *Takifugu rubipres* and medium conserved in *Caenorhabditis elegans, Drosophila melanogaster* and *Saccaromyces cerevisiae*. Figure51 shows a screen shot of CORUM phylogenetic conservation of the *20S proteasome* complex.

#	Complex Name	Organism	Protein	Homo sapiens	Mus musculus	Rattus norvegicus	Bos taurus	Xenopus laevis	Danio rerio	Takifugu rubripes	Drosophila melanogaster	Caenorhabditis elegans	Arabidopsis thaliana	Oryza sativa	Neurospora crassa	Schizosaccharomyces pombe	Saccharomyces cerevisiae	Dictyostelium discoideum	Thermoplasma acidophilum	Escherichia coli	Bacillus subtilis
38	20S proteasome	Mouse																			
			Psmb1	0.93	1.00	0.98	0.94	0.83	0.85	0.84	0.52	0.36	0.47	0.48	0.41	0.46	0.42	0.39	0.20	0.02	0.05
			Psmb5	0.93	1.00	0.98	0.93	0.74	0.68	0.66	0.50	0.34	0.53	0.53	0.44	0.48	0.48	0.54	0.27	0.01	0.03
			Psma3	0.99	1.00	1.00	0.99	0.94	0.93	0.92	0.57	0.48	0.62	0.62	0.43	0.48	0.44	0.59	0.27	0.03	0.03
			Psma2	1.00	1.00	1.00	0.98	0.96	0.95	0.94	0.80	0.65	0.68	0.68	0.57	0.58	0.51	0.64	0.35	0.03	0.03
			Psmb7	0.98	1.00	0.99	0.97	0.81	0.82	0.80	0.64	0.40	0.59	0.60	0.56	0.57	0.54	0.62	0.21	0.09	0.02
			Psmb4	0.94	1.00	0.97	0.93	0.79	0.76	0.77	0.39	0.30	0.43	0.40	0.35	0.41	0.38	0.40	0.11	0.10	0.07
			Psmb6	0.93	1.00	0.97	0.92	0.80	0.76	0.74	0.57	0.45	0.46	0.49	0.46	0.53	0.54	0.64	0.20	0.05	0.00
			Psma6	1.00	1.00	1.00	1.00	0.95	0.95	0.94	0.70	0.61	0.67	0.62	0.58	0.59	0.50	0.58	0.33	0.05	0.02
			Psma4	0.99	1.00	1.00	0.99	0.97	0.97	0.96	0.70	0.58	0.60	0.61	0.61	0.57	0.52	0.59	0.32	0.05	0.04
			Psmb3	0.99	1.00	1.00	1.00	0.94	0.94	0.79	0.68	0.54	0.60	0.39	0.62	0.60	0.58	0.56	0.19	0.04	0.03
			Psmb2	0.97	1.00	0.99	0.97	0.85	0.85	0.83	0.55	0.38	0.45	0.41	0.46	0.47	0.47	0.41	0.15	0.04	0.04
			Psma1	0.98	1.00	0.99	0.98	0.94	0.90	0.90	0.52	0.55	0.48	0.49	0.52	0.57	0.53	0.64	0.31	0.03	0.03
			Psma7	0.99	1.00	0.99	1.00	0.97	0.90	0.85	0.69	0.63	0.67	0.23	0.59	0.54	0.59	0.70	0.40	0.02	0.02

Figure 51: Phylogenetic conservation of the *20S proteasome* complex.

We found that the *20S proteasome* complex interactions are preserved in all tests networks except in the network for *Xenopus tropicalis*. This may due to the lack of pattern edges or relevant vertices in the target networks. In general we observed a large number of matches in networks related to complex organisms (for example in *Homo sapiens* we found that the complex also matches with subunits specific of the *26S proteasome* complexes). Figure 53 shows a match of the *20S proteasome* complex in *Takifugu rubipres* and *Rattus norvegicus*, respectively.

References

[1] L. Cordella, P. Foggia, C. Sansone, and M. Vento. A (sub)graph isomorphism algorithm for matching large graphs. *IEEE Transactions on Pattern Analysis and Machine Intelligence*, 26(10):1367–1372, 2004.

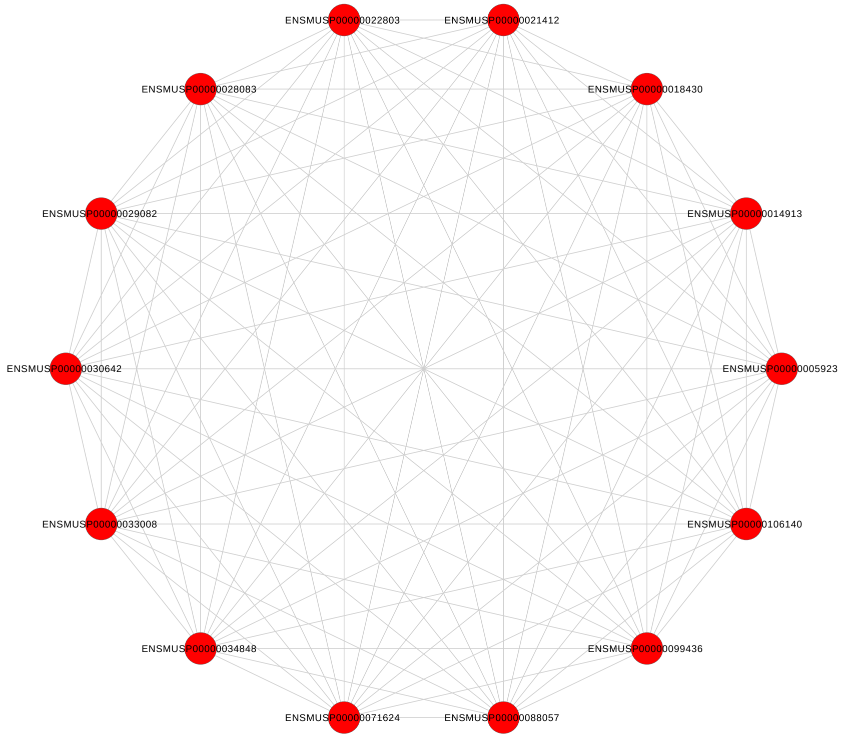

Figure 52: *20S proteasome* complex from *Mus musculus*

[2] I Dunger-Kaltenbach, G Fobo, C Frishman, G Montrone, and HW Mewes. Corum: the comprehensive resource of mammalian protein complexes. *Nucleic Acids Res.*, 2010.

[3] LA Elenich, D Nandi, AE Kent, TS McCluskey, M Cruz, MN Iyer, EC Woodward, CW Conn, AL Ochoa, DB Ginsburg, and Monaco JJ. The complete primary structure of mouse 20s proteasomes. *Immunogenetics*, 1999.

[4] U Mudunuri, A Che, M Yi, and MR Stephens. biodbnet: the biological database network. *Bioinformatics*, 25(4):555–556, 2009.

[5] T Rattei, P Tischler, S Gotz, J Jehl, MA Hoser, R Arnold, A Conesa, and MewesHW. Simapa comprehensive database of pre-calculated protein sequence similarities, domains, annotations and clusters. *Nucleic Acids Res.*, 2010.

[6] BT Sherman, DW Huang, Q Tan, Y Guo, S Bour, D Lui, R Stephens, MW Baseler, C Lane, and RA Lempicki. David knowledgebase: a gene-centered database integrating heterogeneous gene annotation resources to facilitate high-throughput gene functional analysis. *BMC Bioinformatics*, 2007.

[7] C. Solnon. Alldifferent-based filtering for subgraph isomorphism. *Artificial Intelligence*, 174:850–864, 2010.

[8] J. R. Ullmann. Bit-vector algorithms for binary constraint satisfaction and subgraph isomorphism. *J. Exp. Algorithmics*, 15:1.6:1.1–1.6:1.64, February 2011.

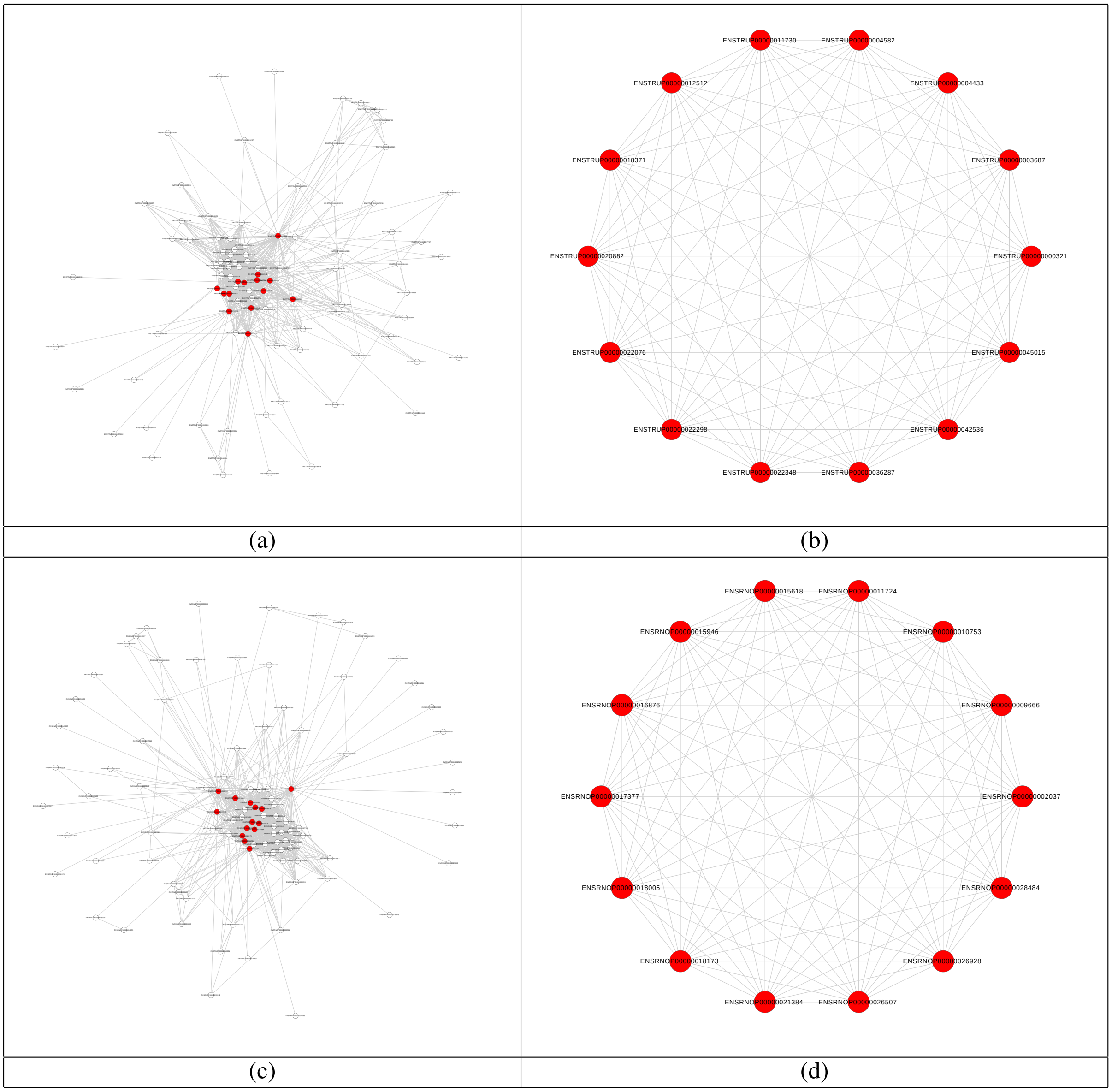

Figure 53: (a) and (b) show the *20S proteasome* complex occurrence in *Takifugu rubipres*. (c) and (d) show the *20S proteasome* complex occurrence in *Rattus norvegicus*. (b) and (c) depict the occurrences with a partial view of the target networks.
