# Supplementary material for: A subgraph isomorphism algorithm and its application to biochemical data
Source: BMC Bioinformatics. 2013 Apr 22;14(Suppl 7):S13. doi: 10.1186/1471-2105-14-S7-S13 (PMC3633016; doi:10.1186/1471-2105-14-S7-S13)
Supplement: Additional file 2 — Average matching and total time performances on AIDS and PDBS datasets. For PDBSv3, tests are grouped with respect to pattern densities. For each algorithm, the average of its result values (expressed in sec) is reported together with the standard deviation (see Additional File 1 for more detailed results). The best algorithm is highlighted in bold. [file 1471-2105-14-S7-S13-S2.pdf]

Dataset	Measurement	RI	VF2	LAD	FocusSearch
AIDS	Matching	**0.529**(±0.386)	8.449(±6.447)	1.615(±1.989)	2.404(±1.338)
	Total	**3.448**(±0.391)	16.678(±6.454)	4.697(±1.883)	7.212(±1.105)
PDBSv1	Matching	**0.040**(±0.112)	4.476(±11.136)	1.747(±5.104)	0.224(±0.695)
	Total	**0.049**(±0.120)	6.438(±15.581)	1.812(±5.213)	0.231(±0.612)
PDBSv2	Matching	**0.008**(±0.010)	1.653(±2.402)	0.621(±0.953)	0.130(±0.148)
	Total	**0.014**(±0.013)	2.252(±2.915)	0.640(±0.961)	0.138(±0.142)
PDBSv3 Dense Patterns	Matching	**0.000**(±0.001)	0.027(±0.055)	0.002(±0.001)	0.002(±0.001)
	Total	**0.005**(±0.002)	0.049(±0.063)	0.004(±0.001)	0.006(±0.002)
PDBSv3 Semi-dense Patterns	Matching	0.019(±0.052)	6.227(±8.810)	0.093(±0.371)	**0.006**(±0.013)
	Total	0.023(±0.053)	6.248(±8.813)	0.095(±0.370)	**0.009**(±0.014)
PDBSv3 Sparse Patterns	Matching	1.136(±1.330)	23.221(±21.364)	3.417(±4.803)	**0.231**(±0.344)
	Total	1.142(±1.330)	23.255(±21.367)	3.420(±4.802)	**0.236**(±0.343)
