# Supplementary material for: A subgraph isomorphism algorithm and its application to biochemical data
Source: BMC Bioinformatics. 2013 Apr 22;14(Suppl 7):S13. doi: 10.1186/1471-2105-14-S7-S13 (PMC3633016; doi:10.1186/1471-2105-14-S7-S13)
Supplement: Additional file 3 — Average space and memory requirements on AIDS and PDBS datasets. For PDBSv3, tests are grouped with respect to pattern densities. For each algorithm, the average of its result values (expressed as the number of space's nodes and kilobytes) is reported together with the standard deviation (see Additional File 1 for more detailed results). The best algorithm is highlighted in bold. [file 1471-2105-14-S7-S13-S3.pdf]

| Dataset | Measurement | RI | VF2 | LAD | FocusSearch |
|---|---|---|---|---|---|
| *AIDS* | Space | 2513149.2(±2343680.6) | 3180536.8(±2305950.4) | **273099.9**(±413978.4) | 865652.4(±1097407.6) |
| | Memory | **85443.6**(±291.0) | 393179.7(±188.5) | 891944.0(±55160.7) | 230816.3(±6371.3) |
| *PDBSv1* | Space | 267551.5(±746512.4) | 354017.7(±1006772.6) | 76184.5(±252165.3) | 93060.1(±291.0) |
| | Memory | **2117.4** (±1133.0) | 2184.6(±1149.6) | 60501.3(±139329.8) | 17478.1(±44098.4) |
| *PDBSv2* | Space | 48350.7(±67986.0) | 90819.4(±131760.1) | **9508.0**(±17825.1) | 20497.0(±39395,7) |
| | Memory | **1759.4**(±301.0) | 2016.4(±391.0) | 21726.3(±16840.0) | 5735.0(±2974.4) |
| *PDBSv3* Dense Patterns | Space | 216.9(±268.3) | 1259.8(±2311.1) | 5.6(±8.3) | **4.8**(±20.9) |
| | Memory | 1421.3(±104.5) | 1651.4(±164.5) | **1134.8**(±278.4) | 1505.1(±98.4) |
| *PDBSv3* Semi-dense Patterns | Space | 26157.7(±81705.1) | 262101.9(±374101.4) | 8594.0(±38699.1) | **7032.5**(±29940.4) |
| | Memory | 1396.8(±113.5) | 1614.2(±178.5) | **1207.6**(±326.2) | 1511.7(±124.5) |
| *PDBSv3* Sparse Patterns | Space | 1263241.8(±1455655.3) | 1456893.3(±1383478.8) | 635652.2(±920536.4) | **568985.5**(±889671.1) |
| | Memory | 1511.1(±121.5) | 1798.6(±190.7) | **1418.6**(±298.8) | 1626.2(±119.5) |
