# Supplementary material for: A subgraph isomorphism algorithm and its application to biochemical data
Source: BMC Bioinformatics. 2013 Apr 22;14(Suppl 7):S13. doi: 10.1186/1471-2105-14-S7-S13 (PMC3633016; doi:10.1186/1471-2105-14-S7-S13)
Supplement: Additional file 4 — Average matching and total time performances on Sansone et al dataset. Tests are grouped with respect to target topologies. For each algorithm, the average of its result values (expressed in sec) is reported together with the standard deviation (see Additional File 1 for more detailed results). The best algorithm is highlighted in bold. [file 1471-2105-14-S7-S13-S4.pdf]

Sansone et al. dataset	Measurement	RI	RI-Ds	VF2	LAD	FocusSearch
bounded	Matching	**0.002**(±0.014)	0.010(±0.012)	1.200(±6.578)	0.466(±3.745)	0.018(±0.012)
	Total	**0.003**(±0.014)	0.015(±0.015)	1.210(±6.578)	0.467(±3.723)	0.022(±0.015)
m2D	Matching	**0.007**(±0.012)	0.014(±0.009)	6.650(±12.299)	16.800(±21.899)	0.120(±0.009)
	Total	**0.007**(±0.012)	0.024(±0.012)	6.660(±12.299)	16.842(±21.878)	0.122(±0.012)
m3D	Matching	**0.003**(±0.008)	0.016(±0.006)	1.680(±6.631)	1.670(±4.986)	0.051(±0.006)
	Total	**0.004**(±0.008)	0.025(±0.010)	1.690(±6.630)	1.675(±4.959)	0.054(±0.010)
m4D	Matching	**0.004**(±0.009)	0.026(±0.029)	5.840(±13.405)	2.390(±13.581)	0.056(±0.029)
	Total	**0.005**(±0.010)	0.036(±0.032)	5.860(±13.407)	2.390(±13.466)	0.059(±0.032)
random	Matching	**0.006**(±0.011)	0.044(±0.011)	9.730(±10.499)	17.400(±13.440)	0.904(±0.022)
	Total	**0.008**(±0.012)	0.060(±0.024)	9.750(±10.499)	17.363(±13.362)	0.910(±0.024)
