# Supplementary material for: A subgraph isomorphism algorithm and its application to biochemical data
Source: BMC Bioinformatics. 2013 Apr 22;14(Suppl 7):S13. doi: 10.1186/1471-2105-14-S7-S13 (PMC3633016; doi:10.1186/1471-2105-14-S7-S13)
Supplement: Additional file 5 — Average space and memory requirements on Sansone et al dataset. Tests are grouped with respect to target topologies (see Additional File 1 for more detailed results). For each algorithm, the average of its result values (expressed as the number of space's nodes and kilobytes) is reported together with the standard deviation. The best algorithm is highlighted in bold. [file 1471-2105-14-S7-S13-S5.pdf]

Sansone et al. dataset	Measurement	RI	RI-Ds	VF2	LAD	FocusSearch
bounded	Space	23075.3(±144186.4)	18875.4(±129865.1)	50251.0(±292235.6)	200579.3(±2003484.9)	**17825.9**(±140249.8)
	Memory	**1197.5**(±23.5)	2170.6(±576.2)	1264.9(±24.5)	3419.2(±1187.1)	1986.2(±378.0)
m2D	Space	60981.4(±126743.4)	62225.1(±134576.4)	435275.2(±886849.8)	5138548.6(±9522834.3)	**32429.8**(±111715.1)
	Memory	**1165.1**(±11.9)	3681.8(±1002.7)	1233.0(±16.8)	2536.5(±969.1)	2422.3(±441.0)
m3D	Space	17954.6(±66174.3)	18514.9(±59120.5)	96122.8(±348596.4)	369750.1(±2228839.7)	**14153.5**(±65491.0)
	Memory	**1203.0**(±15.3)	3887.8(±1014.3)	1276.2(±21.3)	3803.2(±1456.9)	2712.7(±511.8)
m4D	Space	32346.0(±48158.6)	30922.6(±84468.8)	236478.5(±678002.9)	821218.5(±3265606.9)	**27288.8**(±42011.9)
	Memory	**1229.5**(±146.2)	5252.6(±927.9)	1308.5(±133.1)	6503.2(±4262.6)	3586.2(±22427.2)
random	Space	19454.3(±84467.6)	20192.5(±84468.8)	492009.7(±777867.1)	883592.4(±2747329.3)	**8090.0**(±28369.8)
	Memory	**1404.1**(±130.1)	5870.9(±686.6)	1457.2(±117.0)	7522.8(±2554.0)	24777.5(±15858.3)
