# Supplementary material for: A subgraph isomorphism algorithm and its application to biochemical data
Source: BMC Bioinformatics. 2013 Apr 22;14(Suppl 7):S13. doi: 10.1186/1471-2105-14-S7-S13 (PMC3633016; doi:10.1186/1471-2105-14-S7-S13)
Supplement: Additional file 6 — Average matching and total time performances on Graemlin dataset. Tests are grouped with respect to the number of labels as shown in the plots (see Additional File 1 for more detailed results). For each algorithm, the average of its result values (expressed in sec) is reported together with the standard deviation. The best algorithm is highlighted in bold. [file 1471-2105-14-S7-S13-S6.pdf]

GRAEMLIN dataset	Measurement	RI	RI-Ds	RI-DsPm	VF2	LAD	FocusSearch
32 labels	Matching	0.108**(±0.513)**	**0.025(±0.094)**	0.050**(±0.101)**	2.879**(±10.164)**	1.416**(±7.373)**	0.034**(±0.045)**
	Total	0.149**(±0.525)**	**0.064(±0.113)**	0.103**(±0.132)**	3.604**(±10.335)**	1.464**(±7.384)**	0.092**(±0.083)**
512 labels	Matching	0.077**(±0.471)**	**0.011(±0.030)**	0.019**(±0.031)**	1.520**(±6.980)**	0.038**(±0.293)**	0.019**(±0.022)**
	Total	0.124**(±0.490)**	**0.057(±0.060)**	0.080**(±0.072)**	2.374**(±7.432)**	0.095**(±0.321)**	0.088**(±0.078)**
2048 labels	Matching	0.009**(±0.104)**	**0.008(±0.046)**	0.014**(±0.014)**	0.368**(±3.643)**	0.007**(±0.060)**	0.017**(±0.021)**
	Total	0.058**(±0.129)**	**0.056(±0.077)**	0.077**(±0.058)**	1.243**(±4.145)**	0.066**(±0.095)**	0.089**(±0.074)**
unique labels	Matching	0.000**(±0.000)**	**0.006(±0.001)**	0.012**(±0.003)**	0.004**(±0.001)**	0.004**(±0.001)**	0.016**(±0.003)**
	Total	0.050**(±0.024)**	**0.055(±0.025)**	0.075**(±0.030)**	0.884**(±0.489)**	0.063**(±0.030)**	0.090**(±0.030)**
