# Supplementary material for: A subgraph isomorphism algorithm and its application to biochemical data
Source: BMC Bioinformatics. 2013 Apr 22;14(Suppl 7):S13. doi: 10.1186/1471-2105-14-S7-S13 (PMC3633016; doi:10.1186/1471-2105-14-S7-S13)
Supplement: Additional file 7 — Average space and memory requirements on Graemlin dataset. Tests are grouped with respect to the number of labels as shown in the plots (see Additional File 1 for more detailed results). For each algorithm, the average of its result values (expressed as the number of space's nodes and kilobytes) is reported together with the standard deviation. The best algorithm is highlighted in bold. [file 1471-2105-14-S7-S13-S7.pdf]

| GRAEMLIN dataset | Measurement | RI | RI-Ds | RI-DsPm | VF2 | LAD | FocusSearch |
|---|---|---|---|---|---|---|---|
| *32 labels* | Space | 62786.7(±299865.9) | 60369.9(±286824.6) | 55436.3(±262247.0) | 70545.6(±306445.6) | 480395.1(±2330735.6) | **5991.0**(±22584.5) |
| | Memory | **3316.3**(±1887.8) | 3631.2(±2073.9) | 16843.8(±8788.8) | 5551.3(±3905.3) | 14287.2(±11973.2) | 5598.1(±3641.8) |
| *512 labels* | Space | 8647.6(±46467.2) | 3530.0(±20102.8) | 1883.1(±13353.5) | 12199.8(±59459.2) | 1266.6(±21471.7) | **566.8**(±3221.7) |
| | Memory | **3706.5**(±2161.6) | 3875.0(±2238.9) | 17124.6(±8953.9) | 6359.9(±4478.9) | 16496.5(±13993.9) | 5491.9(±3587.2) |
| *2048 labels* | Space | 945.7(±29748.6) | 439.7(±55878.7) | 146.6(±14344.5) | 2213.9(±35293.2) | 83.1(±9020.2) | **43.1**(±6892.2) |
| | Memory | **3780.4**(±2153.6) | 3932.6(±2210.9) | 17247.3(±8648.6) | 6513.6(±4465.0) | 16652.8(±13672.7) | 5537.5(±3393.5) |
| *unique labels* | Space | 42.6(±4.1) | 40.9(±4.1) | 40.9(±4.1) | 41.9(±4.1) | 2.0(±0.0) | **0.0**(±0.0) |
| | Memory | **3799.6**(±1262.7) | 3932.5(±1284.5) | 17225.0(±4980.2) | 6554.2(±2617.9) | 16347.2(±7941.3) | 5373.1(±1927.7) |
