# Supplementary material for: A subgraph isomorphism algorithm and its application to biochemical data
Source: BMC Bioinformatics. 2013 Apr 22;14(Suppl 7):S13. doi: 10.1186/1471-2105-14-S7-S13 (PMC3633016; doi:10.1186/1471-2105-14-S7-S13)
Supplement: Additional file 8 — Average matching and total time performances on PPI dataset. Tests are grouped with respect to the number of labels and label distributions as shown in the plots (see Additional File 1 for more detailed results). For each algorithm, the average of its result values is reported together with the standard deviation. The best algorithm is highlighted in bold. [file 1471-2105-14-S7-S13-S8.pdf]

PPI network dataset	Measurement	RI	RI-Ds	RI-Ds-Pm	LAD	FocusSearch
Unique labels	Matching	**0.001**(±0.000)	0.018(±0.011)	0.024(±0.013)	0.009(±0.004)	0.032(±0.014)
	Total	**0.064**(±0.045)	0.082(±0.047)	0.123(±0.055)	0.141(±0.064)	0.144(±0.073)
Labels with skewed distribution						
32	Matching	4.564(±9.099)	**0.116**(±0.221)	0.142(±0.186)	15.120(±26.939)	0.150(±0.167)
	Total	4.609(±9.102)	**0.197**(±0.220)	0.224(±0.201)	15.230(±26.936)	0.216(±0.163)
512	Matching	1.719(±7.570)	0.170(±0.211)	0.148(±0.186)	1.522(±7.089)	**0.127**(±0.387)
	Total	1.776(±7.585)	0.263(±0.220)	0.242(±0.201)	1.647(±7.107)	**0.210**(±0.404)
2048	Matching	0.441(±3.337)	**0.022**(±0.077)	0.026(±0.037)	0.129(±0.910)	0.038(±0.030)
	Total	0.501(±3.368)	**0.119**(±0.115)	0.122(±0.077)	0.257(±0.954)	0.127(±0.081)
Labels with uniform distribution						
32	Matching	0.268(±0.666)	**0.056**(±0.118)	0.089(±0.118)	6.141(±20.413)	0.191(±0.459)
	Total	0.319(±0.676)	**0.148**(±0.137)	0.177(±0.141)	6.259(±20.420)	0.265(±0.472)
256	Matching	0.431(±1.894)	**0.052**(±0.173)	0.066(±0.178)	1.342(±6.250)	0.065(±0.133)
	Total	0.490(±1.915)	**0.149**(±0.201)	0.162(±0.207)	1.470(±6.278)	0.151(±0.165)
2048	Matching	0.002(±0.007)	0.013(±0.007)	0.020(±0.011)	**0.010**(±0.007)	0.035(±0.016)
	Total	0.064(±0.048)	0.114(±0.052)	0.118(±0.056)	**0.139**(±0.065)	0.126(±0.075)
