# Supplementary material for: A subgraph isomorphism algorithm and its application to biochemical data
Source: BMC Bioinformatics. 2013 Apr 22;14(Suppl 7):S13. doi: 10.1186/1471-2105-14-S7-S13 (PMC3633016; doi:10.1186/1471-2105-14-S7-S13)
Supplement: Additional file 9 — Average space and memory requirements on PPI dataset. For each dataset, tests are grouped with respect to respect to the number of labels and label distributions as shown in the plots (see Additional File 1 for more detailed results). For each algorithm, the average of its result values (expressed as the number of space's nodes and kilobytes) is reported together with the standard deviation. The best algorithm is highlighted in bold. [file 1471-2105-14-S7-S13-S9.pdf]

PPI network dataset	Measurement	RI	RI-Ds	RI-Ds-Pm	LAD	FocusSearch
unique labels	Space	32.2(±14.8)	225.6(±14.8)	225.6(±14.8)	2.0(±0.0)	**0.0**(±0.0)
	Memory	**4873.2**(±2273.0)	5201.5(±2294.1)	37098.4(±9310.1)	69584.4(±36417.9)	16143.6(±7439.6)
Labels with skewed distribution						
32	Space	1211033.9(±2016492.2)	2500732.9(±617967.4)	2370084.3(±608787.5)	1670880.3(±4121724.3)	**67329.1**(±122938.1)
	Memory	**3997.0**(±1025.6)	**3997.0**(±8729.3)	38651.3(±9058.4)	66502.3(±28711.4)	17174.0(±6504.2)
512	Space	476352.8(±2049546.4)	1437629.6(±945342.0)	1275656.2(±808322.3)	112381.1(±499665.1)	**72136.7**(±327591.8)
	Memory	**4530.2**(±1991.3)	**4530.2**(±9247.4)	37547.0(±9548.6)	71092.2(±36261.5)	16965.5(±7787.0)
2048	Space	28564.8(±215397.3)	36633.3(±39000.5)	25916.7(±27735.7)	2763.0(±20853.2)	**1755.2**(±13274.3)
	Memory	**4680.5**(±2190.6)	**4680.5**(±9245.3)	37042.5(±9402.6)	70360.8(±36727.5)	16332.2(±7588.5)
Labese with uniform distribution						
32	Space	143365.8(±377057.7)	918815.3(±364689.4)	862651.5(±349474.4)	825666.3(±3415048.9)	**100874.8**(±333778.9)
	Memory	**4275.7**(±1585.5)	**4275.7**(±9041.9)	38126.5(±9518.0)	69011.0(±33673.0)	17427.1(±7521.3)
256	Space	110747.4(±518291.5)	332790.6(±215013.0)	332656.0(±229833.1)	27633.6(±119970.4)	**17512.8**(±82031.3)
	Memory	**4644.2**(±2106.2)	**4644.2**(±9500.9)	37645.1(±9443.3)	72311.6(±37727.9)	17010.0(±7973.8)
2048	Space	98.0(±251.3)	470.8(±184.6)	319.2(±84.2)	13.7(±64.6)	**6.5**(±38.3)
	Memory	**4748.9**(±2252.5)	**4748.9**(±9295.1)	37123.7(±9412.7)	70498.3(±36882.4)	16342.7(±7659.7)
